# MUC16 and TP53 family co-regulate tumor-stromal heterogeneity in pancreatic adenocarcinoma

**DOI:** 10.3389/fonc.2023.1073820

**Published:** 2023-02-03

**Authors:** Ramakanth Chirravuri-Venkata, Vi Dam, Rama Krishna Nimmakayala, Zahraa Wajih Alsafwani, Namita Bhyravbhatla, Imayavaramban Lakshmanan, Moorthy P. Ponnusamy, Sushil Kumar, Maneesh Jain, Dario Ghersi, Surinder K. Batra

**Affiliations:** ^1^ Department of Biochemistry and Molecular Biology, University of Nebraska Medical Center, Omaha, NE, United States; ^2^ School of Interdisciplinary Informatics, University of Nebraska, Omaha, NE, United States; ^3^ Eppley Institute for Research in Cancer and Allied Diseases and Buffett Cancer Center, University of Nebraska Medical Center, Omaha, NE, United States

**Keywords:** MUC16, metastasis, tumor microenvironment, mouse model, TP53 (p53), mesothelial

## Abstract

MUC16/CA125 is one of the few oldest cancer biomarkers still used in current clinical practice. As mesothelium is an abundant source of MUC16 and a major contributor to stromal heterogeneity in PDAC, we investigated the regulation of MUC16 in tumor and stromal compartments individually. The trajectories constructed using the single-cell transcriptomes of stromal cells from KPC tumors demonstrated continuity in the trajectory path between MUC16-expressing mesothelial cells and other CAF subsets. Further, the tumor tissues of MUC16 whole-body knockout (KPCM) showed dysregulation in the markers of actomyosin assembly and fibroblast differentiation (iCAF and myCAF), indicating that MUC16 has an extra-tumoral role in controlling CAF differentiation. Although we found mesothelium-derivative stromal cells to be bystanders in normal pancreas, the proportion of these cells was higher in invasive PDAC, particularly in TP53 deficient tumors. Moreover, we also detail the regulation of MUC16, KRAS, and SOX9 by TP53 family members (TP53 and TP63) using multi-omics data from knockout models, PDAC cell lines, and human PDAC tissues.

## Introduction

The advent of mucin 16 (MUC16/CA125) as an Ovarian Cancer biomarker in the 1980s is a major milestone in the biomarker research arena (1). The clinical utility of MUC16 as a diagnostic aid for risk stratification, early detection, and differential diagnosis is still relevant to contemporary cancer diagnostics. Transmembrane mucins (MUC1 and MUC16) support cancer progression by altering homozygous and heterozygous cell-cell adhesion potential (2). MUC16, in particular, is integral in regulating complex processes like cell-cell junction integrity, cytoskeletal structure, signal transduction, and invasion process (3, 4). While some literature demonstrates the colocalization of MUC16 with cytokeratins in the apical portions of mucociliary epithelium, the findings elsewhere demonstrate its overexpression during keratolysis and cornification (5, 6). MUC16 expression is not just specific to epithelial cells but is also found in other cell types like fibroblasts and mesothelial cells of visceral and reproductive organs (5, 7).

The C-terminal portion of the MUC16 protein undergoes protease-mediated cleavage by Type 1 matrix-metalloproteinases. Its circulation levels often correlate with the magnitude of connective tissue involvement during cancer progression and surgical recoveries (8, 9). The multipotent mesothelial cells support connective tissue development by acting as a major source of collagen-producing cells and have been recently recognized as key effectors in cycling-recycling events in the normal pancreas as well as PDAC progression (10, 11). The cell lineage studies demonstrate that the mesothelial-derived lineages confer intrinsic regenerative potential to several organs, including the pancreas, with little to no intervention from neural crest or circulating cells (12). It is important to note that the constitutive expression and secretion of MUC16 by mesothelial cells is several-fold higher than other cell types, including ovarian cancer cells (13). The conditional knockout of the mesothelial marker, Wilms’ tumor 1 (WT1), a putative transcriptional regulator of MUC16, results in multi-organ failure with loss of pancreatic exocrine glands (14). Although WT1 regulates embryonic development and tissue maintenance, its signaling pathways are entangled with known oncogenes and tumor suppressors (TP63, TP53, SPP1) (15, 16).

Histologically, most pancreatic tumors show a spectrum of ductal hyperplasia-carcinoma to squamous metaplasia, giving rise to speculation that squamous carcinomas are likely a result of a metaplastic change of adenocarcinoma (17). Due to the role of TP53 family members like TP63 in the squamous reprogramming of adenocarcinomas, many studies have attempted to deconvolute isoform-specific regulation of TP63 (18). It should be noted that TP63 often works in consort with TP53 to either allow DNA-damage-dependent survival or growth inhibition (19). TP53 alterations themselves change the splicing of TP63; therefore, the high predisposition of TP53 alterations in PDAC tumors may facilitate TP63-mediated molecular reprogramming of the tumors. Further, TP63 also causes global shifts in the stromal landscape with a more evident infiltration of neutrophils and inflammatory CAFs (20).

In PDAC, the infiltrating tumor cells show high expression of MUC16 relative to matched PanIN‐3 cells, correlating with tumor size, serosal invasion, and lymph node metastasis (3). The expression of MUC16 is mostly discussed in the context of tumor epithelial cells in PDAC. However, the infiltration of mesothelial-derived cells in PDAC extends the list of possible alternate sources and regulation of MUC16. Using publicly available data, we perform molecular characterization of epithelial tumor cells and mesothelium derivatives in PDAC to decouple tumor-stromal regulation of MUC16.

## Methods

### Processing of scRNA data of human PDAC tissues

The scRNA data of 16 human PDAC tissues and PBMCs (accession no: GSE155698) and stromal cells (DAPI-_CD45-_CD31-_EPCAM-) from 4 KPC (Kras-LSL-G12D; Trp53-LSL-R172H; Pdx1-Cre) C57BL6/J mice were individually obtained from NCBI-GEO data repository and subsequently processed using Seurat R package as provided below (21, 22). The scRNA data was processed according to best practices for data processing and clustering as outlined in the Seurat R toolkit documentation (https://satijalab.org/). The scRNA data (GSE155698) of 42692 cells from 16 PDAC tissues were considered for the analysis. The samples belonging to tissue 11 (PDAC_TISSUE_11A & 11_B) were not included in the analysis as they were processed at two different core facilities. Out of 42692 cells, 16635 cells met the quality control thresholds (MT reads < 5% & nFeature_RNA > 200) and were subsequently processed using standard (logarithmic) normalization with scale factor of 10000. The top 2000 highly variable features identified using Variance Stabilizing Transformation (VST) were used for principal component analysis (PCA). The first 35 principal components (capturing 83.6% of the variability) were used to perform cell type-based cluster groupings. The top 30 cluster markers for each cluster were used to annotate the clusters with their cell type using *Enrichr* database (23). We further identified E-cadherin (CDH1) expressing epithelial cells and stratified them based on MUC16-expression (into above and below median expression groups) to identify differentially expressed genes.

### Processing of scRNA data of KPC stromal cells

The RNA-seq processed data of tumor-stromal subtypes (also known as Moffitt data; n = 357) was retrieved from NCBI GEO repository (accession no: GSE71729). Only primary PDAC samples (n = 145) were used for downstream analysis. The prior normalized scRNA data of stromal cells (DAPI-, CD45-, CD31-, EPCAM-) (GSE129455) from 4 KPC (Kras-LSL-G12D; Trp53-LSL-R172H; Pdx1-Cre) C57BL6/J mice were used to perform clustering and lineage trajectory analysis. We constructed principal components using the top 2000 variable features. The nearest neighbor graph was computed using the top 50 principal components and was subsequently clustered. A subset of stromal cells expressing either Mesothelin (Msln) or Muc1 or Muc16 (3565 cells) were used to create three cell groups: a) Muc1-expressing (928 cells); b) Muc16-expressing (148 cells); and c) Msln-expressing (2491 cells) for lineage trajectory analysis (**Supplementary Methods 1**). The top 500 variable features of this subset were identified and were thereafter used for principal component analysis. The UMAP reduction and cell trajectory analysis was performed using the top 20 principal components. The mesothelial cells expressing highly selective markers (Wt1, Muc16, Msln, Upk3b, Upk1b) were selected as root cells for trajectory inference. We used a larger cohort of Upk1b-expressing mesothelial cells (617 cells) to find early gene expression changes associated with the mesothelial-mesenchymal transition. Besides hierarchical clustering, we also used arbitrary correlation measures to identify genes correlating with the loss/downregulation of mesothelial markers.

### Differential expression testing using scRNA data

The median measure cutoffs were employed for gene-expression-based cell group stratification unless mentioned otherwise. The differential testing for subgroup analysis was done using *FindMarkers S*eurat R function.

### RNA-sequence analysis of KPC and KPCM tissues

The RNA sequencing was performed on pancreatic tumors obtained from KPC (KrasG12D/+; Trp53R172H/+; Pdx-1 Cre) and KPCM (KrasG12D/+; Trp53R172H/+; Pdx-1 Cre, Muc16-/-) mice. The sequence reads were aligned to the mouse genome using HISAT2. The gene counts were estimated using feature Counts and were subsequently normalized using DESeq2 R package.

### Analysis of TME- associated mesothelial-mesenchymal plasticity

The bulk RNA-seq data of parental mesothelial cells and FACS sorted eGFP+ mesothelial cells from tumors (CT1BA5 & BMFA3) were obtained from NCBI GEO (accession no: GSE196740). The pre-processing and normalization were performed using DESeq2 R package (https://bioconductor.org/packages/release/bioc/html/DESeq2.html) prior to differential expression analysis.

### scRNA-seq analysis addressing stromal heterogeneity in genetically engineered mice models (GEMMs)

The scRNA data of GEMM models of normal, KPC late, KPfC late, KIC early, and KIC late were obtained from NCBI GEO (GSE125588). The data were log-transformed with a scale factor of 10000 before the downstream analysis. The stromal cells were identified based on PDPN expression. The epithelioid-like cells were identified based on the markers borrowed from pseudotime trajectory findings (Muc1, Clu). The mesothelial cells were identified using markers (Muc16, Cldn15, Upk1b).

### Cell type annotations

The cell type-specific gene expression data of Descartes (descartes.brotmanbaty.org) developmental database (657 cell types from 15 organs) and Human Protein Atlas (www.proteinatlas.org) (51 cell types of 13 tissues) were downloaded from their respective websites (24, 25).

### TCGA-PAAD analyses

The processed TCGA-PAAD RNA-seq (FPKM-UQ normalized) and clinical data was obtained from UCSC Xena data portal (https://xena.ucsc.edu/). The molecular subtype information according to Bailey’s classification scheme was obtained from previously published sources (26, 27). Differential expression analysis across the sample cohorts of different subtypes was performed, with an FDR-corrected p-value threshold of 0.05 for statistical significance. The preprocessed files of mutational, clinical information and copy number variation were obtained from cBioPortal (cbioportal.org). The data regarding splice variants of TP63 were retrieved from UCSC Xena data portal. The information regarding TP63 isoforms was accessed from RefSeq (https://www.ncbi.nlm.nih.gov/refseq/), and Ensembl-UCSC ID conversion was accomplished using the biomart Ensembl resource. Only samples classified as Pancreatic Adenocarcinoma in “cancer type detailed” clinical information (n = 176) were considered for the analysis, and among them, the samples with MUC16 Z-score expression > 1 were designated as “MUC16-overexpressing”.

### Histological subtype analysis

The normalized expression of MUC16 in QCMG-PDAC (n = 96) and the corresponding sample-wise clinical information regarding histological subtypes was acquired from cBioPortal (cbioportal.org) web server.

### Putative transcriptional factor identification

The Signaling Pathways Project (SPP) database and Ensembl websites were used for the identification of transcriptional factor binding sites in MUC16 and TP63 (28, 29). We also accessed JASPER transcriptional binding site profiles using the Enrichr webserver (https://maayanlab.cloud/Enrichr/) for validation.

### Reanalysis/appraisal of RNA-seq data from other sources

We used the author-deposited differential gene expression data available on NCBI GEO (GSE140484) on pancreatic tumors grown using orthotopically implanted TP63-expressing SUIT2 cells in NOD-scid gamma (NSG) mice. Similarly, the log-2-fold differences (log2(µ_sh_/µ_shR_)) in the expression of genes between p53 R172H/null murine PDAC cells treated with doxycycline-inducible control (shR) or anti-p53 shRNA (sh1 & sh2) were estimated using the data deposited in NCBI GEO (GSE114502). The RNA-sequence data of genotypes KRAS Trp53fl/+ and KRAS Trp53fl/+ TAp63fl/fl were obtained from ArrayExpress (accession code: E-MTAB-4415) and were preprocessed and normalized using Kallisto/sleuth and DESeq2 FPKM function. We then computed log-2-fold differences (log2(µ_KPTAp63fl/fl_/µ_KPC_)) in the expression of genes and nominal p-values (t-test).

### CCLE cell line proteomic analysis

The data pertaining to copy number alterations and protein abundance ratios of CCLE-PAAD cell lines (n = 15) were accessed from cBioPortal (cbioportal.org) and DepMap (depmap.org) web portals. The normalized gene and protein abundance data was used to perform differential expression analysis between cell line groupings created using MUC16 protein expression (MUC16-high & MUC16-low).

### HPA cell- type signatures

The gene signatures of individual cell types of Human Protein Atlas data were estimated using a one-sided Student t-test (p < 0.05). We only considered a gene as a gene signature for a particular cell type if the gene was not overexpressed in more than three cell types (24).

### GSEA analysis

GSEA analysis was performed using in-house generated HPA cell type gene signatures (see HPA cell signatures methods section) on MUC16-high and TP63-high cohorts of TCGA-PAAD with 1000 phenotype permutations.

### Descartes developmental gene signature and analysis

Descartes developmental annotations (https://descartes.brotmanbaty.org/) of differentially expressed genes of MUC16-high and TP63-high groups were accessed using *Enrichr* web server (23). A gene was considered as a pancreatic mesothelial cell-specific signature if the fractions of mesothelial cells expressing the gene were significantly higher than other cell types (p < 0.05). We excluded the gene expression fractions in mesothelial cells of other origins for this analysis. In addition, the fractions of cells expressing the putative signature must be higher in pancreatic mesothelial cells relative to all other cells/cell types (22, 30).

### Immunogenicity

The prediction binding affinity using 9-mer peptide sequences of the mutated genes was performed with MHCPan and MHCFlurry across TCGA-PAAD cohort. The sample-wise immunogenic loads were used to perform differential genomic/transcriptomic analysis as well as survival analysis.

### Immunophenotype analysis

In-silico immunophenotyping on prior normalized TCGA and Moffitt PDAC RNA seq datasets was performed using CIBERSORT using standard parameters with LM22 signatures (31). The two-tailed Student t-test was used to evaluate the statistical significance for group-based analysis. The correlation tests were performed using the Pearson approach, and p-value < 0.05 was considered statistically significant.

### Statistical analysis

The hypothesis testing between groups/subgroups was conducted using t-test. The p-values reported in the boxplots were estimated using *stat_means_compare* function of ggplot2 package.

## Results

### TME induces changes to the cell identity of MUC16-expressing mesothelial cells

**Figure 1:**
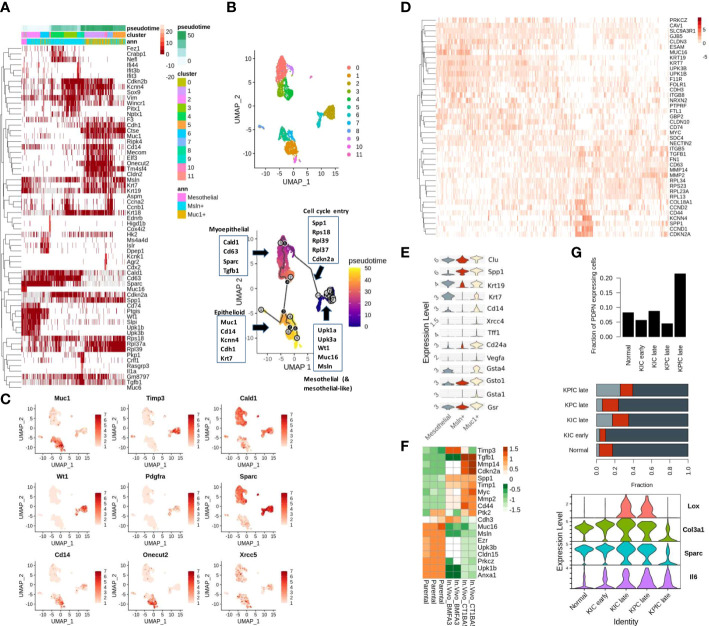
Mesothelial cell plasticity in stromal and tumor compartments: **(A)** The highly variable genes across Msln-expressing, Muc1-expressing stromal, and mesothelial (Meso) cell states are depicted with respect to pseudo-time. The left-most portion of the heatmap includes Muc16-expressing and other mesothelial cells variably expressing mesothelial markers (Wt1, Upk3b, Muc16, Upk1b, Msln). The Muc1-expressing cells clustered at the right-most portion of the plot show co-expression of cytokeratins (Krt7, Krt19, Krt18), epithelial (Muc1, Cdh1, Elf3, Cldn2), and myeloid markers (Spp1, Kcnn4). Muc1-expressing cells are observed later during the pseudo-time trajectory. The mesenchymal cells bridging the path between the Muc16-expressing and Muc1-expressing cells exhibit high expression of mesenchymal markers (Vim, Tgfb1, Cald1, Cd63, Sparc) with variable expression of mesothelial and epithelial cells. Annotation columns on the top represent pseudo time (top), cell clusters (middle), and membership in either of the three stratified cohorts (Meso, MUC1-exp, MSLN-exp) (bottom). **(B)** The clustering and trajectory inference analysis revealed continuity in the transcriptional path among the three predefined cell types: a) MUC16-expressing mesothelial, b) Mesothelin-expressing (Msln), and c) MUC1-expressing KPC stromal cells (n = 3365) **(C)** UMAP representation of PDAC stromal cells expressing select genes specific to distinct cell types, DNA repair, and metaplasia (Cd14, Onecut2, Pdgfra, Muc1, Xrcc5) **(D)** The early transcriptional changes associated with mesothelial-mesenchymal cell state is depicted in the heatmap where the loss of tight-junction and cell-adhesion genes (Cldn3, Gjb3, Prkcz, Cdh3, Cd200, Cldn15, NrxN) correlated with a gain of osteopontin (SPP1) and MMP expression. **(E)** Violin plots of pre-selected markers showing overexpression of oxidative stress/redox signaling (Gsta1, Gsto1, Gsta4, Gsr), Polycomb complex-related genes (Muc1, Clu, Cd24a, Cd14) and DNA damage/senescence (Xrcc4, Vegfa) in Muc1-expressing stromal KPC cells. **(F)** The heatmap depicts the TME-induced downregulation of cell-to-cell adhesion genes in FACS-sorted eGFP mesothelial cells obtained from mice PDAC tumors (BMFA3 and CT1BA5) compared to parental mesothelial cells. **(G)** The higher fraction of PDPN-expressing stromal cells relative to tumor cells was noted in late KPfC models tissues (top) than in normal pancreas and other PDAC mice models; although mesothelial cells fractions were comparable across different PDAC mice tissues (middle); the frequency of Muc1-expressing cells were more prominent in late PDAC tissues (middle); The PDPN-expressing stromal cells of KPC PDAC tissues showed overexpression of activated state (Tnf, Lox, Sparc & IL6) compared to KPfC tissues (bottom).

Mesothelial cells undergo mesenchymal transition before aiding in reparative cancer and tissue injury phenotypes. Recent single-cell studies demonstrate that the mesothelium-derived CAFs, characterized by their expression of both mesothelial as well as fibroblast markers, infiltrate the PDAC tumors to modulate the tumor-stromal dialogue. Similarly, a few other studies also noted that the abundance in the expression of mesothelial markers (Sciellin/SCEL, Mesothelin/MSLN, MUC16) at the invasive front is correlated with poor outcomes in GI cancers (3, 32, 33). We also noted an over-representation of mesothelial signatures in the MUC16-high expressing samples of TCGA-PAAD cohort annotated using Descartes transcriptional signatures of pancreatic cells (**Supplementary Figures 1A, B**; **Supplementary File 1**) (25). To determine the association between MUC16 and mesothelial plasticity, we used scRNA data of fibroblast-enriched stromal cells (DAPI-_CD45-_CD31-_EPCAM-) from four KPC mice and identified a small fraction (2%; 158/8524 cells) of Muc16-expressing mesothelial cells. However, we noted the shared expression of mesothelial markers (Upk1b, Upk3b, Wt1, Msln) in a larger subset of stromal CAFs (**Figure 1A**). The cluster analysis of all stromal cells revealed the presence of distinct CAF subpopulations characterized by either myeloid-like (Cd14-expressing), myoepithelial/smooth muscle (SMC) (Caldesmon/Cald1), and mesothelial (Muc16, Upk3b, Upk1b) transcriptional activity. The majority (~75%; 6393/8524 cells) of the CAFs expressed myopeithelial/SMC marker Cald1, and around 40% (3370/8524 cells) of them expressed Mesothelin (Msln). Although Msln-expressing cells displayed a high level of heterogeneity without any clear commitment towards epithelioid-like, mesothelial, or SMC fractions, a subset (~23%; 800/3370 cells) of these cells showed distinctive transcriptional profile characterized by their expression of cytokeratins (Krt7, Krt19), epithelial membrane antigen (Muc1) and myeloid markers (Cd14, Kcnn4) (**Figure 1A**).

From here on, we will use the term “epithelioid-like” to refer to these CAFs due to their co-expression of both epithelial and myeloid markers. Therefore, to examine if shared Msln expression indicates their co-evolution or shared lineage, we constructed cell trajectories with only mesothelial (Muc16-expressing), Msln-expressing and Muc1-expressing cells (3565 cells). The clustering and trajectory analyses yielded three distinct cell clusters with a continuous trajectory path (**Figure 1B**). The transition with respect to pseudo-time also showed a gradual loss of mesothelial markers (Muc16, Upk3b, Upk1b) coinciding with the gain in the expression of ribosomal subunits (Rps27a, Rpl23a, Rpl17), cell cycle re-entry and osteopontin signaling (Spp1, Cd44, Cdkn2a, Ccnd1) (rho < -0.4 & p < 0.0001) (**Figures 1A, C**). Moreover, the overexpression of aging/DNA repair genes (Xrcc4, Vegfa, Clu), galectins (Lgals2, Lgals3), and redox/glutathione genes (Gsta1, Gsta4, Gsr, Gsto1) in Muc1-expressing CAFs signify senescent pathways behind their emergence (**Figure 1E**). We also independently compared Muc16-expressing mesothelial CAFs to a larger fraction of mesothelial CAFs that are marked by Upk1b expression (~5%; 459/8525) where we found higher expression of cytoskeletal, cell-cell adhesion, and junction genes (Cldn3, Gjb3, Prkcz, Cdh3, Cd200, Cldn15, Nrxn2) in Muc16-expressing mesothelial cells. In contrast, the Upk1b-expressing mesothelial cells exhibited increased expression of genes involved in extracellular matrix degradation (Mmp2, Mmp9, Mmp14), MYC-regulated ribosomal subunits (Rpl13, Rpl23, Rpl34), cell-cycle/proliferation (Cdkn2a, Ccnd2, Cd44, Ccnd1), mesenchymal adhesion (Itgb5, Tgfb1, Fn1), osteopontin (Spp1) and HLA-class II (Cd74) (**Figure 1D**).

Due to low pure fractions of MUC16-expressing mesothelial cells in PDAC tissues when compared to the normal pancreas, we hypothesized that Tumor Microenvironment (TME) influences MUC16 expression and mesothelial cell identity. The FACS sorted eGFP mesothelial cells from mice PDAC tumors (BMFA3 and CT1BA5) showed decreased cell-cell adhesion (Muc16, Cldn15, Cdh3) and ERM protein Ezrin (Ezr) with concomitant overexpression of mesenchymal (Acta2, Tgfb1), proteases (Mmp2, Mmp14, Timp1, Timp3) and cell cycle (Myc, Cdkn2a) genes compared to parental mesothelial cells (**Figure 1F**). We further used scRNA data of normal pancreas (60 days old), early (40 days old), and late (60 days old except for KPC where KPC mice were 6 months old at sacrifice) tumor tissues from multiple mouse PDAC models (*KPC (Kras^LSL−G12D/+^Tr*p53^LSL−R172H/+^ *Ptf1a^Cre/+^), KIC (Kras^LSL−G12D/+^Ink4a^fl/fl^Ptf1a^Cre/+^) and KPfC (Kras^LSL−G12D/+^Trp53^fl/fl^Pdx1^Cre/+^)*) to quantify the fraction of infiltrating mesothelial cells. We saw Pdpn-expressing stromal cells were substantially higher in *KPfC* late tissues (21%) compared to normal and PDAC tissues from other mice models (4-9%). Although Muc16-expressing mesothelial cell fractions were low in all mice tissues, their presence was noted in late KPfC (~4%) and normal tissues (~7%) but not in *KPC* (late) tissues. In contrast, the infiltration of epithelioid-like cells was more evident in all late PDAC tissues, with higher prominence in late KPfC tissues. The stromal cells of KPC and KIC tissues were found to be in an “activated” state relative to KPfC PDAC tissues due to their overexpression of markers lysyl-oxidase (Lox), Col3a1, Sparc, and Il6 (**Figure 1G**).

### The loss of MUC16 impacts fibroblast polarization by causing dysregulation in myofibril organization

**Figure 2:**
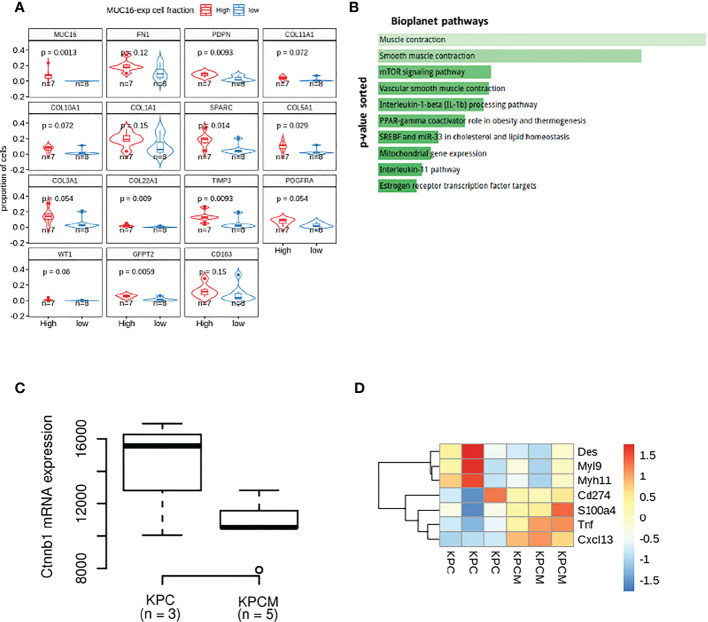
The transcriptional differences in the MUC16 knockout (KPCM mice) tumors compared to KPC PDAC tissues indicate changes to tumor-stromal environment: **(A)** The boxplots depict the proportion of cells expressing SMC/fibroblast markers in samples with high and low frequency of MUC16-expressing cells. **(B)** The enrichment analysis of downregulated genes in KPCM tissues compared to KPC tumors show underrepresentation of pathways of SMC maintenance and contraction. **(C)** The downregulation of beta-catenin transcription in KPCM tumors is evident relative to KPC tissues. **(D)** The heatmap illustrates the genes markers of iCAFs (Tnf, S100a4, Cxcl13), myCAFs (Myh11, Myl9, Des), and immune checkpoint (Cd274) across KPC (n = 3) and KPCM (n = 3) mice is shown; The consistent downregulation of myCAF markers in KPCM tissues indicate the paracrine modulation of CAF polarization by MUC16.

Our previous efforts uncovered MUC16 regulation of endothelial cell binding, lymphangiogenesis (NRP2, VEGFC), and cell-cell adhesion integrity (34). In parallel, the scRNA data from 15 PDAC tissues samples also showed higher infiltration of cells expressing SMC/fibroblast markers (PDPN, COL5A1, COL22A1, TIMP3, SPARC, WT1, GFPT2) in samples with higher proportions/fractions of MUC16-expressing tumor (epithelial) cells (n = 7) (**Figure 2A**). Our accompanying paper on KPCM experimental models (KrasG12D/+; Trp53R172H/+; Pdx-1-Cre; Muc16-/-) shows that knockout of Muc16 in tumor cells substantially decreases α-SMA (Acta2) levels in CAFs. Moreover, the PDAC tissues of KPCM mice also showed decreased infiltration of α-SMA+ cells, indicating that intratumoral loss of Muc16 can alter stromal cell function (34). As Muc16 loss caused a considerable decrease in myofibroblast (α-SMA+) infiltration, we investigated if Muc16 loss is associated with global changes to fibroblast polarization. The transcriptome profile of KPCM tumor tissues (n = 3; 25-week-old) did not demonstrate changes to panCAF marker Podoplanin (Pdpn) but showed significant downregulation of genes involved in cytoskeletal, smooth muscle contraction, myofibril organization and focal adhesion (p < 0.05) relative to age-matched KPC (n = 3; 25-week old) PDAC samples (**Figure 2B**). Specifically, the KPCM tissues showed decreased transcription of beta-catenin (Ctnnb1) and increased expression of iCAF markers (Tnf, S100a4, Cxcl13) and immune checkpoint (Cd274) (p > 0.05). On the contrary, KPC tissues showed overexpression of myofibroblastic (myCAF) markers (Myh11, Myl9, Des; p < 0.05). The dysregulation of several actomyosin filaments that are classical markers of myCAFs indicates that Muc16 has an extratumoral role in maintaining myofibril organization in myCAFs. (**Figures 2C, D**).

### The interplay between TP53 and TP63 regulates MUC16 as well as mucinous molecular features

**Figure 3:**
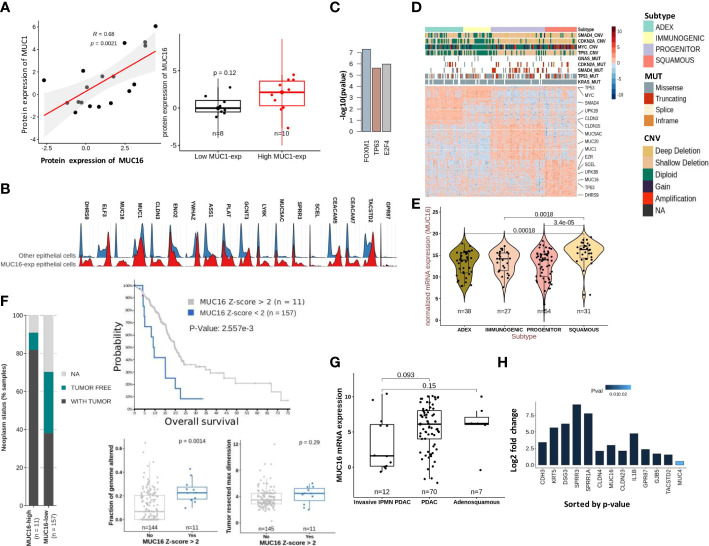
Molecular characteristics of MUC16 expressing tumors: **(A)** A strong correlation between MUC1 and MUC16 protein expression was found in CCLE PDAC cell lines (n = 18) (left); The boxplot represents the significant differences in MUC16 expression between MUC1-high (n = 10) and MUC1-low (n = 8) expressing cell lines. **(B)** The ridge plot of genes overexpressed in MUC16-expressing single cells relative to all other epithelial (E-cadherin expressing) cells across 15 PDAC patients. **(C)** The putative transcriptional factors identified by ENCODE/CHEA that putatively regulate the overexpressed genes in high MUC16-expressing PDAC tumors (TCGA-PAAD; Z-score > 1; n = 22). **(D)** Heatmap of differentially expressed genes across 4 molecular subtypes of TCGA-PAAD cohort (n = 149) following Bailey’s classification; the annotation bars on the top illustrate the different subtypes, mutation, and copy number status of hallmark genes (SMAD4, MYC, CDKN2A, KRAS, TP53) in their respective samples (n = 149). **(E)** The significant upregulation of MUC16 in squamous subtype tumors compared to other tumor subtypes (p < 0.05) is illustrated. **(F)** The correlation between MUC16 expression and clinico-pathological variables: a) survival (top), b) fraction of genome altered (bottom-left), c) tumor resected maximum dimension (bottom-right), and d) neoplasm status is depicted. **(G)** The boxplots show overexpression of MUC16 in histologically classified PDAC and adenosquamous subtypes compared to IPMN-derived PDAC samples (n = 93; p< 0.05). **(H)** The log fold change differences (Log2FC) of expression of MUC16, squamous markers (SPRR1A, SPRR3A, TACSTD2, GPR87, KRT5), cell-junction genes (CLDN4, CLDN23, GJB5) between TP63 OE SUIT2 cell lines relative to their control are graphically summarized.

The cytoplasmic tail of MUC16 maintains cell-cell adhesion integrity due to its potential interaction with ezrin/radixin/moesin (ERM) proteins that crosslink the actin cytoskeleton (4). Several proteins that maintain actin assembly, cell-cell adhesion, and focal adhesion are under the direct regulation of TP53 family members, TP53 and TP63 (23, 30). The tumor subtyping studies associate MUC16 expression withbasal/squamous subtypes that are predisposed to higher stromal involvement (31). Therefore, we used the scRNA data from 15 human PDAC tissues (16635 cells) for a focused evaluation of MUC16. Across these samples, MUC16 expression was merely limited to two epithelial tumor cell clusters, whereas expression of other mucins (like MUC1) was more uniformly distributed across the epithelial and SMC/myoepithelial cell clusters (**Supplementary Figures 2A, B**). The co-expression of MUC1 was noted in at least 70% of all cells that expressed mucins (MUC4, 5AC, 20, and 16). In part, these findings were also reproducible in PDAC cell lines, and specifically, strong correlations were found between MUC16 and MUC1 at a protein level (R = 0.68; p = 0.002) (**Figure 3A**).

The scRNA-seq analysis also revealed a distinct transcriptional profile of MUC16-expressing cells relative to all other epithelial (E-cadherin expressing) cells due to their higher reliance on glycolytic (HK2, ENO2, ELF3, CLDN3) and unfolded protein response (YWHAZ, ASS1) pathways (**Figure 3B**; **Supplementary Figure 2A**). Although MUC16-expressing cells also expressed other classical mucins (like MUC20, MUC4 & MUC1), the co-expression of genes involved in TP63 regulated processes like cornification (PLAT, SPPR1A, SPRR3, SCEL), tight-junctions (CLDN3) and anoikis resistance (CEACAM5, CEACAM7, TACSTD2, GPR87) were discriminative (**Figure 3B**; **Supplementary Figure 2C**). Consistent with scRNAseq analysis, the MUC16 overexpressing samples (n = 22; Z-score > 1) of the TCGA-PAAD cohort (n = 176) showed overexpression of genes regulated by TP63 (ENCODE/CHEA; p < 0.05) (**Figure 3C**; **Supplementary File 1**). Furthermore, the subtype analysis using Bailey’s schema showed MUC16 upregulation in samples classified as a squamous molecular subtype (26, 35). Unlike other tumor subtypes, squamous tumors scored poor on several prognostic indices (tumor weight, fraction of genomic altered, histological grade) and displayed an increased frequency of genetic alterations in hallmark genes (SMAD4, MYC, KRAS, and TP53) (**Figures 3D, E**; **Supplementary File 2**). Similarly, MUC16-overexpressing TCGA-PAAD tumors also registered poor on a few clinico-pathological scales (fraction of genome altered, neoplasm status, tumor resected maximum dimension, and survival) (**Figure 3F**).

The analysis using histological subtypes of QCMG-PAAD cohort (n = 96) pointed to significant overexpression of MUC16 in *de novo* adenocarcinomas (n = 70) and adenosquamous (n = 7) cancers compared to PDAC-originated from Intraductal Papillary Mucinous Neoplasms (IPMNs) (n = 12) (**Figure 3G**). Due to its large size, the complexity associated with the transcriptional and post-transcriptional regulation of MUC16 is overburdening. However, recent efforts show evidence of a TNF-responsive NFkb binding site in the promotor region of the MUC16. Besides that, we show the presence of CTCF and TP63 binding sites near the transcriptional start site (TSS) and promoter region of MUC16, respectively (**Supplementary File 3**). The TP63 gene itself contains a CTCF binding site in its promoter.

To further examine the associations between MUC16 and TP63, we profiled the pancreatic tumors grown using orthotopically implanted TP63-expressing SUIT2 cells in NOD-scid gamma (NSG) mice and saw significant overexpression of MUC16 and other basal subtype markers (CLDN4, SPRR1A, SPRR1B, SPRR3, KRT5, DSG3) and cell-cell adhesion proteins (DSG3, PKP1, NECTIN1, CLDN4, CDH3, CLDN1) (**Figure 3H**).

**Figure 4:**
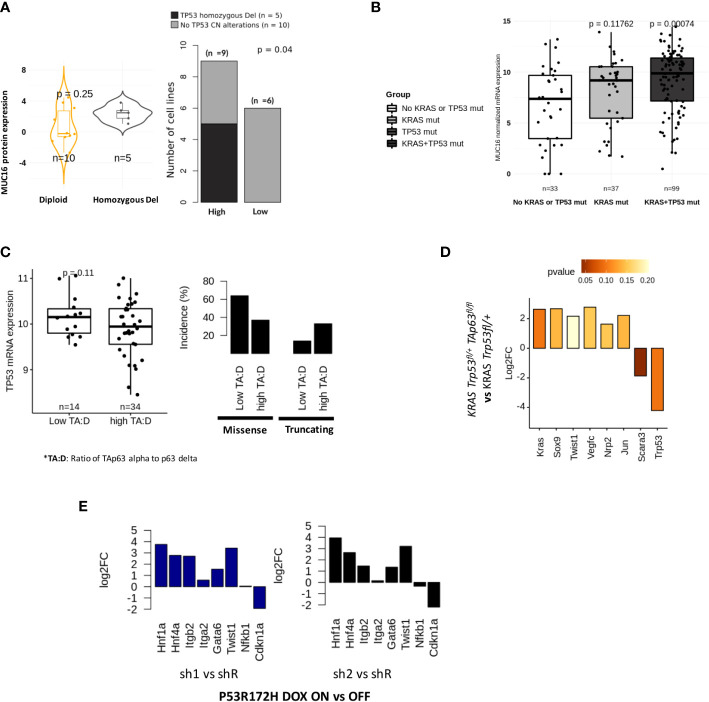
Tp53 and Tp63 synchronously regulate MUC16, SOX9, and KRAS signaling: **(A)** MUC16 protein expression for TP53 copy number alterations across 15 CCLE PDAC cell lines (TP53 non-mutant) (left). The incidence of TP53 alterations in cell lines is exhibited with respect to (n = 9) and low (n = 6) MUC16 protein expression (right). **(B)** MUC16 overexpression in KRAS and TP53 mutant PDAC samples is shown using boxplots (TCGA-PAAD) (left); The expression of MUC16 is significantly different across different types of TP53 mutations except for WT (right) **(C)** The ratio of TAp63 to Tp63 delta correlates with TP53 mRNA expression (left); The retention of TP53 truncating and TP53 missense mutations are noted in samples with high TAp63 to Tp63 delta expression ratio and low TAp63 to Tp63 delta respectively (right). **(D)** The TAp63 knockout murine cell lines (TAp63 fl/fl, TP53+/fl KRAS) show downregulation of KRAS, SOX9, claudins (Cldn4, Cldn3, Cldn6, Cldn7) and collagens (Col4a3, Col4a4, Col8a1) with concomittant upregulation of TP53- apoptotic signaling (Tp53, Unc5b, Scara3). **(E)** p53 knockdown using RNA interference (shRNA) in KRAS-R172H/null murine PDAC cells shows upregulation of integrin signaling (ITGB2, ITGA2), EMT (Twist1) and progenitor subtype associated transcriptional factors (HNF1A, GATA6, HNF4A) relative to parental cells; the log2 fold changes are represented in the barplots.

We evaluated if TP53 functional status may impact MUC16 expression. In the CCLE-PAAD dataset, we found homozygous deletion to be significantly more frequent (Fisher exact p-val = 0.04) in MUC16-high (55%; n = 9) than in MUC16-low (0%; n = 6) (**Figure 4A**). Although these align with our previous findings of cell cycle progression and failed TP53-mediated CDK control in MUC16-expressing cells, it is unclear why MUC16 is overexpressed in TP53 mutants of TCGA-PAAD cohort, including gain-of-function mutants (**Figure 4B**). On the same lines, we also previously showed that overexpressing MUC16-cter itself downregulates TP53 allowing cell survival *via* JAK2/STAT3/GR axis (36). TP63 and TP53 have shared control of ~1000 common genomic binding sites in genes involved in cell cycle, apoptosis, and DNA damage repair pathways. Besides that, TP53 also directly controls the transcriptional splicing of TP63 isoforms, where TP53 gain-in-function mutations are shown to downregulate the longest isoform of TP63, isoform 1 (also known as TAp63alpha). Firstly, we saw strong correlations between TP63 and TP53 expression across the TCGA-PAAD cohort (rho = 0.27; p < 0.05), however, the samples with a high expression ratio of TAp63alpha to p63-delta were only limited to a subset of samples (n = 34) that have a higher incidence of truncating mutations and lower expression of TP53 (**Figure 4C**). It is believed that TAp63 takes control and modulates some of TP53 functions, such as apoptosis and desmosome maintenance, especially in case of deficiency of the latter.

Although we found a TP63 binding site in the promotor region of MUC16, we postulated that the sustenance of RAS-mediated signaling might be required for overall mucinous transformation, like we previously demonstrated (37, 38). To evaluate if KRAS signaling requires functional TP63 in TP53 deficient tumors, we used murine PDAC cell lines of *KRAS Trp53^fl/+^* and *KRAS Trp53^fl/+^ TAp63^fl/fl^* genotypes and found decreased expression of transcriptional factors (Kras, Sox9, Twist1, Jun) and lymph angiogenesis/perineural invasion (Nrp2, Vegfc) in *KRAS Trp53^fl/+^ TAp63^fl/fl^* cell lines (p > 0.05). Interestingly, as we also noted higher expression of TP53 expression in *Trp53^fl/+^ TAp63^fl/fl^* cell lines, we suppose that the lack of TAp63 alpha isoform may revert to oxidative stress-induced apoptotic signaling by TP53 (Scara3, Unc5b, Trp53) (p < 0.05) (**Figure 4D**).

### Loss of TP53 amplifies hedgehog, angiogenesis, and mucinous phenotype

As shown in the earlier section, we found increased stromal mediation in KPfC models relative to KPC models. We also observed that ~50% of samples in the TCGA-PDAC cohort have either TP53 truncating mutations, heterozygous deletion, or both. The frequency of such TP53 loss-in-function events is more evident in mucinous molecular subtypes: squamous (27%) and progenitor (44%) (**Figure 3D**; **Supplementary File 2**). It is important to note that the TP53 mutations act as dominant negative functions; therefore, we expect that such mutations may override associated WT. We noticed the upregulation of hedgehog (IHH, SHH), angiogenesis (VEGFA, F11R, FOSL1, PDLIM3, EYA1, SFRP1, TJP1, RAC1), glycolytic (ENO2, ELF3, HK2), SMAD signaling (SMAD3, SMAD2), NFkB (RELA) and goblet cell transcriptional signaling (SPDEF) in samples with loss-of-function alterations in TP53 (**Supplementary Figure 3A**). We hypothesize that the heterozygous loss of TP53 is not just limited to TP53 but involves copy number changes at a chromosomal locus level. These findings indicate that the paracrine angiogenic signaling conditioned by hypoxia may predispose these tumors to increased infiltration of mesothelium-derived fibroblasts, as evident in KPfC mice models (**Figure 1G**). We further suspected that loss-of-function of TP53 promotes progenitor phenotype as opposed to a squamous phenotype that is characteristic of TP53-gain-in-function mutants. Using p53 R172H/null murine PDAC cells either treated with doxycycline-inducible control (shR) or anti-p53 shRNA (sh1 & sh2), we show upregulation of integrin signaling (Itgb2, Itga2), EMT (TWIST1) and progenitor transcriptional factors (Hnf1a, Hnf4a, Gata6) in anti-p53 shRNA (sh1 & sh2) cell lines (**Figure 4E**). On the other hand, we also show correlations between *DNp63delta* and TP53 missense mutations where progressive increases in TP53 alterations were found in the upper quartiles of its expression in TCGA-PAAD samples (**Figure 4C**).

### MUC16 and immunogenicity

**Figure 5:**
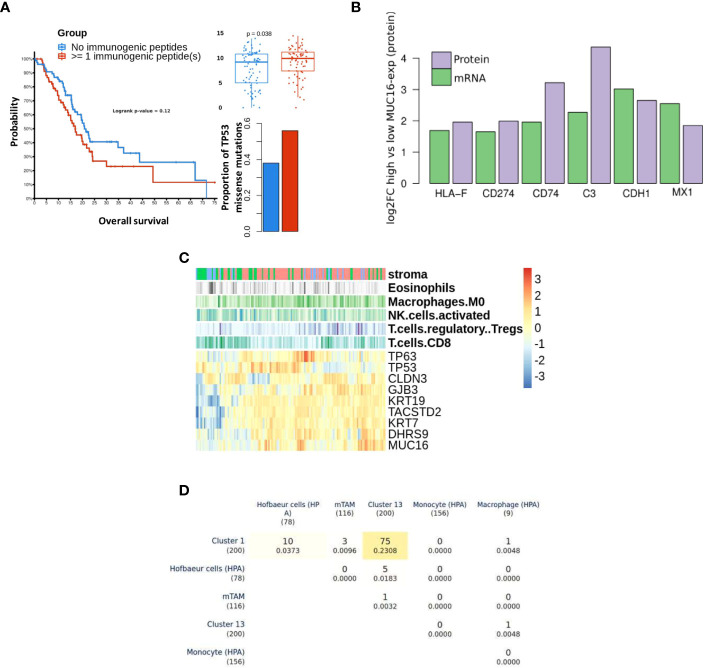
Differential immune profile of MUC16 expressing tumors: **(A)** The survival probability (left) between samples with pre-estimated immunogenic loads is shown. The samples with detected immunogenic loads showed poorer survival (p > 0.05), higher proportions of TP53 missense mutations (p < 0.05), and high MUC16 expression (p < 0.05). **(B)** The immunomodulatory genes are upregulated at both mRNA and protein level in MUC16-high expressing PDAC CCLE cell lines (n = 9) compared to MUC16-low expressing cell lines (n = 6) (p < 0.05); the log2 fold changes are represented with green and purple bars for mRNA and protein levels respectively **(C)** The gene expression heatmap of selected markers in Moffitt PDAC cohort is illustrated. The top three annotation columns depict the level correlation estimates (spearman method) between MUC16 and immune cell fractions (macrophages, NK cells, Tregs, and CD8 T cells). **(D)** Triangular matrix representing overlap (& Jaccard index similarity coefficient) between markers of PDAC macrophage cell clusters (cluster 1 & 13), murine Tumor-Associated Macrophages (mTAMs), circulating monocyte, circulating macrophage, and Hofbauer (embryonic/developmental) cells.

MUC16 showed significant co-expression with antigens (tumor-antigens & autoantigens) and acute phase reactants such as transglutaminases (TGM2), CEACAMs, C-Reactive Protein (CRP), and alkaline phosphatase (ALPP) (**Supplementary File 1**). We performed prediction binding affinity of 9-mer peptides of genes with identified mutations across TCGA-PAAD samples using *MHCPan* and *MHCFlurry* and estimated sample-wise putative immunogenic loads. The samples with at least 1 identified immunogenic peptide (n = 70) showed poorer survival probability (p = n.s), a higher proportion of TP53 mutations (one-sided fisher-exact p < 0.05), and overexpression of MUC16 (p < 0.05) (**Figure 5A**). Moreover, the PDAC cell lines with high MUC16 protein expression in the CCLE database also exhibited significant upregulation of immunomodulatory proteins (CD274, HLA-F, CD74, MX1, CDH1, and C3) at both mRNA and protein levels (p< 0.05) (**Figure 5B**).

Using bulk RNA seq data, we observed overexpression of cornification markers (SCEL, SPRR3, PPL, SPRR1B), MSLN, MUC1, and MUC16 in samples with marked stromal cell infiltration (activated stroma). Also, the immunophenotype of these samples exhibited significant correlations between MUC16 mRNA expression and putative fractions of immune cell subsets, particularly macrophages (r = 0.26, p < 0.05) and T-regulatory cells (r = 0.21, p <0.05). Conversely, MUC16 expression negatively correlated with cell fractions of CD8 T cells (r = -0.18, p< 0.05) and NK cells (r = -0.21, p < 0.05) (**Figure 5C**). As MUC16-expressing tumors showed significantly higher infiltration of macrophages expressing mesothelial-specific markers (GFPT2, PDPN, WT1) similar to fibroblasts, we attempted to identify if these subsets are likely to share mesothelium-driven developmental pathways. When compared with HPA immune cell signatures, the PDAC macrophages exhibited closer transcriptional similarity to developmental (Hofbauer villous) macrophages than circulating myeloid-derived cell signatures due to their overexpression of CD36, DAB2, HBEGF, and SPP1 (**Figure 5D**) (34).

## Discussion

PDAC progression is triggered by a cascade of events involving stromal cell mediation, autoantigenicity, and desmoplasia (39). Recently, the insidious role of mesothelial plasticity was recognized as a principal modulator of disease progression by shaping the tumor-stromal environment (10). Our work captures the metaplastic changes driven by mesothelial-derived cells in the PDAC tumors and stroma. The lineage trajectories constructed by KPC stromal cells exhibited continuity in the transcriptional path among Muc16-expressing mesothelial cells, mesenchymal/myoepithelial cells (Cald1 and/or Msln-expressing), and Muc1-expressing epithelioid cells. The expression of metalloproteinases (Mmp2, Mmp14) and cell cycle markers (Ccnd1, Ccnd2, Cdkn2a), osteopontin (Spp1), and MYC-regulated ribosomal stress genes (Rps23, Rpl13, Rpl23a, Rpl34) in mesothelial cells negatively correlated with MUC16 expression in mesothelial cells. Moreover, the expression of mesenchymal markers like TGFB1 in a subset of mesothelial cells with low expression of MUC16 indicates that cell-cycle re-entry and ECM degradation may likely precede mesenchymal differentiation. MUC16-expressing mesothelial cells were found in low numbers in human and moused PDAC tissues, and herein we show that this is due to increased TME-mediated MMP activation during MMT. The downregulation of MUC16 in mesothelial-derivatives with MT1-MMPs expression may also concur with increased secretion of MUC16-like, as demonstrated elsewhere (9). The emergence of distinct Muc1-expressing stromal cells of KPC tissues is possibly due to cellular senescence mediated by polycomb complex-related (Onecut2, Muc1, Cd14, Clu), DNA repair (Xrcc4, Clu) and oxidative stress (Gsto1, Gsr, Gsta1, Gsta4) (23, 40–42).

**Figure 6:**
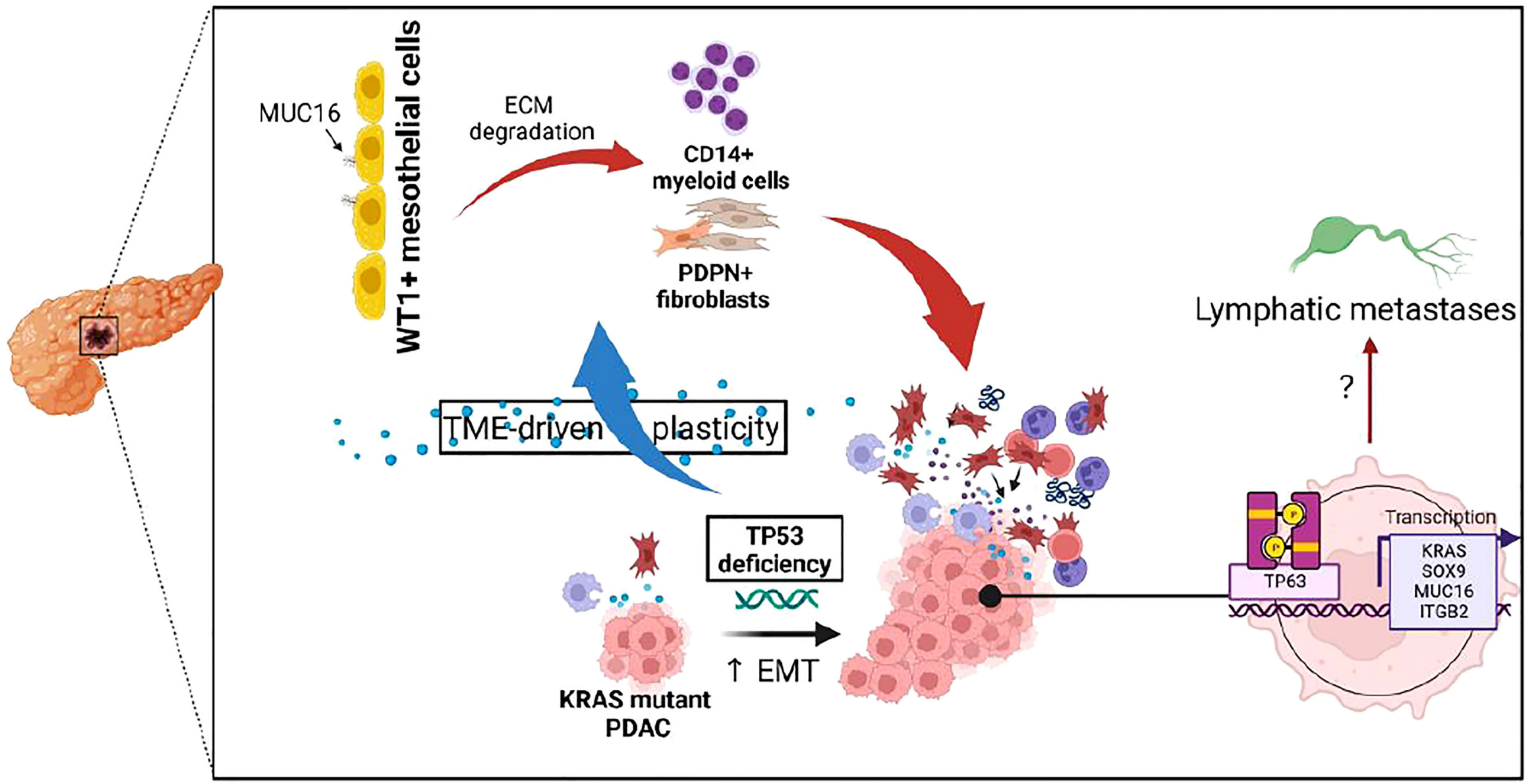
Illustration depicting the impact of TP53 genetic alterations on tumor microenvironment and TP63-mediated tumor cell reprogramming.

The MUC16-expressing epithelial cells displayed a distinct transcriptional profile relative to other mucin-expressing cells due to their overexpression of cancer antigens (CEACAM5, CEACAM7), cornification/squamous (SPRR3, SPRR1A, TACSTD2), and cell-cell adhesion (PARD6B, CLDN4, MYH14, CLDN7, DSG2, LAMC2). Due to the co-expression of squamous markers in MUC16-expressing tumors, we dissected the associations between TP63, TP53, and MUC16, where we identified that TAp63 also mediates SOX9 and KRAS signaling like TP53-gain-in-function mutants (**Figure 6**). It is likely that TAp63 and DNTp63 synchronously co-regulate basal cell expansion, angiogenesis, cellular senescence, and squamous transdifferentiation. We already demonstrated previously that MUC16 facilitates the expression of TP63 effectors involved in angiogenesis (NRP2, SDC1, and PTHLH), and therefore MUC16 may play a vital role in the convergence of TP53 and TP63 pathways (34).

Further, the high infiltration of stromal cells, particularly myofibroblasts expressing TIMP3 and PDGFR, may create fibers and extracellular matrix in MUC16-expressing tumors. We assume that these stromal cells are mesothelial-derived due to the retention of their mesothelial-specific markers, WT1 and PDPN. The gene expression profile of KPCM tissues relative to KPC showed considerable downregulation of actinomyosin intermediate filaments required for myofibroblast differentiation and intercellular junction integrity, supporting its more comprehensive (extratumoral) role in PDAC. On the other hand, the increased influx of macrophages in MUC16-high tumors is possibly due to TP53 dysfunction like demonstrated elsewhere rather than MUC16 over-expression by itself (43).

## Conclusion and future directions

The MUC16-expressing PDAC tumors showed high antigenic loads, differential polarization of mesothelium-derived CAFs, and increased predisposition to TP53 loss. These characteristics likely cumulate and reflect the prognostic burden associated with MUC16 expression in PDAC. Moreover, the genomic and transcriptional dysregulation of TP53 family members (TP53 and TP63) may exhibit a broader impact on mucinous signaling due to their modulation of KRAS and SOX9 activity. This signaling modulation was also accompanied by shifts in the stromal cell proportions and is likely a consequence of altered biological behaviors and the growth of cancer cells. On the other hand, the tumor tissues of the whole-body knockout model of MUC16 showed downregulation of cytoskeletal, smooth muscle contraction, myofibril organization, and focal adhesion pathways besides profound transcriptional changes to fibroblast subtype markers. We expect future studies to address if the decreased myofibroblast polarization in MUC16 deficient tumors results from reshaped tumor-stromal dialogue and impaired MMT process.

## Data availability statement

The datasets presented in this study can be found in online repositories. The names of the repository/repositories and accession number(s) can be found below: https://www.ncbi.nlm.nih.gov/, GSE212777.

## Ethics statement

The mouse studies conducted followed the US Public Health ‘Service’ Guidelines for the Care and Use of Laboratory ‘Animals’ under an approved protocol by the Institutional Animal Care and Use Committee, University of Nebraska Medical Center.

## Author contributions

RC-V performed data acquisition, research, and manuscript preparation. VD performed data analysis and developed immunogenicity in-silico pipelines. RN performed research and figure modifications. ZA independently reanalyzed the data, validated pipelines, and developed final reports. All authors contributed to the article and approved the submitted version.
