# Supplementary material for: MUC16 and TP53 family co-regulate tumor-stromal heterogeneity in pancreatic adenocarcinoma
Source: Front Oncol. 2023 Feb 3;13:1073820. doi: 10.3389/fonc.2023.1073820 (PMC9936860; doi:10.3389/fonc.2023.1073820)
Supplement: Supplementary file 1 [file Image_1.pdf]

Supplementary figure 1

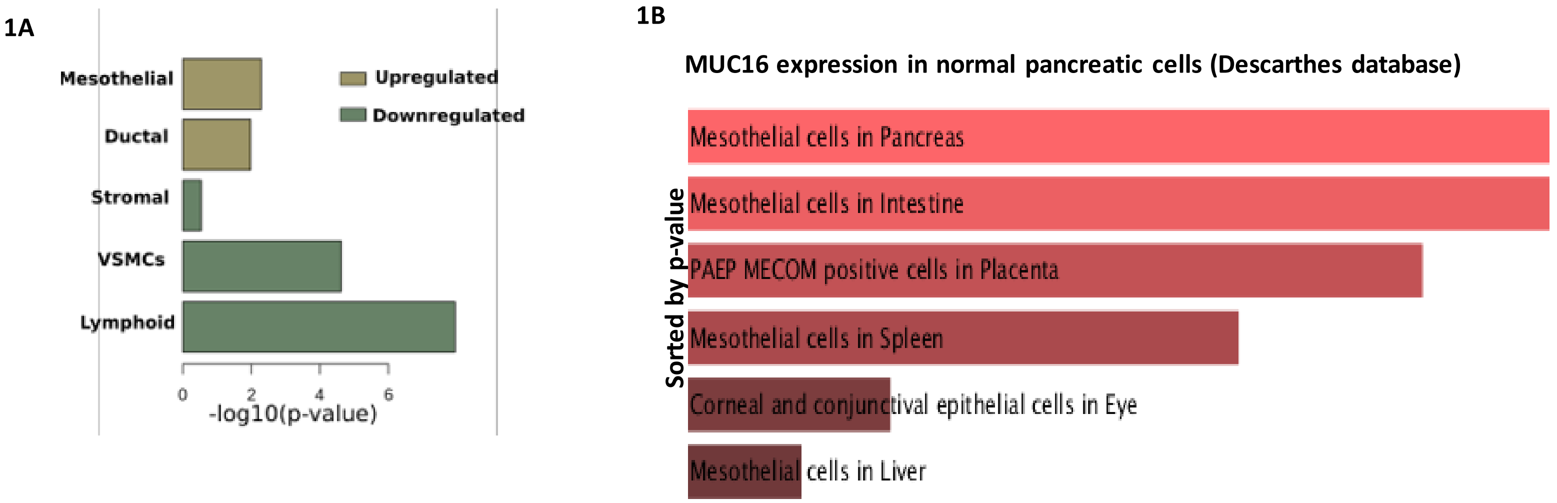

Supplementary figure 1A: The enrichment of pancreatic cell specific transcriptional signatures (Descartes) differentially expressed ($p < 0.05$) in MUC16-high expressing tumors (n = 22) of TCGA-PDAC cohort (n = 176) .

Supplementary figure 1B: The overrepresentation of MUC16 in mesothelial cells of Pancreas and Intestines in noted using Descarthes developmental signature database.
