# Supplementary material for: MUC16 and TP53 family co-regulate tumor-stromal heterogeneity in pancreatic adenocarcinoma
Source: Front Oncol. 2023 Feb 3;13:1073820. doi: 10.3389/fonc.2023.1073820 (PMC9936860; doi:10.3389/fonc.2023.1073820)
Supplement: Supplementary file 2 [file Image_2.pdf]

Supplementary figure 2

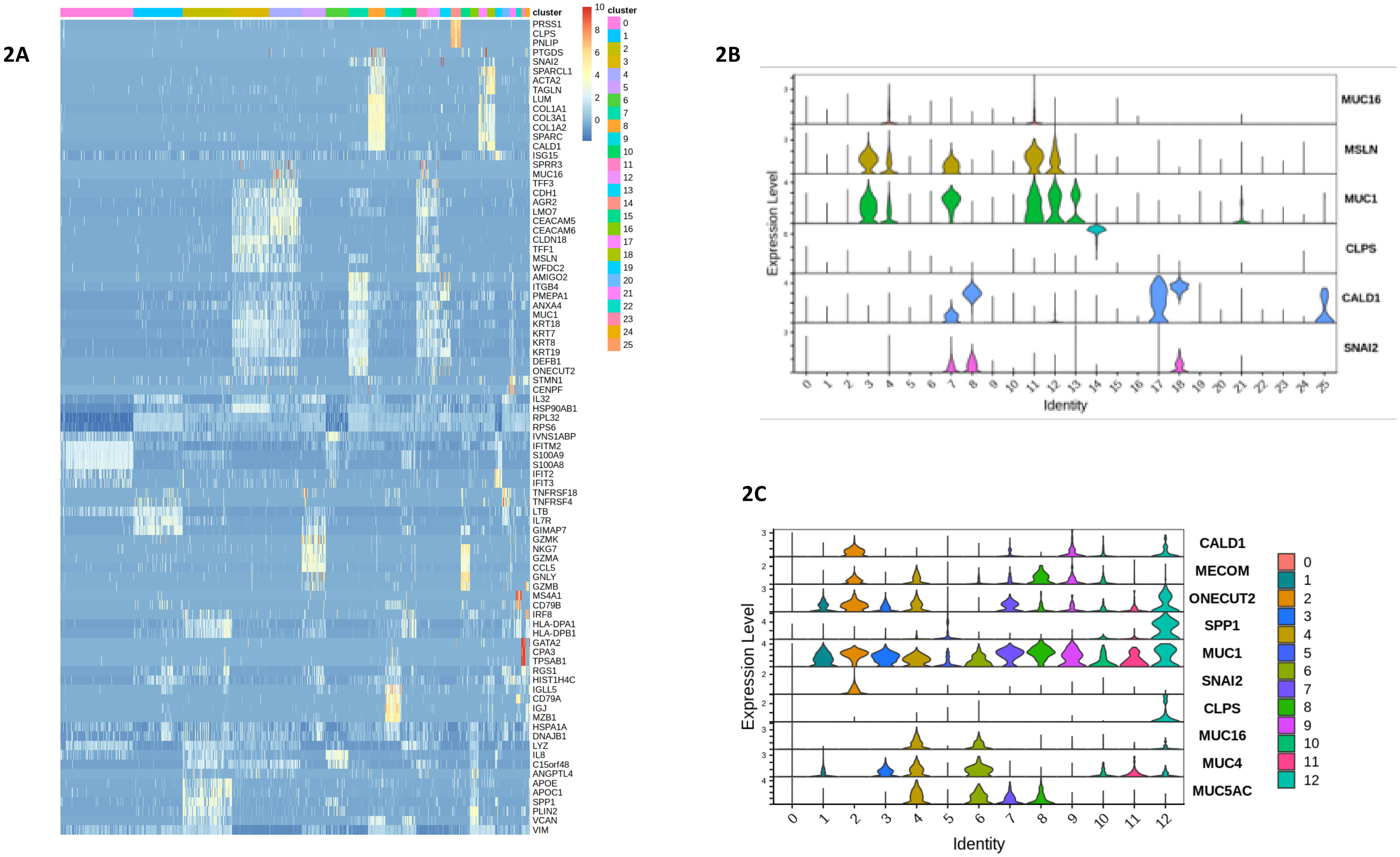

Supplementary figure 2A: The top 10 differentially expressed genes across the clusters are illustrated in the heatmap (left).

Supplementary figure 2B: The cluster markers across the 25 cell clusters obtained from the clustering analysis of 14698 cells from 16 PDAC tissues (left). The distribution of cells expressing select markers (MUC16, MUC1, CALD1, CLPS, SNAI2, MSLN) across the clusters is represented (right).

Supplementary figure 2C: The reclustering of PDAC myoepithelial and epithelial cells showed the considerable presence of cells expressing metaplastic marker (ONECUT2) and Osteopontin (SPP1). Also, high-level view of the cell clusters show co-expression of other mucins with MUC16-expressing cell clusters.
