# Supplementary material for: MUC16 and TP53 family co-regulate tumor-stromal heterogeneity in pancreatic adenocarcinoma
Source: Front Oncol. 2023 Feb 3;13:1073820. doi: 10.3389/fonc.2023.1073820 (PMC9936860; doi:10.3389/fonc.2023.1073820)
Supplement: Supplementary file 3 [file Image_3.pdf]

Supplementary figure 3

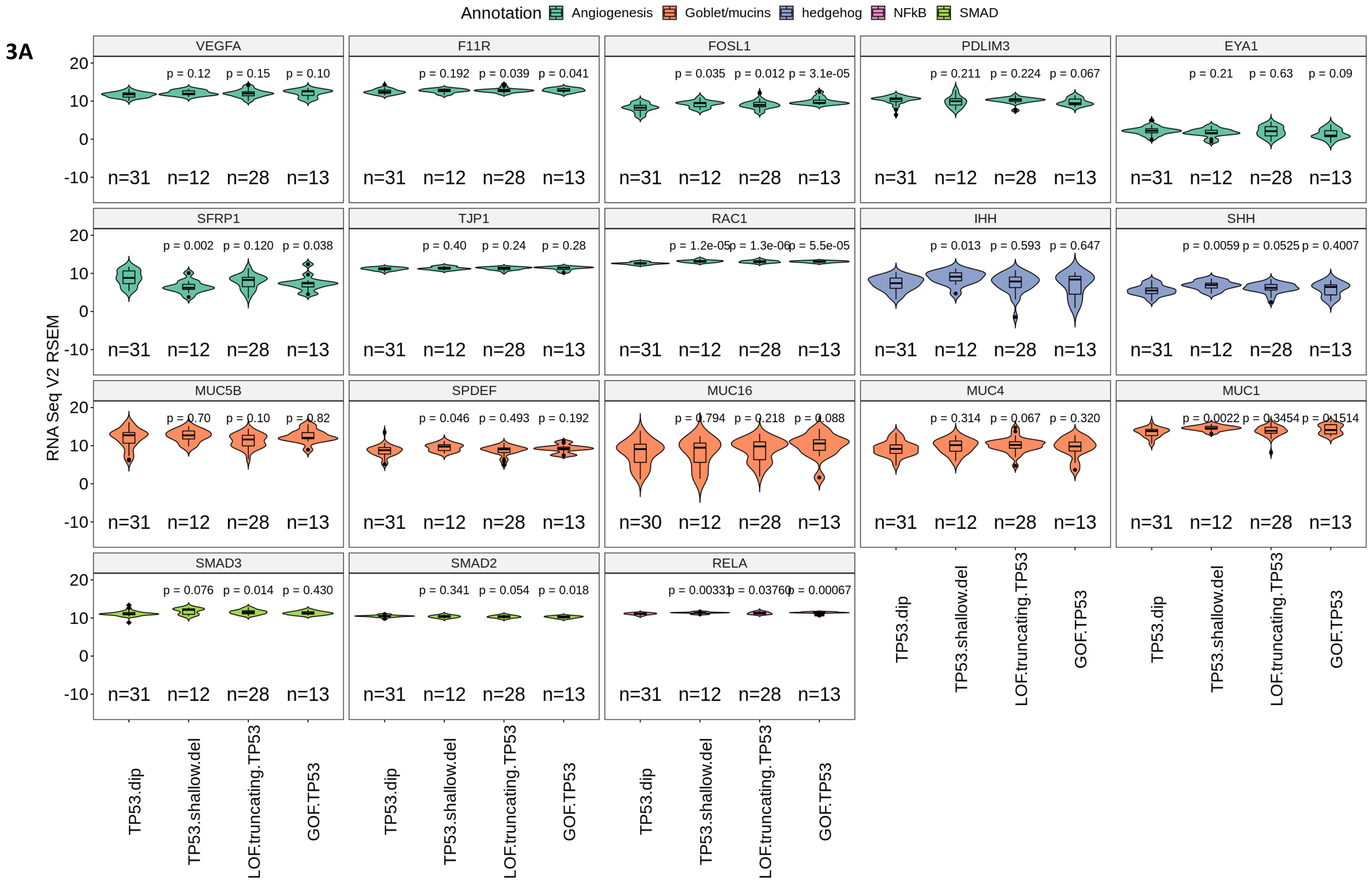

Supplementary figure 3A: The PDAC samples (TCGA-PAAD) with loss-of-TP53 alterations (heterozygous deletion and truncating mutations) show upregulation of hypoxic (ENO2, HK2, ELF3), angiogenesis (F11R, VEGFA, FOSL1,PDLIM3, EYA1, SFPR1) and TGF-beta signaling (SMAD3, SMAD2) relative to samples WT TP53 or TP53 gain-of-function mutations.
