# Supplementary material for: MUC16 and TP53 family co-regulate tumor-stromal heterogeneity in pancreatic adenocarcinoma
Source: Front Oncol. 2023 Feb 3;13:1073820. doi: 10.3389/fonc.2023.1073820 (PMC9936860; doi:10.3389/fonc.2023.1073820)
Supplement: Supplementary file 6 [file DataSheet_3.pdf]

TP63 binding motif (gggcatggtggcacatgcct) in Human MUC16 gene

agtaaaggaaactatcaacagagtaaacagacaatctacagaatgggaga
aaagctttacaaactttgcatctgacaaaggtctaatatccagcatctaa
aagaaacgtgccgggtgtggtggctcacacctgtaatcccagcactttgg
cggatcacctgaggttgggagttcgagaccagcctgcccaacatggagaa
agccaatctctactaaaaatacaaaaattagccgggcatggtggcacatg
cctgtaattccagctactccaggggctgaggcagaagaatcgcttgaacc
cggaaggtggaggttgcagtgagccgagattgcaccactgcactccagcc
tgggtgacagagtgagactccatctcaaaaaaaataaaaaataaaaaata
aacgaatttataatttaaaaaaccagcaaaaaacaaaacaaacaaacaaa
agaaacattacacagtgggcaaaggacgtggttcttcaatcattttgacc
tggataaaattggtagctcctgagtagaaaagaagattgggaaagcatga
ggaatgaattatagtttcacttaccccaccggcctagggaaaaactcaat
tcctaggagacgcagcttagaaccaaggtgggcctcatcttgagagaaat
ccatgagccatgcctatcttagtctgtattgcattgcttatagcagaaca
gctgaaactgggtaatttacaaagaaaaataatgtatctattacagatat
ggaggctggaaagttgaaatccagaggggcacacctggtgagagccctgt
tgctggtggggactctgtggcatccagaggtagacagggcatcacacgtc
aagacagctgagtgcgctaatgtgatagcacaggtctcacttcctatttt
aaagcttttcttttcttttttttgtttgagatggagtctcaatctgtcacc
caggatggagtgcagtgctgcgatcttggctcactgcaacctccacctcc
tgggttcaagtgattttcctctcttagcctcctgagtagctgggaccaca
ggtgcgcaccactatgtctggctaattttttgtatttttttgtagagacat
ggtttcaccatgttggccaggctggtctcgaattcctgacttcaagtaat
ccacccacctcagcctcccaaagtgctgggattacaagcatgagccacca
tgcatggcctaaagcttcttttaaagccaccaagtcccttcccatgttag
cccactaatccatgggttagtcatgaatggattaatctattcatacggac
agagccctcatcacccaatcacctcttaaaggccccacctctcaatactg
ccacactggggattaagtttcaacagagttttggaggggacattcaaatc
atagtaatgcccaaagtgaaaaatcttccctgcacttttccctcaacaaa
aacagccagagatagtgagctgccaggaaattcttttttttttcctcttc
tgtcctaaatcagcatcgctagacctttacatgattcaacctcatcttct
tcaccctctgggtcatgaaatttttatttatttatttattatttttcttggg
acagactctggctctgtcgcccaggctgaagtgcagtggtgtgatcttgg
ctcactgcaacctccgcctcccgggttcaagcgattctcctgcctcagcc
tcctgagtagctgggattacaggtgggcgccaccacacccagctaatttt
ttgtatttttagtagagatggggtttcaccatattagccaggatggtctc
catctcttgacctcgtgatctgcccacctcagcctcccaaaatgctggga
ttacaggcatgagacaccacgcccagcaggccagggtcatgagattttaa
tcaagagcaacttccactgattcctgagagtgcatctgtgggcccctgct
ctgatctgaacagaagtgccgtgtcttctctgacctccacttctcaattc
aagagccttagtatctgccagtatcacacactgagcattagctccatctc
atgggggtgtaggtaggggctctatctgcatctttctttcttttttttctt
tctttcccttcctcccttcctcactccctcggtcctctctttctttcctt
ttctttcttccttcctcccttcctccctccctccctctctctttctctct
ttctttctttccttctttctttctttctctcttccttccctccctccctc

cttccttcctttctctttctttctctttctttctttttttccttccttcc
ttccttctttctctttctctccctcccttccttccttccttccttccttc
cttcctttctttctttctttctttctttctttctttctttctttctttct
ttctttcttccttccttccttccttccttccttccttccttccttccttt
cttttctttctttctctttctttttgagacagagctcttattacccatgc
tggagtgcagtggtgtgaccttggcttactgcaacatctgcctcctaggg
tcaagtgattctcctgcctcagcctcctaagtagctgggattacagacac
atgccaccacacccaatatttatttttattaaatttttttttaaaattat
ttttaaaaaattaaaaataattttgtatttttagtagagacggggtttct
ccatgttggtcaggctgatctcaaactcccaacctcaggtgatcctccca
cctcacctcccaaagtgctgggattacaggcatgagccaccgtgcccagc
ctggttcctggtttctaagacatcacacacacacacacacacacacacac
acactcacacactcagagagagagagagagagaggatcattaagacatga
tacactaagaaattctattctgcagacactgagaatccgttaaaaagttt
gaagggaagaattgagatcatcaggtgtttatttgaggaaattgtctgtg
gttgaactatcctttcctttctctccctgagatttggtcttctcaattag
AAGCGTTGCACAATTCCCCCAACCTCCATACATACGGCAGCTCTTCTAGA
CACAGGTTTTCCCAGGTCAAATGCGGGGACCCCAGCCATATCTCCCACCC
TGAGAAATTTTGGAGTTTCAGGGAGCTCAGAAGCTCTGCAGAGGCCACCC
TCTCTGAGGGGATTCTTCTTAGACCTCCATCCAGAGGCAAATGTTGACCT
GTCCATGCTGAAACCCTCAGGCCTTCCTGGGTCATCTTCTCCCACCCGCT
CCTTGATGACAGGGAGCAGGAGCACTAAAGCCACACCAGAAATGGATTCA
GGACTGACAGGAGCCACCTTGTCACCTAAGACATCTACAGGTGCAATCGT
GGTGACAGAACATACTCTGCCCTTTACTTCCCCAGATAAGACCTTGGCCA
GTCCTACATCTTCGGTTGTGGGAAGAACCACCCAGTCTTTGGGGGTGATG
TCCTCTGCTCTCCCTGAGTCAACCTCTAGAGGAATGACACACTCCGAGCA
AAGAACCAGCCCATCGCTGAGTCCCCAGGTCAATGGAACTCCCTCTAGGA
ACTACCCTGCTACAAGCATGGTTTCAGGATTGAGTTCCCCAAGGACCAGG
ACCAGTTCCACAGAAGGAAATTTTACCAAAGAAGCATCTACATACACACT
CACTGTAGAGACCACAAGTGGCCCAGTCACTGAGAAGTACACAGTCCCCA
CTGAGACCTCAACAACTGAAGGTGACAGCACAGAGACCCCCTGGGACACA
AGATATATTCCTGTAAAAATCACATCTCCAATGAAAACATTTGCAGATTC
AACTGCATCCAAGGAAAATGCCCCAGTGTCTATGACTCCAGCTGAGACCA
CAGTTACTGACTCACATACTCCAGGAAGGACAAACCCATCATTTGGGACA
CTTTATTCTTCCTTCCTTGACCTATCACCTAAAGGGACCCCAAATTCCAG
AGGTGAAACAAGCCTGGAACTGATTCTATCAACCACTGGATATCCCTTCT
CCTCTCCTGAACCTGGCTCTGCAGGACACAGCAGAATAAGTACCAGTGCG
CCTTTGTCATCATCTGCTTCAGTTCTCGATAATAAAATATCAGAGACCAG
CATATTCTCAGGCCAGAGTCTCACCTCCCCTCTGTCTCCTGGGGTGCCCG
AGGCCAGAGCCAGCACAATGCCCAACTCAGCTATCCCTTTTTCCATGACA
CTAAGCAATGCAGAAACAAGTGCCGAAAGGGTCAGAAGCACAATTTCCTC
TCTGGGGACTCCATCAATATCCACAAAGCAGACAGCAGAGACTATCCTTA
CCTTCCATGCCTTCGCTGAGACCATGGATATACCCAGCACCCACATAGCC
AAGACTTTGGCTTCAGAATGGTTGGGAAGTCCAGGTACCCTTGGTGGCAC
CAGCACTTCAGCGCTGACAACCACATCTCCATCTACCACTTTAGTCTCAG
AGGAGACCAACACCCATCACTCCACGAGTGGAAAGGAAACAGAAGGAACT
TTGAATACATCTATGACTCCACTTGAGACCTCTGCTCCTGGAGAAGAGTC

CGAAATGACTGCCACCTTGGTCCCCACTCTAGGTTTTACAACTCTTGACA
GCAAGATCAGAAGTCCATCTCAGGTCTCTTCATCCCACCCAACAAGAGAG
CTCAGAACCACAGGCAGCACCTCTGGGAGGCAGAGTTCCAGCACAGCTGC
CCACGGGAGCTCTGACATCCTGAGGGCAACCACTTCCAGCACCTCAAAAG
CATCATCATGGACCAGTGAAAGCACAGCTCAGCAATTTAGTGAACCCCAG
CACACACAGTGGGTGGAGACAAGTCCTAGCATGAAAACAGAGAGACCCCC
AGCATCAACCAGTGTGGCAGCCCCTATCACCACTTCTGTTCCCTCAGTGG
TCTCTGGCTTCACCACCCTGAAGACCAGCTCCACAAAAGGGATTTGGCTT
GAAGAAACATCTGCAGACACACTCATCGGAGAATCCACAGCTGGCCCAAC
CACCCATCAGTTTGCTGTTCCCACTGGGATTTCAATGACAGGAGGCAGCA
GCACCAGGGGAAGCCAGGGCACAACCCACCTACTCACCAGAGCCACAGCA
TCATCTGAGACATCCGCAGATTTGACTCTGGCCACGAACGGTGTCCCAGT
CTCCGTGTCTCCAGCAGTGAGCAAGACGGCTGCTGGCTCAAGTCCTCCAG
GAGGGACAAAGCCATCATATACAATGGTTTCTTCTGTCATCCCTGAGACA
TCATCTCTACAGTCCTCAGCTTTCAGGGAAGGAACCAGCCTGGGACTGAC
TCCATTAAACACTAGACATCCCTTCTCTTCCCCTGAACCAGACTCTGCAG
GACACACCAAGATAAGCACCAGCATTCCTCTGTTGTCATCTGCTTCAGTT
CTTGAGGATAAAGTGTCAGCGACCAGCACATTCTCACACCACAAAGCCAC
CTCATCTATTACCACAGGGACTCCTGAAATCTCAACAAAGACAAAGCCCA
GCTCAGCCGTTCTTTCCTCCATGACCCTAAGCAATGCAGCAACAAGTCCT
GAAAGAGTCAGAAATGCAACTTCCCCTCTGACTCATCCATCTCCATCAGG
GGAAGAGACAGCAGGGAGTGTCCTCACTCTCAGCACCTCTGCTGAGACTA
CAGACTCACCTAACATCCACCCAACTGGGACACTGACTTCAGAATCGTCA
GAGAGTCCTAGCACTCTCAGCCTCCCAAGTGTCTCTGGAGTCAAAACCAC
ATTTTCTTCATCTACTCCTTCCACTCATCTATTTACTAGTGGAGAAGAAA
CAGAGGAAACTTCGAATCCATCTGTGTCTCAACCTGAGACTTCTGTTTCC
AGAGTAAGGACCACCTTGGCCAGCACCTCTGTCCCTACCCCAGTATTCCC
CACCATGGACACCTGGCCTACACGTTCAGCTCAGTTCTCTTCATCCCACC
TAGTGAGTGAGCTCAGAGCTACGAGCAGTACCTCAGTTACAAACTCAACT
GGTTCAGCTCTTCCTAAAATATCTCACCTCACTGGGACGGCAACAATGTC
ACAGACCAATAGAGACACGTTTAATGACTCTGCTGCACCCCAAAGCACAA
CTTGGCCAGAGACTAGTCCCAGATTCAAGACAGGGTTACCTTCAGCAACA
ACCACTGTTTCAACCTCTGCCACTTCTCTCTCTGCTACTGTAATGGTCTC
TAAATTCACTTCTCCAGCAACTAGTTCCATGGAAGCAACTTCTATCAGGG
AACCATCAACAACCATCCTCACAACAGAGACCACGAATGGCCCAGGCTCT
ATGGCTGTGGCTTCTACCAACATCCCAATTGGAAAGGGCTACATTACTGA
AGGAAGATTGGACACAAGCCATCTGCCCATTGGAACCACAGCTTCCTCTG
AGACATCTATGGATTTTACCATGGCCAAAGAAAGTGTCTCAATGTCAGTA
TCTCCATCTCAGTCCATGGATGCTGCTGGCTCAAGCACTCCAGGAAGGAC
AAGCCAATTCGTTGACACATTTTCTGATGATGTCTATCATTTAACATCCA
GAGAAATTACAATACCTAGAGATGGAACAAGCTCAGCTCTGACTCCACAA
ATGACTGCAACTCACCCTCCATCTCCTGATCCTGGCTCTGCTAGAAGCAC
CTGGCTTGGCATCTTGTCCTCATCTCCTTCTTCTCCTACTCCCAAAGTCA
CAATGAGCTCCACATTTTCAACTCAGAGAGTCACCACAAGCATGATAATG
GACACAGTTGAAACTAGTCGGTGGAACATGCCCAACTTACCTTCCACGAC
TTCCTTGACACCAAGTAATATTCCAACAAGTGGTGCCATAGGAAAAAGCA
CCCTGGTTCCCTTGGACACTCCATCTCCAGCCACATCATTGGAGGCATCA

GAAGGGGGACTTCCAACCCTCAGCACCTACCCTGAATCAACAAACACACC
CAGCATCCACCTCGGAGCACACGCTAGTTCAGAAAGTCCAAGCACCATCA
AACTTACCATGGCTTCAGTAGTAAAACCTGGCTCTTACACACCTCTCACC
TTCCCCTCAATAGAGACCCACATTCATGTATCAACAGCCAGAATGGCTTA
CTCTTCTGGGTCTTCACCTGAGATGACAGCTCCTGGAGAGACTAACACTG
GTAGTACCTGGGACCCCACCACCTACATCACCACTACGGATCCTAAGGAT
ACAAGTTCAGCTCAGGTCTCTACACCCCACTCAGTGAGGACACTCAGAAC
CACAGAAAACCATCCAAAGACAGAGTCCGCCACCCCAGCTGCTTACTCTG
GAAGTCCTAAAATCTCAAGTTCACCCAATCTCACCAGTCCGGCCACAAAA
GCATGGACCATCACAGACACAACTGAACACTCCACTCAATTACATTACAC
AAAATTGGCAGAAAAATCATCTGGATTTGAGACACAGTCAGCTCCAGGAC
CTGTCTCTGTAGTAATCCCTACCTCCCCTACCATTGGAAGCAGCACATTG
GAACTAACTTCTGATGTCCCAGGGGAACCCCTGGTCCTTGCTCCCAGTGA
GCAGACCACAATCACTCTCCCCATGGCAACATGGCTGAGTACCAGTTTGA
CAGAGGAAATGGCTTCAACAGACCTTGATATTTCAAGTCCAAGTTCACCC
ATGAGTACATTTGCTATTTTTCCACCTATGTCCACACCTTCTCATGAACT
TTCAAAGTCAGAGGCAGATACCAGTGCCATTAGAAATACAGATTCAACAA
CGTTGGATCAGCACCTAGGAATCAGGAGTTTGGGCAGAACTGGGGACTTA
ACAACTGTTCCTATCACCCCACTGACAACCACGTGGACCAGTGTGATTGA
ACACTCAACACAAGCACAGGACACCCTTTCTGCAACGATGAGTCCTACTC
ACGTGACACAGTCACTCAAAGATCAAACATCTATACCAGCCTCAGCATCC
CCTTCCCATCTTACTGAAGTCTACCCTGAGCTCGGGACACAAGGGAGAAG
CTCCTCTGAGGCAACCACTTTTTGGAAACCATCTACAGACACACTGTCCA
GAGAGATTGAGACTGGCCCAACAAACATTCAATCCACTCCACCCATGGAC
AACACAACAACAGGGAGCAGTAGTAGTGGAGTCACCCTGGGCATAGCCCA
CCTTCCCATAGGAACATCCTCCCCAGCTGAGACATCCACAAACATGGCAC
TGGAAAGAAGAAGTTCTACAGCCACTGTCTCTATGGCTGGGACAATGGGA
CTCCTTGTTACTAGTGCTCCAGGAAGAAGCATCAGCCAGTCATTAGGAAG
AGTTTCCTCTGTCCTTTCTGAGTCAACTACTGAAGGAGTCACAGATTCTA
GTAAGGGAAGCAGCCCAAGGCTGAACACACAGGGAAATACAGCTCTCTCC
TCCTCTCTTGAACCCAGCTATGCTGAAGGAAGCCAGATGAGCACAAGCAT
CCCTCTAACCTCATCTCCTACAACTCCTGATGTGGAATTCATAGGGGGCA
GCACATTTTGGACCAAGGAGGTCACCACAGTTATGACCTCAGACATCTCC
AAGTCTTCAGCAAGGACAGAGTCCAGCTCAGCTACCCTTATGTCCACAGC
TTTGGGAAGCACTGAAAATACAGGAAAAGAAAAACTCAGAACTGCCTCTA
TGGATCTTCCATCTCCAACTCCATCAATGGAGGTGACACCATGGATTTCT
CTCACTCTCAGTAATGCCCCCAATACCACAGATTCACTTGACCTCAGCCA
TGGGGTGCACACCAGCTCTGCAGGGACTTTGGCCACTGACAGGTCATTGA
ATACTGGTGTCACTAGAGCCTCCAGATTGGAAAACGGCTCTGATACCTCT
TCTAAGTCCCTGTCTATGGGAAACAGCACTCACACTTCCATGACTTACAC
AGAGAAGAGTGAAGTGTCTTCTTCAATCCATCCCCGACCTGAGACCTCAG
CTCCTGGAGCAGAGACCACTTTGACTTCCACTCCTGGAAACAGGGCCATA
AGCTTAACATTGCCTTTTTCATCCATTCCAGTGGAAGAAGTCATTTCTAC
AGGCATAACCTCAGGACCAGACATCAACTCAGCACCCATGACACATTCTC
CCATCACCCCACCAACAATTGTATGGACCAGTACAGGCACAATTGAACAG
TCCACTCAACCACTACATGCAGTTTCTTCAGAAAAAGTTTCTGTGCAGAC
ACAGTCAACTCCATATGTCAACTCTGTGGCAGTGTCTGCTTCCCCTACCC

ATGAGAATTCAGTCTCTTCTGGAAGCAGCACATCCTCTCCATATTCCTCA
GCCTCACTTGAATCCTTGGATTCCACAATCAGTAGGAGGAATGCAATCAC
TTCCTGGCTATGGGACCTCACTACATCTCTCCCCACTACAACTTGGCCAA
GTACTAGTTTATCTGAGGCACTGTCCTCAGGCCATTCTGGGGTTTCAAAC
CCAAGTTCAACTACGACTGAATTTCCACTCTTTTCAGCTGCATCCACATC
TGCTGCTAAGCAAAGAAATCCAGAAACAGAGACCCATGGTCCCCAGAATA
CAGCCGCGAGTACTTTGAACACTGATGCATCCTCGGTCACAGGTCTTTCT
GAGACTCCTGTGGGGGCAAGTATCAGCTCTGAAGTCCCTCTTCCAATGGC
CATAACTTCTAGATCAGATGTTTCTGGCCTTACATCTGAGAGTACTGCTA
ACCCGAGTTTAGGCACAGCCTCTTCAGCAGGGACCAAATTAACTAGGACA
ATATCCCTGCCCACTTCAGAGTCTTTGGTTTCCTTTAGAATGAACAAGGA
TCCATGGACAGTGTCAATCCCTTTGGGGTCCCATCCAACTACTAATACAG
AAACAAGCATCCCAGTAAACAGCGCAGGTCCACCTGGCTTGTCCACAGTA
GCATCAGATGTAATTGACACACCTTCAGATGGGGCTGAGAGTATTCCCAC
TGTCTCCTTTTCCCCCTCCCCTGATACTGAAGTGACAACTATCTCACATT
TCCCAGAAAAGACAACTCATTCATTTAGAACCATTTCATCTCTCACTCAT
GAGTTGACTTCAAGAGTGACACCTATTCCTGGGGATTGGATGAGTTCAGC
TATGTCTACAAAGCCCACAGGAGCCAGTCCCTCCATTACACTGGGAGAGA
GAAGGACAATCACCTCTGCTGCTCCAACCACTTCCCCCATAGTTCTCACT
GCTAGTTTCACAGAGACCAGCACAGTTTCACTGGATAATGAAACTACAGT
AAAAACCTCAGATATCCTTGACGCACGGAAAACAAATGAGCTCCCCTCAG
ATAGCAGTTCTTCTTCTGATCTGATCAACACCTCCATAGCTTCTTCAACT
ATGGATGTCACTAAAACAGCCTCCATCAGTCCCACTAGCATCTCAGGAAT
GACAGCAAGTTCCTCCCCATCTCTCTTCTCTTCAGATAGACCCCAGGTTC
CCACATCTACAACAGAGACAAATACAGCCACCTCTCCATCTGTTTCCAGT
AACACCTATTCTCTTGATGGGGGCTCCAATGTGGGTGGCACTCCATCCAC
TTTACCACCCTTTACAATCACCCACCCTGTCGAGACAAGCTCGGCCCTAT
TAGCCTGGTCTAGACCAGTAAGAACTTTCAGCACCATGGTCAGCACTGAC
ACTGCCTCCGGAGAAAATCCTACCTCTAGCAATTCTGTGGTGACTTCTGT
TCCAGCACCAGGTACATGGACCAGTGTAGGCAGTACTACTGACTTACCTG
CCATGGGCTTTCTCAAGACAAGTCCTGCAGGAGAGGCACACTCACTTCTA
GCATCAACTATTGAACCAGCCACTGCCTTCACTCCCCATCTCTCAGCAGC
AGTGGTCACTGGATCCAGTGCTACATCAGAAGCCAGTCTTCTCACTACGA
GTGAAAGCAAAGCCATTCATTCTTCACCACAGACCCCAACTACACCCACC
TCTGGAGCAAACTGGGAAACTTCAGCTACTCCTGAGAGCCTTTTGGTAGT
CACTGAGACTTCAGACACAACACTTACCTCAAAGATTTTGGTCACAGATA
CCATCTTGTTTTCAACTGTGTCCACGCCACCTTCTAAATTTCCAAGTACG
GGGACTCTGTCTGGAGCTTCCTTCCCTACTTTACTCCCGGACACTCCAGC
CATCCCTCTCACTGCCACTGAGCCAACAAGTTCATTAGCTACATCCTTTG
ATTCCACCCCACTGGTGACTATAGCTTCGGATAGTCTTGGCACAGTCCCA
GAGACTACCCTGACCATGTCAGAGACCTCAAATGGTGATGCACTGGTTCT
TAAGACAGTAAGTAACCCAGATAGGAGCATCCCTGGAATCACTATCCAAG
GAGTAACAGAAAGTCCACTCCATCCTTCTTCCACTTCCCCCTCTAAGATT
GTTGCTCCACGGAATACAACCTATGAAGGTTCGATCACAGTGGCACTTTC
TACTTTGCCTGCGGGAACTACTGGTTCCCTTGTATTCAGTCAGAGTTCTG
AAAACTCAGAGACAACGGCTTTGGTAGACTCATCAGCTGGGCTTGAGAGG
GCATCTGTGATGCCACTAACCACAGGAAGCCAGGGTATGGCTAGCTCTGG

AGGAATCAGAAGTGGGTCCACTCACTCAACTGGAACCAAAACATTTTCTT
CTCTCCCTCTGACCATGAACCCAGGTGAGGTTACAGCCATGTCTGAAATC
ACCACGAACAGACTGACAGCTACTCAATCAACAGCACCCAAAGGGATACC
TGTGAAGCCCACCAGTGCTGAGTCAGGCCTCCTAACACCTGTCTCTGCCT
CCTCAAGCCCATCAAAGGCCTTTGCCTCACTGACTACAGCTCCCCCAACT
TGGGGGATCCCACAGTCTACCTTGACATTTGAGTTTTCTGAGGTCCCAAG
TTTGGATACTAAGTCCGCTTCTTTACCAACTCCTGGACAGTCCCTGAACA
CCATTCCAGACTCAGATGCAAGCACAGCATCTTCCTCACTGTCCAAGTCT
CCAGAAAAAAACCCAAGGGCAAGGATGATGACTTCCACAAAGGCCATAAG
TGCAAGCTCATTTCAATCAACAGGTTTTACTGAAACCCCTGAGGGATCTG
CCTCCCCTTCTATGGCAGGGCATGAACCCAGAGTCCCCACTTCAGGAACA
GGGGACCCTAGATATGCCTCAGAGAGCATGTCTTATCCAGACCCAAGCAA
GGCATCATCAGCTATGACATCGACCTCTCTTGCATCAAAACTCACAACTC
TCTTCAGCACAGGTCAAGCAGCAAGGTCTGGTTCTAGTTCCTCTCCCATA
AGCCTATCCACTGAGAAAGAAACAAGCTTCCTTTCCCCCACTGCATCCAC
CTCCAGAAAGACTTCACTATTTCTTGGGCCTTCCATGGCAAGGCAGCCCA
ACATATTGGTGCATCTTCAGACTTCAGCTCTGACACTTTCTCCAACATCC
ACTCTAAATATGTCCCAGGAGGAGCCTCCTGAGTTAACCTCAAGCCAGAC
CATTGCAGAAGAAGAGGGAACAACAGCTGAAACACAGACGTTAACCTTCA
CACCATCTGAGACCCCAACATCCTTGTTACCTGTCTCTTCTCCCACAGAA
CCCACAGCCAGAAGAAAGAGTTCTCCAGAAACATGGGCAAGCTCTATTTC
AGTTCCTGCCAAGACCTCCTTGGTTGAAAgtaagaatgccctgctccttc
cccaagtgtgctggggatgaatctggaaataaactacatctttttttattt
tttaaacttttatatttgaaaatataaatattttaggttcagggaacatg
tgcaggtttgttatataggtaaattgcatgtcatgggggcttggggtaca
gattacatcatcagccaggtaataagcctagtacctgatcagtagatttt
ttttaatcctctccctcctcccagcctccaccctcaattcacatgtctcc
atgtgtactcaaggtttaattcccacttatgagtgagaacatgcggtatt
tgtaaactacatctttatttttgctaacctcgaactgaaatttagcattt
gttttattgatgaatagaggtaacaaaacaaaccacattaatcctagcag
tgcctgtgcctttgccaacaacagaaattccggacactttcatatcctat
gacaattgttgcaagcacttttaaaaatcatgtacgactttattcataat
tatagtggttattaggcttttcaatagatcttatttaatgagttagtaaa
ataagtgcctgtattattgtattacatttgtttattaagatcttgataac
aacatttcaatataatcatttcctttgtttttttaaattttagattcaggg
gtatatgtgcaggtttgttacgtggatatactgcataatgatgaggtttg
gcttctagtgaacccatcagccaaatagtgaatgttgtgcccaataagta
gtttttcaatcctcacttcactcccagcctcctctattttggagtcccag
tgtctattatttctatctttatgtccacatgtacccattggttagctccc
acttataagtgagaatgtgcagtatttaattttctgtttttgagttattt
tgcttaggttgatggccttcagctccagccacgttgctttaaagaacatg
atttcattctttttttatggctgcatagtactccgaggtgtatgtgtacca
gattttctttatccacaatgatttcctttgtaatctaatattttatattg
ttattttatgttttattctatatttttattttaatttataaaggaattca
tatggttcacaagcctgtcaaagggacctataataaaaagaggttaagaa
tccatgctctaaacagaatattactccatttatttcatttatttttaaa
gagacagtctcactctgtcatccaggctggagtacagtggagtgatcata

gctcattgcaaccctgaactcttgggcacaagcaattctcctgcttcatc
ctccagaggagctgggactacaggtgcacatcaccatgcccagctagttt
taaaaattattttgtagagatggtgtctcactatcctacccaggctggtc
tcaaactcctgggctcaggcaatcctcccactttgacctcccaaagtgtt
gagattacaggggcaagccactgtgcctggccacttgtcacattttaatt
tgtgattacttataaaatgaaccccttcccatctgagatctgtcagtctt
tctggtgacggtgcctggtgtctgctttctaccatgtcctgttagactag
tgtttgatgggaggtcacctgggcagctgtccagctcactcactgggctc
tagagcctctgagttgaagcaaaatagaaagatcagtcaatgtaaagaaa
gctcaaaaactgacattctgaagtaatggatagctaaaccttcctattgc
ccttttctttcagCAACTGATGGAACGCTAGTGACCACCATAAAGATGTC
AAGCCAGGCAGCACAAGGAAATTCCACGTGGCCTGCCCCAGCAGAGGAGA
CGGGGAGCAGTCCAGCAGgtaaatatagaccttgtttccatttctgctct
gctaatgccacccaagcctttcttttcttttcttttcttttcttttcttt
tcttttcttttcttttcttttctttctctccctttctttctttctttctt
tctttctttctttctttctttctttctttctttctctttctttctttctt
tctttctttctttctttctctttctttcttctttctctctctctctttct
ttctttctcttgttcttttaaatttttttatttttttacttaatttttttt
cacccaagccttaaggccaatttggaccagatagtgagaccccacctcta
taaaaaaatttttttttaaaaaaaaataagttgggcatcgtgcaggcctgta
gtccctgctactcgagaggccaaggtgggaggacagcttgctgctgacta
aaagtgctgcttattgattctgggaagaaaaaaatatacaaggcttcagtt
tcattattttataagtaaatgctagcaacttttcctttctttctctcttt
ctctcttcctctctttctctcctctccttctcttctctctctctctctct
ctctttctctctcctctccttctcttctcttctttctctctctctctctt
tcatttatttttgagacatggtctcattctgtcacccaggctggagtaca
gtggtgtatatttactgcagtactcactgtactcactgcagcctcaaatt
cctgggctcaagctatcctctcacctcagcctcctgagtagctgggcagc
agtccagctcactcactgggctctagagcctctgtgctatgcccagctta
ttgttgttgttttttaaatttttttttttgtacagatggggtctcacta
tgtggcccaaggtggtcttaaactcctggctccaagagatcctcccacct
cagcctcccaaagtgcagggattacaggtgtgagccactgtgcccagcct
agaccgcatttttttttttgaaacagggtctccctctgttgcccaggctgg
agtgcaatggcgtgttcatggttcactgcagcctcagcctcctcagtctc
aagcaatcctccaacttcagcctcccccaacagctagaactgcaggtgat
catcaccaattagcctggttaattgtgtgtgtatttcttaaatttttttgt
agagatagttctcactatattgcttgggctggtctcaaactcctggactc
aagtgattcacctacctcggcctccctaagcactgggattacaggcttga
gccaccacacccagcaaggactaggttttaaaataggttcctaggctggg
tgtggtggcttacgcccgtaatcccagcactttgggaggctaaggtgggc
ggatcacgaggtcaggagtttgagaccagcctggccaacatagtgaaacc
ctgtctctactaaaaatacaaaaaaattagctgggcatagtggcacacacc
tgtaatcccagctacttgggaggctgaggaaggagaatcacttgaacctg
ggaggcggaggttgcagtgagccgagatcacgccattgctctccagcctg
ggtgacagagcaagactccatctaaaaaaaaaaaaaagttcctttgacttc
ttgacactcttctctgaggatattgatcatttttccccaatagatgttac
taattgaacacttctgttgcttcaacttactaatttacatgatcaatagc

caattaattcagcaggagagaatgctacagagtcgattctttctgtactt
tcttctgctccagagtgaaggatctttctaaatcagagaccatcactgtg
ttcacagggagggcctaggtgaacctgagatggcaaatgttgcgtttgtt
actacggaagaagggattatgggctgaagtccttggcagtgccaaattgc
ttagaaaaatgtgaaatatggtccctaggagtgctcttgggatgtcacat
ttttctcactcctttgacaggtagatgttattttcctgaaggacagggaa
aggattcagagggaggaatgaatttgaaagaaaatgaaggtgacgagaaa
gaatgagctcatctcccttatcctctttcttctcaaatccttaagtagct
ttgcagtgaactaagatttgggggaacctagaggaggctgaaagttggaa
gctgaaattggtttggcaagggcaagctccaaagacaaaagtggaaatag
tttgggggtagcctcttgcatgggtgaagtcctggttcatcacatcctcc
cttatgcaaagagcccttttatatggggcatggggaaaaactgagctaaa
ggtgataatttctcctgagcaagccagatggtcaaagctctaacttcacc
atctcccttggaatgtttaatgtgttccctggtgtccagaggcttaaagt
gtgagaattaaaagctcaacattttctttcccagagaaggaggaaatagt
tttaattgaaatcccgggaggaaatgaatgatagtgtcaaaccaaaaaac
ttcatcttctgtaccatttgcatatactccactgacttactttctaatca
cagGCACATCCCCAGGAAGCCCAGAAATGTCTACCACTCTCAAAATCATG
AGCTCCAAGGAACCCAGCATCAGCCCAGAGATCAGGTCCACTGTGAGAAA
TTCTCCTTGGAAGACTCCAGAAACAACTGTTCCCATGGAGACCACAGTGG
AACCAGTCACCCTTCAGTCCACAGCCCTAGGAAGTGGCAGCACCAGCATC
TCTCACCTGCCCACAGGAACCACATCACCAACCAAGTCACCAACAGAAAA
TATGTTGGCTACAGAAAGGGTCTCCCTCTCCCCATCCCCACCTGAGGCTT
GGACCAACCTTTATTCTGGAACTCCAGGAGGGACCAGGCAGTCACTGGCC
ACAATGTCCTCTGTCTCCCTAGAGTCACCAACTGCTAGAAGCATCACAGG
GACTGGTCAGCAAAGCAGTCCAGAACTGGTTTCAAAGACAACTGGAATGG
AATTCTCTATGTGGCATGGCTCTACTGGAGGGACCACAGGGGACACACAT
GTCTCTCTGAGCACATCTTCCAATATCCTTGAAGACCCTGTAACCAGCCC
AAACTCTGTGAGCTCATTGACAGATAAATCCAAACATAAAACCGAGACAT
GGGTAAGCACCACAGCCATTCCCTCCACTGTCCTGAATAATAAGATAATG
GCAGCTGAACAACAGACAAGTCGATCTGTGGATGAGGCTTATTCATCAAC
TAGTTCTTGGTCAGATCAGACATCTGGGAGTGACATCACCCTTGGTGCAT
CTCCTGATGTCACAAACACATTATACATCACCTCCACAGCACAAACCACC
TCACTAGTGTCTCTGCCCTCTGGAGACCAAGGCATTACAAGCCTCACCAA
TCCCTCAGGAGGAAAAACAAGCTCTGCGTCATCTGTCACATCTCCTTCAA
TAGGGCTTGAGACTCTGAGGGCCAATGTAAGTGCAGTGAAAAGTGACATT
GCCCCTACTGCTGGGCATCTATCTCAGACTTCATCTCCTGCGGAAGTGAG
CATCCTGGACGTAACCACAGCTCCTACTCCAGGTATCTCCACCACCATCA
CCACCATGGGAACCAACTCAATCTCAACTACCACACCCAACCCAGAAGTG
GGTATGAGTACCATGGACAGCACCCCGGCCACAGAGAGGCGCACAACTTC
TACAGAACACCCTTCCACCTGGTCTTCCACAGCTGCATCAGATTCCTGGA
CTGTCACAGACATGACTTCAAACTTGAAAGTTGCAAGATCTCCTGGAACA
ATTTCCACAATGCATACAACTTCATTCTTAGCCTCAAGCACTGAATTAGA
CTCCATGTCTACTCCCCATGGCCGTATAACTGTCATTGGAACCAGCCTGG
TCACTCCATCCTCTGATGCTTCAGCTGTAAAGACAGAGACCAGTACAAGT
GAAAGAACATTGAGTCCTTCAGACACAACTGCATCTACTCCCATCTCAAC
TTTTTCTCGTGTCCAGAGGATGAGCATCTCAGTTCCTGACATTTTAAGTA

CAAGTTGGACTCCCAGTAGTACAGAAGCAGAAGATGTGCCTGTTTCAATG
GTTTCTACAGATCATGCTAGTACAAAGACTGACCCAAATACGCCCCTGTC
CACTTTTCTGTTTGATTCTCTGTCCACTCTTGACTGGGACACTGGGAGAT
CTCTGTCATCAGCCACAGCCACTACCTCAGCTCCTCAGGGGGCCACAACT
CCCCAGGAACTCACTTTGGAAACCATGATCAGCCCAGCTACCTCACAGTT
GCCCTTCTCTATAGGGCACATTACAAGTGCAGTCACACCAGCTGCAATGG
CAAGGAGCTCTGGAGTTACTTTTTCAAGACCAGATCCCACAAGCAAAAAG
GCAGAGCAGACTTCCACTCAGCTTCCCACCACCACTTCTGCACATCCAGG
GCAGGTGCCCAGATCAGCAGCAACAACTCTGGATGTGATCCCACACACAG
CAAAAACTCCAGATGCAACTTTTCAGAGACAAGGGCAGACAGCTCTTACA
ACAGAGGCAAGAGCTACATCTGACTCCTGGAATGAGAAAGAAAAATCAAC
CCCAAGTGCACCTTGGATCACTGAGATGATGAATTCTGTCTCAGAAGATA
CCATCAAGGAGGTTACCAGCTCCTCCAGTGTATTAAGGACCCTGAATACG
CTGGACATAAACTTGGAATCTGGGACGACTTCATCCCCAAGTTGGAAAAG
CAGCCCATATGAGAGAATTGCCCCTTCTGAGTCCACCACAGACAAAGAGG
CAATTCACCCTTCTACAAACACAGTAGAGACCACAGGCTGGGTCACAAGT
TCCGAACATGCTTCTCATTCCACTATCCCAGCCCACTCAGCGTCATCCAA
ACTCACATCTCCAGTGGTTACAACCTCCACCAGGGAACAAGCAATAGTTT
CTATGTCAACAACCACATGGCCAGAGTCTACAAGGGCTAGAACAGAGCCT
AATTCCTTCTTGACTATTGAACTGAGGGACGTCAGCCCTTACATGGACAC
CAGCTCAACCACACAAACAAGTATTATCTCTTCCCCAGGTTCCACTGCGA
TCACCAAGGGGCCTAGAACAGAAATTACCTCCTCTAAGAGAATATCCAGC
TCATTCCTTGCCCAGTCTATGAGGTCGTCAGACAGCCCCTCAGAAGCCAT
CACCAGGCTGTCTAACTTTCCTGCCATGACAGAATCTGGAGGAATGATCC
TTGCTATGCAAACAAGTCCACCTGGCGCTACATCACTAAGTGCACCTACT
TTGGATACATCAGCCACAGCCTCCTGGACAGGGACTCCACTGGCTACGAC
TCAGAGATTTACATACTCAGAGAAGACCACTCTCTTTAGCAAAGGTCCTG
AGGATACATCACAGCCAAGCCCTCCCTCTGTGGAAGAAACCAGCTCTTCC
TCTTCCCTGGTACCTATCCATGCTACAACCTCGCCTTCCAATATTTTGTT
GACATCACAAGGGCACAGTCCCTCCTCTACTCCACCTGTGACCTCAGTTT
TCTTGTCTGAGACCTCTGGCCTGGGGAAGACCACAGACATGTCGAGGATA
AGCTTGGAACCTGGCACAAGTTTACCTCCCAATTTGAGCAGTACAGCAGG
TGAGGCGTTATCCACTTATGAAGCCTCCAGAGATACAAAGGCAATTCATC
ATTCTGCAGACACAGCAGTGACGAATATGGAGGCAACCAGTTCTGAATAT
TCTCCTATCCCAGGCCATACAAAGCCATCCAAAGCCACATCTCCATTGGT
TACCTCCCACATCATGGGGGACATCACTTCTTCCACATCAGTATTTGGCT
CCTCCGAGACCACAGAGATTGAGACAGTGTCCTCTGTGAACCAGGGACTT
CAGGAGAGAAGCACATCCCAGGTGGCCAGCTCTGCTACAGAGACAAGCAC
TGTCATTACCCATGTGTCTAGTGGTGATGCTACTACTCATGTCACCAAGA
CACAAGCCACTTTCTCTAGCGGAACATCCATCTCAAGCCCTCATCAGTTT
ATAACTTCTACCAACACATTTACAGATGTGAGCACCAACCCCTCCACCTC
TCTGATAATGACAGAATCTTCAGGAGTGACCATCACCACCCAAACAGGTC
CTACTGGAGCTGCAACACAGGGTCCATATCTCTTGGACACATCAACCATG
CCTTACTTGACAGAGACTCCATTAGCTGTGACTCCAGATTTTATGCAATC
AGAGAAGACCACTCTCATAAGCAAAGGTCCCAAGGATGTGTCCTGGACAA
GCCCTCCCTCTGTGGCAGAAACCAGCTATCCCTCTTCCCTGACACCTTTC
TTGGTCACAACCATACCTCCTGCCACTTCCACGTTACAAGGGCAACATAC

ATCCTCTCCTGTTTCTGCGACTTCAGTTCTTACCTCTGGACTGGTGAAGA
CCACAGATATGTTGAACACAAGCATGGAACCTGTGACCAATTCACCTCAA
AATTTGAACAATCCATCAAATGAGATACTGGCCACTTTGGCAGCCACCAC
AGATATAGAGACTATTCATCCTTCCATAAACAAAGCAGTGACCAATATGG
GGACTGCCAGTTCAGCACATGTACTGCATTCCACTCTCCCAGTCAGCTCA
GAACCATCTACAGCCACATCTCCAATGGTTCCTGCCTCCAGCATGGGGGA
CGCTCTTGCTTCTATATCAATACCTGGTTCTGAGACCACAGACATTGAGG
GAGAGCCAACATCCTCCCTGACTGCTGGACGAAAAGAGAACAGCACCCTC
CAGGAGATGAACTCAACTACAGAGTCAAACATCATCCTCTCCAATGTGTC
TGTGGGGGCTATTACTGAAGCCACAAAAATGGAAGTCCCCTCTTTTGATG
CAACATTCATACCAACTCCTGCTCAGTCAACAAAGTTCCCAGATATTTTC
TCAGTAGCCAGCAGTAGACTTTCAAACTCTCCTCCCATGACAATATCTAC
CCACATGACCACCACCCAGACAGGGTCTTCTGGAGCTACATCAAAGATTC
CACTTGCCTTAGACACATCAACCTTGGAAACCTCAGCAGGGACTCCATCA
GTGGTGACTGAGGGGTTTGCCCACTCAAAAATAACCACTGCAATGAACAA
TGATGTCAAGGACGTGTCACAGACAAACCCTCCCTTTCAGGATGAAGCCA
GCTCTCCCTCTTCTCAAGCACCTGTCCTTGTCACAACCTTACCTTCTTCT
GTTGCTTTCACACCGCAATGGCACAGTACCTCCTCTCCTGTTTCTATGTC
CTCAGTTCTTACTTCTTCACTGGTAAAGACCGCAGGCAAGGTGGATACAA
GCTTAGAAACAGTGACCAGTTCACCTCAAAGTATGAGCAACACTTTGGAT
GACATATCGGTCACTTCAGCAGCCACCACAGATATAGAGACAACGCATCC
TTCCATAAACACAGTAGTTACCAATGTGGGGACCACCGGTTCAGCATTTG
AATCACATTCTACTGTCTCAGCTTACCCAGAGCCATCTAAAGTCACATCT
CCAAATGTTACCACCTCCACCATGGAAGACACCACAATTTCCAGATCAAT
ACCTAAATCCTCTAAGACTACAAGAACTGAGACTGAGACAACTTCCTCCC
TGACTCCTAAACTGAGGGAGACCAGCATCTCCCAGGAGATCACCTCGTCC
ACAGAGACAAGCACTGTTCCTTACAAAGAGCTCACTGGTGCCACTACCGA
GGTATCCAGGACAGATGTCACTTCCTCTAGCAGTACATCCTTCCCTGGCC
CTGATCAGTCCACAGTGTCACTAGACATCTCCACAGAAACCAACACCAGG
CTGTCTACCTCCCCAATAATGACAGAATCTGCAGAAATAACCATCACCAC
CCAAACAGGTCCTCATGGGGCTACATCACAGGATACTTTTACCATGGACC
CATCAAATACAACCCCCCAGGCAGGGATCCACTCAGCTATGACTCATGGA
TTTTCACAATTGGATGTGACCACTCTTATGAGCAGAATTCCACAGGATGT
ATCATGGACAAGTCCTCCCTCTGTGGATAAAACCAGCTCCCCCTCTTCCT
TTCTGTCCTCACCTGCAATGACCACACCTTCCCTGATTTCTTCTACCTTA
CCAGAGGATAAGCTCTCCTCTCCTATGACTTCACTTCTCACCTCTGGCCT
AGTGAAGATTACAGACATATTACGTACACGCTTGGAACCTGTGACCAGCT
CACTTCCAAATTTCAGCAGCACCTCAGATAAGATACTGGCCACTTCTAAA
GACAGTAAAGACACAAAGGAAATTTTTCCTTCTATAAACACAGAAGAGAC
CAATGTGAAAGCCAACAACTCTGGACATGAATCCCATTCCCCTGCACTGG
CTGACTCAGAGACACCCAAAGCCACAACTCAAATGGTTATCACCACCACT
GTGGGAGATCCAGCTCCTTCCACATCAATGCCAGTGCATGGTTCCTCTGA
GACTACAAACATTAAGAGAGAGCCAACATATTTCTTGACTCCTAGACTGA
GAGAGACCAGTACCTCTCAGGAGTCCAGCTTTCCCACGGACACAAGTTTT
CTACTTTCCAAAGTCCCCACTGGTACTATTACTGAGGTCTCCAGTACAGG
GGTCAACTCTTCTAGCAAAATTTCCACCCCAGACCATGATAAGTCCACAG
TGCCACCTGACACCTTCACAGGAGAGATCCCCAGGGTCTTCACCTCCTCT

ATTAAGACAAAATCTGCAGAAATGACGATCACCACCCAAGCAAGTCCTCC
TGAGTCTGCATCGCACAGTACCCTTCCCTTGGACACATCAACCACACTTT
CCCAGGGAGGGACTCATTCAACTGTGACTCAGGGATTCCCATACTCAGAG
GTGACCACTCTCATGGGCATGGGTCCTGGGAATGTGTCATGGATGACAAC
TCCCCCTGTGGAAGAAACCAGCTCTGTGTCTTCCCTGATGTCTTCACCTG
CCATGACATCCCCTTCTCCTGTTTCCTCCACATCACCACAGAGCATCCCC
TCCTCTCCTCTTCCTGTGACTGCACTTCCTACTTCTGTTCTGGTGACAAC
CACAGATGTGTTGGGCACAACAAGCCCAGAGTCTGTAACCAGTTCACCTC
CAAATTTGAGCAGCATCACTCATGAGAGACCGGCCACTTACAAAGACACT
GCACACACAGAAGCCGCCATGCATCATTCCACAAACACCGCAGTGACCAA
TGTAGGGACTTCCGGGTCTGGACATAAATCACAATCCTCTGTCCTAGCTG
ACTCAGAGACATCGAAAGCCACACCTCTGATGAGTACCACCTCCACCCTG
GGGGACACAAGTGTTTCCACATCAACTCCTAATATCTCTCAGACTAACCA
AATTCAAACAGAGCCAACAGCATCCCTGAGCCCTAGACTGAGGGAGAGCA
GCACGTCTGAGAAGACCAGCTCAACAACAGAGACAAATACTGCCTTTTCT
TATGTGCCCACAGGTGCTATTACTCAGGCCTCCAGAACAGAAATCTCCTC
TAGCAGAACATCCATCTCAGACCTTGATCGGCCCACAATAGCACCCGACA
TCTCCACAGGAATGATCACCAGGCTCTTCACCTCCCCCATCATGACAAAA
TCTGCAGAAATGACCGTCACCACTCAAACAACTACTCCTGGGGCTACATC
ACAGGGTATCCTTCCCTGGGACACATCAACCACACTTTTCCAGGGAGGGA
CTCATTCAACCGTGTCTCAGGGATTCCCACACTCAGAGATAACCACTCTT
CGGAGCAGAACCCCTGGAGATGTGTCATGGATGACAACTCCCCCTGTGGA
AGAAACCAGCTCTGGGTTTTCCCTGATGTCACCTTCCATGACATCCCCTT
CTCCTGTTTCCTCCACATCACCAGAGAGCATCCCCTCCTCTCCTCTCCCT
GTGACTGCACTTCTTACTTCTGTTCTGGTGACAACCACAAATGTATTGGG
CACAACAAGCCCAGAGCCCGTAACGAGTTCACCTCCAAATTTAAGCAGCC
CCACACAGGAGAGACTGACCACTTACAAAGACACTGCGCACACAGAAGCC
ATGCATGCTTCCATGCATACAAACACTGCAGTGGCCAACGTGGGGACCTC
CATTTCTGGACATGAATCACAATCTTCTGTCCCAGCTGATTCACACACAT
CCAAAGCCACATCTCCAATGGGTATCACCTTCGCCATGGGGGATACAAGT
GTTTCTACATCAACTCCTGCCTTCTTTGAGACTAGAATTCAGACTGAATC
AACATCCTCTTTGATTCCTGGATTAAGGGACACCAGGACGTCTGAGGAGA
TCAACACTGTGACAGAGACCAGCACTGTCCTTTCAGAAGTGCCCACTACT
ACTACTACTGAGGTCTCCAGGACAGAAGTTATCACTTCCAGCAGAACAAC
CATCTCAGGGCCTGATCATTCCAAAATGTCACCCTACATCTCCACAGAAA
CCATCACCAGGCTCTCCACTTTTCCTTTTGTAACAGGATCCACAGAAATG
GCCATCACCAACCAAACAGGTCCTATAGGGACTATCTCACAGGCTACCCT
TACCCTGGACACATCAAGCACAGCTTCCTGGGAAGGGACTCACTCACCTG
TGACTCAGAGATTTCCACACTCAGAGGAGACCACTACTATGAGCAGAAGT
ACTAAGGGCGTGTCATGGCAAAGCCCTCCCTCTGTGGAAGAAACCAGTTC
TCCTTCTTCCCCAGTGCCTTTACCTGCAATAACCTCACATTCATCTCTTT
ATTCCGCAGTATCAGGAAGTAGCCCCACTTCTGCTCTCCCTGTGACTTCC
CTTCTCACCTCTGGCAGGAGGAAGACCATAGACATGTTGGACACACACTC
AGAACTTGTGACCAGCTCCTTACCAAGTGCAAGTAGCTTCTCAGGTGAGA
TACTCACTTCTGAAGCCTCCACAAATACAGAGACAATTCACTTTTCAGAG
AACACAGCAGAAACCAATATGGGGACCACCAATTCTATGCATAAACTACA
TTCCTCTGTCTCAATCCACTCCCAGCCATCCGGACACACACCTCCAAAGG

TTACTGGATCTATGATGGAGGACGCTATTGTTTCCACATCAACACCTGGT
TCTCCTGAGACTAAAAATGTTGACAGAGACTCAACATCCCCTCTGACTCC
TGAACTGAAAGAGGACAGCACCGCCCTGGTGATGAACTCAACTACAGAGT
CAAACACTGTTTTCTCCAGTGTGTCCCTGGATGCTGCTACTGAGGTCTCC
AGGGCAGAAGTCACCTACTATGATCCTACATTCATGCCAGCTTCTGCTCA
GTCAACAAAGTCCCCAGACATTTCACCTGAAGCCAGCAGCAGTCATTCTA
ACTCTCCTCCCTTGACAATATCTACACACAAGACCATCGCCACACAAACA
GGTCCTTCTGGGGTGACATCTCTTGGCCAACTGACCCTGGACACATCAAC
CATAGCCACCTCAGCAGGAACTCCATCAGCCAGAACTCAGGATTTTGTAG
ATTCAGAAACAACCAGTGTCATGAACAATGATCTCAATGATGTGTTGAAG
ACAAGCCCTTTCTCTGCAGAAGAAGCCAACTCTCTCTCTTCTCAGGCACC
TCTCCTTGTGACAACCTCACCTTCTCCTGTAACTTCCACATTGCAAGAGC
ACAGTACCTCCTCTCTTGTTTCTGTGACCTCAGTACCCACCCCTACACTG
GCGAAGATCACAGACATGGACACAAACTTAGAACCTGTGACTCGTTCACC
TCAAAATTTAAGGAACACCTTGGCCACTTCAGAAGCCACCACAGATACAC
ACACAATGCATCCTTCTATAAACACAGCAGTGGCCAATGTGGGGACCACC
AGTTCACCAAATGAATTCTATTTTACTGTCTCACCTGACTCAGACCCATA
TAAAGCCACATCCGCAGTAGTTATCACTTCCACCTCGGGGGACTCAATAG
TTTCCACATCAATGCCTAGATCCTCTGCGATGAAAAAGATTGAGTCTGAG
ACAACTTTCTCCCTGATATTTAGACTGAGGGAGACTAGCACCTCCCAGAA
AATTGGCTCATCCTCAGACACAAGCACGGTCTTTGACAAAGCATTCACTG
CTGCTACTACTGAGGTCTCCAGAACAGAACTCACCTCCTCTAGCAGAACA
TCCATCCAAGGCACTGAAAAGCCCACAATGTCACCGGACACCTCCACAAG
ATCTGTCACCATGCTTTCTACTTTTGCTGGCCTGACAAAATCCGAAGAAA
GGACCATTGCCACCCAAACAGGTCCTCATAGGGCGACATCACAGGGTACC
CTTACCTGGGACACATCAATCACAACCTCACAGGCAGGGACCCACTCAGC
TATGACTCATGGATTTTCACAATTAGATTTGTCCACTCTTACGAGTAGAG
TTCCTGAGTACATATCAGGGACAAGCCCACCCTCTGTGGAAAAAACCAGC
TCTTCCTCTTCCCTTCTGTCTTTACCAGCAATAACCTCACCGTCCCCTGT
ACCTACTACATTACCAGAAAGTAGGCCGTCTTCTCCTGTTCATCTGACTT
CACTCCCCACCTCTGGCCTAGTGAAGACCACAGATATGCTGGCATCTGTG
GCCAGTTTACCTCCAAACTTGGGCAGCACCTCACATAAGATACCGACTAC
TTCAGAAGACATTAAAGATACAGAGAAAATGTATCCTTCCACAAACATAG
CAGTAACCAATGTGGGGACCACCACTTCTGAAAAGGAATCTTATTCGTCT
GTCCCAGCCTACTCAGAACCACCCAAAGTCACCTCTCCAATGGTTACCTC
TTTCAACATAAGGGACACCATTGTTTCCACATCCATGCCTGGCTCCTCTG
AGATTACAAGGATTGAGATGGAGTCAACATTCTCCCTGGCTCATGGGCTG
AAGGGAACCAGCACCTCCCAGGACCCCATCGTATCCACAGAGAAAAGTGC
TGTCCTTCACAAGTTGACCACTGGTGCTACTGAGACCTCTAGGACAGAAG
TTGCCTCTTCTAGAAGAACATCCATTCCAGGCCCTGATCATTCCACAGAG
TCACCAGACATCTCCACTGAAGTGATCCCCAGCCTGCCTATCTCCCTTGG
CATTACAGAATCTTCAAATATGACCATCATCACTCGAACAGGTCCTCCTC
TTGGCTCTACATCACAGGGCACATTTACCTTGGACACACCAACTACATCC
TCCAGGGCAGGAACACACTCGATGGCGACTCAGGAATTTCCACACTCAGA
AATGACCACTGTCATGAACAAGGACCCTGAGATTCTATCATGGACAATCC
CTCCTTCTATAGAGAAAACCAGCTTCTCCTCTTCCCTGATGCCTTCACCA
GCCATGACTTCACCTCCTGTTTCCTCAACATTACCAAAGACCATTCACAC

CACTCCTTCTCCTATGACCTCACTGCTCACCCCTAGCCTAGTGATGACCA
CAGACACATTGGGCACAAGCCCAGAACCTACAACCAGTTCACCTCCAAAT
TTGAGCAGTACCTCACATGAGATACTGACAACAGATGAAGACACCACAGC
TATAGAAGCCATGCATCCTTCCACAAGCACAGCAGCGACTAATGTGGAAA
CCACCAGTTCTGGACATGGGTCACAATCCTCTGTCCTAGCTGACTCAGAA
AAAACCAAGGCCACAGCTCCAATGGATACCACCTCCACCATGGGGCATAC
AACTGTTTCCACATCAATGTCTGTTTCCTCTGAGACTACAAAAATTAAGA
GAGAGTCAACATATTCCTTGACTCCTGGACTGAGAGAGACCAGCATTTCC
CAAAATGCCAGCTTTTCCACTGACACAAGTATTGTTCTTTCAGAAGTCCC
CACTGGTACTACTGCTGAGGTCTCCAGGACAGAAGTCACCTCCTCTGGTA
GAACATCCATCCCTGGCCCTTCTCAGTCCACAGTTTTGCCAGAAATATCC
ACAAGAACAATGACAAGGCTCTTTGCCTCGCCCACCATGACAGAATCAGC
AGAAATGACCATCCCCACTCAAACAGGTCCTTCTGGGTCTACCTCACAGG
ATACCCTTACCTTGGACACATCCACCACAAAGTCCCAGGCAAAGACTCAT
TCAACTTTGACTCAGAGATTTCCACACTCAGAGATGACCACTCTCATGAG
CAGAGGTCCTGGAGATATGTCATGGCAAAGCTCTCCCTCTCTGGAAAATC
CCAGCTCTCTCCCTTCCCTGCTGTCTTTACCTGCCACAACCTCACCTCCT
CCCATTTCCTCCACATTACCAGTGACTATCTCCTCCTCTCCTCTTCCTGT
GACTTCACTTCTCACCTCTAGCCCGGTAACGACCACAGACATGTTACACA
CAAGCCCAGAACTTGTAACCAGTTCACCTCCAAAGCTGAGCCACACTTCA
GATGAGAGACTGACCACTGGCAAGGACACCACAAATACAGAAGCTGTGCA
TCCTTCCACAAACACAGCAGCGTCCAATGTGGAGATTCCCAGCTCTGGAC
ATGAATCCCCTTCCTCTGCCTTAGCTGACTCAGAGACATCCAAAGCCACA
TCACCAATGTTTATTACCTCCACCCAGGAGGATACAACTGTTGCCATATC
AACCCCTCACTTCTTGGAGACTAGCAGAATTCAGAAAGAGTCAATTTCCT
CCCTGAGCCCTAAATTGAGGGAGACAGGCAGTTCTGTGGAGACAAGCTCA
GCCATAGAGACAAGTGCTGTCCTTTCTGAAGTGTCCATTGGTGCTACTAC
TGAGATCTCCAGGACAGAAGTCACCTCCTCTAGCAGAACATCCATCTCTG
GTTCTGCTGAGTCCACAATGTTGCCAGAAATATCCACCACAAGAAAAATC
ATTAAGTTCCCTACTTCCCCCATCCTGGCAGAATCATCAGAAATGACCAT
CAAGACCCAAACAAGTCCTCCTGGGTCTACATCAGAGAGTACCTTTACAT
TAGACACATCAACCACTCCCTCCTTGGTAATAACCCATTCGACTATGACT
CAGAGATTGCCACACTCAGAGATAACCACTCTTGTGAGTAGAGGTGCTGG
GGATGTGCCACGGCCCAGCTCTCTCCCTGTGGAAGAAACAAGCCCTCCAT
CTTCCCAGCTGTCTTTATCTGCCATGATCTCACCTTCTCCTGTTTCTTCC
ACATTACCAGCAAGTAGCCACTCCTCTTCTGCTTCTGTGACTTCACTTCT
CACACCAGGCCAAGTGAAGACTACTGAGGTGTTGGACGCAAGTGCAGAAC
CTGAAACCAGTTCACCTCCAAGTTTGAGCAGCACCTCAGTTGAAATACTG
GCCACCTCTGAAGTCACCACAGATACGGAGAAAATTCATCCTTTCTCAAA
CACGGCAGTAACCAAAGTTGGAACTTCCAGTTCTGGACATGAATCCCCTT
CCTCTGTCCTACCTGACTCAGAGACAACCAAAGCCACATCGGCAATGGGT
ACCATCTCCATTATGGGGGATACAAGTGTTTCTACATTAACTCCTGCCTT
ATCTAACACTAGGAAAATTCAGTCAGAGCCAGCTTCCTCACTGACCACCA
GATTGAGGGAGACCAGCACCTCTGAAGAGACCAGCTTAGCCACAGAAGCA
AACACTGTTCTTTCTAAAGTGTCCACTGGTGCTACTACTGAGGTCTCCAG
GACAGAAGCCATCTCCTTTAGCAGAACATCCATGTCAGGCCCTGAGCAGT
CCACAATGTCACAAGACATCTCCATAGGAACCATCCCCAGGATTTCTGCC

TCCTCTGTCCTGACAGAATCTGCAAAAATGACCATCACAACCCAAACAGG
TCCTTCGGAGTCTACACTAGAAAGTACCCTTAATTTGAACACAGCAACCA
CACCCTCTTGGGTGGAAACCCACTCTATAGTAATTCAGGGATTTCCACAC
CCAGAGATGACCACTTCCATGGGCAGAGGTCCTGGAGGTGTGTCATGGCC
TAGCCCTCCCTTTGTGAAAGAAACCAGCCCTCCATCCTCCCCGCTGTCTT
TACCTGCCGTGACCTCACCTCATCCTGTTTCCACCACATTCCTAGCACAT
ATCCCCCCCTCTCCCCTTCCTGTGACTTCACTTCTCACCTCTGGCCCGGC
GACAACCACAGATATCTTGGGTACAAGCACAGAACCTGGAACCAGTTCAT
CTTCAAGTTTGAGCACCACCTCCCATGAGAGACTGACCACTTACAAAGAC
ACTGCACATACAGAAGCCGTGCATCCTTCCACAAACACAGGAGGGACCAA
TGTGGCAACCACCAGCTCTGGATATAAATCACAGTCCTCTGTCCTAGCTG
ACTCATCTCCAATGTGTACCACCTCCACCATGGGGGATACAAGTGTTCTC
ACATCAACTCCTGCCTTCCTTGAGACTAGGAGGATTCAGACAGAGCTAGC
TTCCTCCCTGACCCCTGGATTGAGGGAGTCCAGCGGCTCTGAAGGGACCA
GCTCAGGCACCAAGATGAGCACTGTCCTCTCTAAAGTGCCCACTGGTGCT
ACTACTGAGATCTCCAAGGAAGACGTCACCTCCATCCCAGGTCCCGCTCA
ATCCACAATATCACCAGACATCTCCACAAGAACCGTCAGCTGGTTCTCTA
CATCCCCTGTCATGACAGAATCAGCAGAAATAACCATGAACACCCATACA
AGTCCTTTAGGGGCCACAACACAAGGCACCAGTACTTTGGACACGTCAAG
CACAACCTCTTTGACAATGACACACTCAACTATATCTCAAGGATTTTCAC
ACTCACAGATGAGCACTCTTATGAGGAGGGGTCCTGAGGATGTATCATGG
ATGAGCCCTCCCCTTCTGGAAAAAACTAGACCTTCCTTTTCTCTGATGTC
TTCACCAGCCACAACTTCACCTTCTCCTGTTTCCTCCACATTACCAGAGA
GCATCTCTTCCTCTCCTCTTCCTGTGACTTCACTCCTCACGTCTGGCTTG
GCAAAAACTACAGATATGTTGCACAAAAGCTCAGAACCTGTAACCAACTC
ACCTGCAAATTTGAGCAGCACCTCAGTTGAAATACTGGCCACCTCTGAAG
TCACCACAGATACAGAGAAAACTCATCCTTCTTCAAACAGAACAGTGACC
GATGTGGGGACCTCCAGTTCTGGACATGAATCCACTTCCTTTGTCCTAGC
TGACTCACAGACATCCAAAGTCACATCTCCAATGGTTATTACCTCCACCA
TGGAGGATACGAGTGTCTCCACATCAACTCCTGGCTTTTTTGAGACTAGC
AGAATTCAGACAGAACCAACATCCTCCCTGACCCTTGGACTGAGAAAGAC
CAGCAGCTCTGAGGGGACCAGCTTAGCCACAGAGATGAGCACTGTCCTTT
CTGGAGTGCCCACTGGTGCCACTGCTGAAGTCTCCAGGACAGAAGTCACC
TCCTCTAGCAGAACATCCATCTCAGGCTTTGCTCAGCTCACAGTGTCACC
AGAGACTTCCACAGAAACCATCACCAGACTCCCTACCTCCAGCATAATGA
CAGAATCAGCAGAAATGATGATCAAGACACAAACAGATCCTCCTGGGTCT
ACACCAGAGAGTACTCATACTGTGGACATATCAACAACACCCAACTGGGT
AGAAACCCACTCGACTGTGACTCAGAGATTTTCACACTCAGAGATGACCA
CTCTTGTGAGCAGAAGCCCTGGTGATATGTTATGGCCTAGTCAATCCTCT
GTGGAAGAAACCAGCTCTGCCTCTTCCCTGCTGTCTCTGCCTGCCACGAC
CTCACCTTCTCCTGTTTCCTCTACATTAGTAGAGGATTTCCCTTCCGCTT
CTCTTCCTGTGACTTCTCTTCTCAACCCTGGCCTGGTGATAACCACAGAC
AGGATGGGCATAAGCAGAGAACCTGGAACCAGTTCCACTTCAAATTTGAG
CAGCACCTCCCATGAGAGACTGACCACTTTGGAAGACACTGTAGATACAG
AAGACATGCAGCCTTCCACACACACAGCAGTGACCAACGTGAGGACCTCC
ATTTCTGGACATGAATCACAATCTTCTGTCCTATCTGACTCAGAGACACC
CAAAGCCACATCTCCAATGGGTACCACCTACACCATGGGGGAAACGAGTG

TTTCCATATCCACTTCTGACTTCTTTGAGACCAGCAGAATTCAGATAGAA
CCAACATCCTCCCTGACTTCTGGATTGAGGGAGACCAGCAGCTCTGAGAG
GATCAGCTCAGCCACAGAGGGAAGCACTGTCCTTTCTGAAGTGCCCAGTG
GTGCTACCACTGAGGTCTCCAGGACAGAAGTGATATCCTCTAGGGGAACA
TCCATGTCAGGGCCTGATCAGTTCACCATATCACCAGACATCTCTACTGA
AGCGATCACCAGGCTTTCTACTTCCCCCATTATGACAGAATCAGCAGAAA
GTGCCATCACTATTGAGACAGGTTCTCCTGGGGCTACATCAGAGGGTACC
CTCACCTTGGACACCTCAACAACAACCTTTTGGTCAGGGACCCACTCAAC
TGCATCTCCAGGATTTTCACACTCAGAGATGACCACTCTTATGAGTAGAA
CTCCTGGAGATGTGCCATGGCCGAGCCTTCCCTCTGTGGAAGAAGCCAGC
TCTGTCTCTTCCTCACTGTCTTCACCTGCCATGACCTCAACTTCTTTTTT
CTCCACATTACCAGAGAGCATCTCCTCCTCTCCTCATCCTGTGACTGCAC
TTCTCACCCTTGGCCCAGTGAAGACCACAGACATGTTGCGCACAAGCTCA
GAACCTGAAACCAGTTCACCTCCAAATTTGAGCAGCACCTCAGCTGAAAT
ATTAGCCACGTCTGAAGTCACCAAAGATAGAGAGAAAATTCATCCCTCCT
CAAACACACCTGTAGTCAATGTAGGGACTGTGATTTATAAACATCTATCC
CCTTCCTCTGTTTTGGCTGACTTAGTGACAACAAAACCCACATCTCCAAT
GGCTACCACCTCCACTCTGGGGAATACAAGTGTTTCCACATCAACTCCTG
CCTTCCCAGAAACTATGATGACACAGCCAACTTCCTCCCTGACTTCTGGA
TTAAGGGAGATCAGTACCTCTCAAGAGACCAGCTCAGCAACAGAGAGAAG
TGCTTCTCTTTCTGGAATGCCCACTGGTGCTACTACTAAGGTCTCCAGAA
CAGAAGCCCTCTCCTTAGGCAGAACATCCACCCCAGGTCCTGCTCAATCC
ACAATATCACCAGAAATCTCCACGGAAACCATCACTAGAATTTCTACTCC
CCTCACCACGACAGGATCAGCAGAAATGACCATCACCCCCAAAACAGGTC
ATTCTGGGGCATCCTCACAAGGTACCTTTACCTTGGACACATCAAGCAGA
GCCTCCTGGCCAGGAACTCACTCAGCTGCAACTCACAGATCTCCACACTC
AGGGATGACCACTCCTATGAGCAGAGGTCCTGAGGATGTGTCATGGCCAA
GCCGCCCATCAGTGGAAAAAACTAGCCCTCCATCTTCCCTGGTGTCTTTA
TCTGCAGTAACCTCACCTTCGCCACTTTATTCCACACCATCTGAGAGTAG
CCACTCATCTCCTCTCCGGGTGACTTCTCTTTTCACCCCTGTCATGATGA
AGACCACAGACATGTTGGACACAAGCTTGGAACCTGTGACCACTTCACCT
CCCAGTATGAATATCACCTCAGATGAGAGTCTGGCCACTTCTAAAGCCAC
CATGGAGACAGAGGCAATTCAGCTTTCAGAAAACACAGCTGTGACTCAGA
TGGGCACCATCAGCGCTAGACAAGAATTCTATTCCTCTTATCCAGGCCTC
CCAGAGCCATCCAAAGTGACATCTCCAGTGGTCACCTCTTCCACCATAAA
AGACATTGTTTCTACAACCATACCTGCTTCCTCTGAGATAACAAGAATTG
AGATGGAGTCAACATCCACCCTGACCCCCACACCAAGGGAGACCAGCACC
TCCCAGGAGATCCACTCAGCCACAAAGCCAAGCACTGTTCCTTACAAGGC
ACTCACTAGTGCCACGATTGAGGACTCCATGACACAAGTCATGTCCTCTA
GCAGAGGACCTAGCCCTGATCAGTCCACAATGTCACAAGACATATCCACT
GAAGTGATCACCAGGCTCTCTACCTCCCCCATCAAGACAGAATCTACAGA
AATGACCATTACCACCCAAACAGGTTCTCCTGGGGCTACATCAAGGGGTA
CCCTTACCTTGGACACTTCAACAACTTTTATGTCAGGGACCCACTCAACT
GCATCTCAAGGATTTTCACACTCACAGATGACCGCTCTTATGAGTAGAAC
TCCTGGAGATGTGCCATGGCTAAGCCATCCCTCTGTGGAAGAAGCCAGCT
CTGCCTCTTTCTCACTGTCTTCACCTGTCATGACCTCATCTTCTCCCGTT
TCTTCCACATTACCAGACAGCATCCACTCTTCTTCGCTTCCTGTGACATC

ACTTCTCACCTCAGGGCTGGTGAAGACCACAGAGCTGTTGGGCACAAGCT
CAGAACCTGAAACCAGTTCACCCCCAAATTTGAGCAGCACCTCAGCTGAA
ATACTGGCCATCACTGAAGTCACTACAGATACAGAGAAACTGGAGATGAC
CAATGTGGTAACCTCAGGTTATACACATGAATCTCCTTCCTCTGTCCTAG
CTGACTCAGTGACAACAAAGGCCACATCTTCAATGGGTATCACCTACCCC
ACAGGAGATACAAATGTTCTCACATCAACCCCTGCCTTCTCTGACACCAG
TAGGATTCAAACAAAGTCAAAGCTCTCACTGACTCCTGGGTTGATGGAGA
CCAGCATCTCTGAAGAGACCAGCTCTGCCACAGAAAAAAGCACTGTCCTT
TCTAGTGTGCCCACTGGTGCTACTACTGAGGTCTCCAGGACAGAAGCCAT
CTCTTCTAGCAGAACATCCATCCCAGGCCCTGCTCAATCCACAATGTCAT
CAGACACCTCCATGGAAACCATCACTAGAATTTCTACCCCCCTCACAAGG
AAAGAATCAACAGACATGGCCATCACCCCCAAAACAGGTCCTTCTGGGGC
TACCTCGCAGGGTACCTTTACCTTGGACTCATCAAGCACAGCCTCCTGGC
CAGGAACTCACTCAGCTACAACTCAGAGATTTCCACAGTCAGTGGTGACA
ACTCCTATGAGCAGAGGTCCTGAGGATGTGTCATGGCCAAGCCCGCTGTC
TGTGGAAAAAAACAGCCCTCCATCTTCCCTGGTATCTTCATCTTCAGTAA
CCTCACCTTCGCCACTTTATTCCACACCATCTGGGAGTAGCCACTCCTCT
CCTGTCCCTGTCACTTCTCTTTTCACCTCTATCATGATGAAGGCCACAGA
CATGTTGGATGCAAGTTTGGAACCTGAGACCACTTCAGCTCCCAATATGA
ATATCACCTCAGATGAGAGTCTGGCCGCTTCTAAAGCCACCACGGAGACA
GAGGCAATTCACGTTTTTGAAAATACAGCAGCGTCCCATGTGGAAACCAC
CAGTGCTACAGAGGAACTCTATTCCTCTTCCCCAGGCTTCTCAGAGCCAA
CAAAAGTGATATCTCCAGTGGTCACCTCTTCCTCTATAAGAGACAACATG
GTTTCCACAACAATGCCTGGCTCCTCTGGCATTACAAGGATTGAGATAGA
GTCAATGTCATCTCTGACCCCTGGACTGAGGGAGACCAGAACCTCCCAGG
ACATCACCTCATCCACAGAGACAAGCACTGTCCTTTACAAGATGCCCTCT
GGTGCCACTCCTGAGGTCTCCAGGACAGAAGTTATGCCCTCTAGCAGAAC
ATCCATTCCTGGCCCTGCTCAGTCCACAATGTCACTAGACATCTCCGATG
AAGTTGTCACCAGGCTGTCTACCTCTCCCATCATGACAGAATCTGCAGAA
ATAACCATCACCACCCAAACAGGTTATTCTCTGGCTACATCCCAGGTTAC
CCTTCCCTTGGGCACCTCAATGACCTTTTTGTCAGGGACCCACTCAACTA
TGTCTCAAGGACTTTCACACTCAGAGATGACCAATCTTATGAGCAGGGGT
CCTGAAAGTCTGTCATGGACGAGCCCTCGCTTTGTGGAAACAACTAGATC
TTCCTCTTCTCTGACATCATTACCTCTCACGACCTCACTTTCTCCTGTGT
CCTCCACATTACTAGACAGTAGCCCCTCCTCTCCTCTTCCTGTGACTTCA
CTTATCCTCCCAGGCCTGGTGAAGACTACAGAAGTGTTGGATACAAGCTC
AGAGCCTAAAACCAGTTCATCTCCAAATTTGAGCAGCACCTCAGTTGAAA
TACCGGCCACCTCTGAAATCATGACAGATACAGAGAAAATTCATCCTTCC
TCAAACACAGCGGTGGCCAAAGTGAGGACCTCCAGTTCTGTTCATGAATC
TCATTCCTCTGTCCTAGCTGACTCAGAAACAACCATAACCATACCTTCAA
TGGGTATCACCTCCGCTGTGGACGATACCACTGTTTTCACATCAAATCCT
GCCTTCTCTGAGACTAGGAGGATTCCGACAGAGCCAACATTCTCATTGAC
TCCTGGATTCAGGGAGACTAGCACCTCTGAAGAGACCACCTCAATCACAG
AAACAAGTGCAGTCCTTTATGGAGTGCCCACTAGTGCTACTACTGAAGTC
TCCATGACAGAAATCATGTCCTCTAATAGAATACACATCCCTGACTCTGA
TCAGTCCACGATGTCTCCAGACATCATCACTGAAGTGATCACCAGGCTCT
CTTCCTCATCCATGATGTCAGAATCAACACAAATGACCATCACCACCCAA

AAAAGTTCTCCTGGGGCTACAGCACAGAGTACTCTTACCTTGGCCACAAC
AACAGCCCCCTTGGCAAGGACCCACTCAACTGTTCCTCCTAGATTTTTAC
ACTCAGAGATGACAACTCTTATGAGTAGGAGTCCTGAAAATCCATCATGG
AAGAGCTCTCTCTTTGTGGAAAAAACTAGCTCTTCATCTTCTCTGTTGTC
CTTACCTGTCACGACCTCACCTTCTGTTTCTTCCACATTACCGCAGAGTA
TCCCTTCCTCCTCTTTTTCTGTGACTTCACTCCTCACCCCAGGCATGGTG
AAGACTACAGACACAAGCACAGAACCTGGAACCAGTTTATCTCCAAATCT
GAGTGGCACCTCAGTTGAAATACTGGCTGCCTCTGAAGTCACCACAGATA
CAGAGAAAATTCATCCTTCTTCAAGCATGGCAGTGACCAATGTGGGAACC
ACCAGTTCTGGACATGAACTATATTCCTCTGTTTCAATCCACTCGGAGCC
ATCCAAGGCTACATACCCAGTGGGTACTCCCTCTTCCATGGCTGAAACCT
CTATTTCCACATCAATGCCTGCTAATTTTGAGACCACAGGATTTGAGGCT
GAGCCATTTTCTCATTTGACTTCTGGATTTAGGAAGACAAACATGTCCCT
GGACACCAGCTCAGTCACACCAACAAATACACCTTCTTCTCCTGGGTCCA
CTCACCTTTTACAGAGTTCCAAGACTGATTTCACCTCTTCTGCAAAAACA
TCATCCCCAGACTGGCCTCCAGCCTCACAGTATACTGAAATTCCAGTGGA
CATAATCACCCCCTTTAATGCTTCTCCATCTATTACGGAGTCCACTGGGA
TAACCTCCTTCCCAGAATCCAGGTTTACTATGTCTGTAACAGAAAGTACT
CATCATCTGAGTACAGATTTGCTGCCTTCAGCTGAGACTATTTCCACTGG
CACAGTGATGCCTTCTCTATCAGAGGCCATGACTTCATTTGCCACCACTG
GAGTTCCACGAGCCATCTCAGGTTCAGGTAGTCCATTCTCTAGGACAGAG
TCAGGCCCTGGGGATGCTACTCTGTCCACCATTGCAGAGAGCCTGCCTTC
ATCCACTCCTGTGCCATTCTCCTCTTCAACCTTCACTACCACTGATTCTT
CAACCATCCCAGCCCTCCATGAGATAACTTCCTCTTCAGCTACCCCATAT
AGAGTGGACACCAGTCTTGGGACAGAGAGCAGCACTACTGAAGGACGCTT
GGTTATGGTCAGTACTTTGGACACTTCAAGCCAACCAGGCAGGACATCTT
CATCACCCATTTTGGATACCAGAATGACAGAGAGCGTTGAGCTGGGAACA
GTGACAAGTGCTTATCAAGTTCCTTCACTCTCAACACGGTTGACAAgtaa
ggacccacagcccctacaatcccattattggggctcataggaaatgacc
ccttcctaagaagcaaaaaaagagttaactagtttattcctgtaccagaa
attctcttagcaaacatgtgtttatctaatttcactcctaggcactgggt
taaggaattggagaaacaaaaataagcaaataattgaatcattacagtat
cgtgataggtgctctaatattactactagtaatgatgatgatgacaataa
gaatgacaacagaaaatatttataacagtacttactctgtttcaagtgca
gttttattaagctatttgatcctcaattgtaacactaattgaagtagaga
ttattagaagcctattttggagatgaagacatttaggcacagggagatta
agtaactttcttaagtccatatagctagaaagcaatagaactaggataag
aactcaaattgtgtgtcactaggaactcatgctcctaactgctgggctaa
acaggagtggtcagagaggtaacagaaaaggcaagaagttctggcatcac
agaagccacaggaggggagggttttaggaaggagaaatggtaagttctgt
taaataccacagaattgccaagttgaaatgaggtgagaagtgctttggca
acatgggggttctcagggattctaatgaagggagcatcagtgaggtatag
ggcattagaagaacagctaaaatactacgtagacgtaagggagggtttct
ccctttgagttgagcaagagacaaaagggataagtgaaaggaaaaagtga
attaatgaaaatagagggaacaaagtacctaagaaggtagaagacaccca
gagcctagaaggaggaaacactacttccaagaggtcagtagataaaggaa
ggaacttcttggggattccattcctctgaaagggttgagcttggcaccaa

ctttgttctggacacaaatccaaagaggccctccaacgactcgagtttc
tgcctcactggacaatatctccaggagaatgatttgctccctctccttct
tcacattattttgtaattcacactaaatttagacacattggtggctattg
acccatatgttactatgtgactcagtagttgagtcacgggtgttcagtaa
gcaagtgatgaatggcatggcgagaagggagaagtgtagttggatggata
aaaggaagaatggagagaagagtgaatggaaggaagcaaagatgaagcgg
aggaaggatagatgcacagaaggaaggatgaaaagaaagaaagatgatgg
aagacaggattgaaggggatatagattgaaggaaagaaaggtagaaggat
gaaatgaagtaaagattgaagaaaagatggatggaaagaagaaaggaggg
tgcacaaaaaatctcacacttcaccacatatgattcatccatataagaaa
aaaccacttgtaccctcaaagctattgaaatacaaacttttaaattaaaa
ttttaaaaagcaagagaaaggaaagaagggaggaaagacaaaaggaagaa
tgggtgatagaaggaaagaataaaaggaagaaaaaaatggaagaatagatg
atcagatctagggatgaatgaaaggaaggatggacaaatctataggtagg
tggatggatctatggacaggtgtggccacttatggcacatagtcccagct
ccagttcatactgatggacttgaggagtgtttgtggccaatgaagtggat
ccatttagacagtgctcttcttctgaatgagatgagttaccccagttttt
ctccccaccttcatcttcagGAACTGATGGCATTATGGAACACATCACAA
AAATACCCAATGAAGCAGCACACAGAGGTACCATAAGACCAGTCAAAGGC
CCTCAGACATCCACTTCGCCTGCCAGTCCTAAAGgtaggtttaactttgc
ttacctcccagtaatgccactcgtgaccatatttcctcctccagagagac
aaaatgtttgtattctttagagagagagaattgtgtgtggttgtcataggtt
tccctgtctgaactgagtctttatctaatggttaccaggcagatgttacc
acttctctttctcctcatggcatgctgagtgagttttgtccaacatcaaa
tattcacaaatttgtccatattaaccaaattttaaaaatgctcattaaaa
acttactatgagctgggcgcagtggctcatgcctgtaatcccaatacttt
gggaggctgaggtgggtggatcacctgaggtcaaaaattcgagaccagtc
tgaccaaaatggtgaaactccatctctactgaaaatataaaaattagccg
ggcatggtggcacacacctgtaatcacagctactcaggaggctgaggcaa
gagagtcacttgaacccaggaggtagaggctgcagtgagctgagattgtg
ccaatgcactccagcctgggtggcagagcaagactccagctcaaaaataa
ataaataaattatatatatatatatatatttatttaaataaaatatact
atatatatatgtatatatatatgtatgtatatatatatatgtatgtatat
atatatatatatatatatatatatatagagagagagagagagagagag
agagagagagacagagtatgtctgaaaatgcatcccaatagttctagcaa
atgtaggaaaaggaagtataaattaacagcctttatgtatgccctggttg
aaaaacagacataactctcttgtaagagaaacttcacaaaaatatctagg
attatatctcccatgatgaaaaatttggaactgtacatttttttaactg
tcacttaaataaagaagatgggtgagctcctgggttatttttgtttgtca
tcactggagagctaaatcacaatatgtaagtacaatgggggaataaaaa
cacgatataaatgtaagatggtttatgacatcactgttatatgttttctt
tgtcacaatatttagtaattcctccttctgttttgaataaagtctgaaac
aaaattttcactttaaaaggcagtaatatgaaatacataaataaataaaa
atctgggtttctcaactttgacactattgtcatttggggcagatacttct
ctgcgatgggggctgtgctgtgcgttgtaggatgtttaccagcctctctg
gcctctgttctagatgccataccttctgcccacacacagttgtgacaacc
aaaaatatctccagacattgttaagtgtcccctggaagcaaaactgcccc

tggttgagaaccactgaactggagaatgcatcctaagatccatcttacta
gatgggcgttttcacctcacttcattctctttccattctggtccccacaa
gatccagaagttcctttagtgaattaatataatgcaatggttaataatat
gggtttaggatttagatacgatgatgttcaagtttttgccctgtcaatag
ctgtgtgtgaccttggtaaattgtggtctctggatttctctttctctcct
gtaaaatgacaagaattatacaatgtgcattttattttattttatctcac
tttatttatttatttatttgaggtggagtcttgctctgtcacccaagctg
gagtgcagaggtgcaatctcggctccctgcaacctccatcccctgagttt
gagtcattctcctgcctcagcctcccgagtagctgggattacagatgcac
gccactaagcctggctaattttttgtatttttagtagagatggggtttgac
catgttgaccaggctggtctagaactcctgacctcaggtgatccgcccac
ctcgtcctcccaaaatgctgggattacaggcatgagccaccgcgcctggc
cagaatgtgccatttaatgttcttgtgaggtttaaaaagacagtgcatgt
caagtgctttgcaattgctcctcctcctccttcccatcaacataatcacc
atcatggtcaccattattgttagatacacatgaagcacttgagagtgcat
tctcagacatattgttttagaggatatccagcaacacagggacactgct
ccataatcagagtcaaaatccctttcatttaccccctttctctaccaag
ctccagtaggctttgtgggctttatagagaagcctccagttagttcttcc
tcttccctaggaaccatttctaaagcctggtcagcagctgctgtctcat
caatgctactggaatcttgttcaaaggtattccagtcaccttggtcagct
ctggtagtgtctagtgtggttggtggagaattctatttagcaaatgttta
ttttgtagtagaattattagtagtgacagtagtaatggtaagtagtagtg
gtagtaacaatagcagcaatagcactagtaagtagtagtaactgtagcaa
taaccatagcaataagcagtaatactagtagtaatagtagtgataatggc
agtaatggtatgtagagttgtgttggtagtaacagtagtagcagtaggta
gtagcagtggtagtagtgacagtagaaatcataagtaatagtgctagcaa
caatagtattaatagtagtaaccatagtaataacatagtaataaccatag
caataggcagtaatattagtagtaacagtagtaataacagcagtaatgac
agagcactaccattactgctgttactctactgtaggtagagtagtaatat
taagtggtagtgatagtaataatagtaatagcaacagcagtagtaatagt
aagtagcagtaaccatagtaataaccatagtgataggtagtaatagttgt
ggtaacagtagtaataactgcagtaatggtaggtagagtggtggtagtag
taacagtagtagtagcagtggtagcagtaacagtagcaatagtaatagtg
gtagtaacagtagtagcagtactagtagtagtaatcatagtaataaccat
tgcaataggtagtagtaacagtagcaataacagcagtaatggtaggtgga
gaagtgatagtaataacagtagcaatagcaggtcatagtcgtggtaatag
tagtaatgtcttatcgtgacccaggaggcactgtgcttggcgccttttta
ccaacactttgagatggccattgtacttatccccactttatagacgggaa
aatggaggtccagcaatatttttaacttaaagagccacccatctcttta
gagaaagagccagaatcccaggcagggctatcttattccagagcccaagc
tctcaaacacatgatacacaatacttaatctctctcaagtcagaggagat
ccacttaagtatacatccatccacatattcattcattcaatcattcaaca
aatattagttgagcacttaccgtatgccaaacagtcaaacgtgaatagct
gttacaaatgagactgtgaaggatggtacaacgcagattcagacagtgtg
ataaggaaatattgagaagcaaagatgagttctggagtgaatttgtaaag
gtggatgtgggcttggatttcaataatggcagaacttaaggaatctgatg
agaagtgggcacttcaggcagagagaagagcttgaacaaggctcagaggc

tgacagtgcaggaaacacatgggaagagggaatagagtagcggtcaagaa
ttcacagaggagttataggtgaagatgcaaccaagttacagaccaaggta
agataggggaataccaatcacaatctcttttcccattccagaagcatccc
agacacatcctagtaaccgagagacatttctctccctttcctcctgtgga
gaataaataagctattgcaagtccagtaagtgtaatcattttgttcaaat
tgtgtgcccattccccaatttacagGACTACACACAGGAGGGACAAAAAG
AATGGAGACCACCACCACAGCTCTGAAGACCACCACCACAGCTCTGAAGA
CCACTTCCAGAGCCACCTTGACCACCAGTGTCTATACTCCCACTTTGGGA
ACACTGACTCCCCTCAATGCATCAATGCAAATGGCCAGCACAATCCCCAC
AGAAATGATGATCACAACCCCATATGTTTTCCCTGATGTTCCAGAAACGA
CATCCTCATTGGCTACCAGCCTGGGAGCAGAAACCAGCACAGCTCTTCCC
AGGACAACCCCATCTGTTTTCAATAGAGAATCAGAGACCACAGCCTCACT
GGTCTCTCGTTCTGGGGCAGAGAGAAGTCCGGTTATTCAAACTCTAGATG
TTTCTTCTAGTGAGCCAGATACAACAGCTTCATGGGTTATCCATCCTGCA
GAGACCATCCCAACTGTTTCCAAGACAACCCCCAATTTTTTCCACAGTGA
ATTAGACACTGTATCTTCCACAGCCACCAGTCATGGGGCAGACGTCAGCT
CAGCCATTCCAACAAATATCTCACCTAGTGAACTAGATGCACTGACCCCA
CTGGTCACTATTTCGGGGACAGATACTAGTACAACATTCCCAACACTGAC
TAAGTCCCCACATGAAACAGAGACAAGAACCACATGGCTCACTCATCCTG
CAGAGACCAGCTCAACTATTCCCAGAACAATCCCCAATTTTTCTCATCAT
GAATCAGATGCCACACCTTCAATAGCCACCAGTCCTGGGGCAGAAACCAG
TTCAGCTATTCCAATTATGACTGTCTCACCTGGTGCAGAAGATCTGGTGA
CCTCACAGGTCACTAGTTCTGGGACAGACAGAAATATGACTATTCCAACT
TTGACTCTTTCTCCTGGTGAACCAAAGACGATAGCCTCATTAGTCACCCA
TCCTGAAGCACAGACAAGTTCGGCCATTCCAACTTCAACTATCTCGCCTG
CTGTATCACGGTTGGTGACCTCAATGGTCACCAGTTTGGCGGCAAAGACA
AGTACAACTAATCGAGCTCTGACAAACTCCCCTGGTGAACCAGCTACAAC
AGTTTCATTGGTCACGCATCCTGCACAGACCAGCCCAACAGTTCCCTGGA
CAACTTCCATTTTTTTCCATAGTAAATCAGACACCACACCTTCAATGACC
ACCAGTCATGGGGCAGAATCCAGTTCAGCTGTTCCAACTCCAACTGTTTC
AACTGAGGTACCAGGAGTAGTGACCCCTTTGGTCACCAGTTCTAGGGCAG
TGATCAGTACAACTATTCCAATTCTGACTCTTTCTCCTGGTGAACCAGAG
ACCACACCTTCAATGGCCACCAGTCATGGGGAAGAAGCCAGTTCTGCTAT
TCCAACTCCAACTGTTTCACCTGGGGTACCAGGAGTGGTGACCTCTCTGG
TCACTAGTTCTAGGGCAGTGACTAGTACAACTATTCCAATTCTGACTTTT
TCTCTTGGTGAACCAGAGACCACACCTTCAATGGCCACCAGTCATGGGAC
AGAAGCTGGCTCAGCTGTTCCAACTGTTTTACCTGAGGTACCAGGAATGG
TGACCTCTCTGGTTGCTAGTTCTAGGGCAGTAACCAGTACAACTCTTCCA
ACTCTGACTCTTTCTCCTGGTGAACCAGAGACCACACCTTCAATGGCCAC
CAGTCATGGGGCAGAAGCCAGCTCAACTGTTCCAACTGTTTCACCTGAGG
TACCAGGAGTGGTGACCTCTCTGGTCACTAGTTCTAGTGGAGTAAACAGT
ACAAGTATTCCAACTCTGATTCTTTCTCCTGGTGAACTAGAAACCACACC
TTCAATGGCCACCAGTCATGGGGCAGAAGCCAGCTCAGCTGTTCCAACTC
CAACTGTTTCACCTGGGGTATCAGGAGTGGTGACCCCTCTGGTCACTAGT
TCCAGGGCAGTGACCAGTACAACTATTCCAATTCTAACTCTTTCTTCTAG
TGAGCCAGAGACCACACCTTCAATGGCCACCAGTCATGGGGTAGAAGCCA
GCTCAGCTGTTCTAACTGTTTCACCTGAGGTACCAGGAATGGTGACCTCT

CTGGTCACTAGTTCTAGAGCAGTAACCAGTACAACTATTCCAACTCTGAC
TATTTCTTCTGATGAACCAGAGACCACAACTTCATTGGTCACCCATTCTG
AGGCAAAGATGATTTCAGCCATTCCAACTTTAGCTGTCTCCCCTACTGTA
CAAGGGCTGGTGACTTCACTGGTCACTAGTTCTGGGTCAGAGACCAGTGC
GTTTTCAAATCTAACTGTTGCCTCAAGTCAACCAGAGACCATAGACTCAT
GGGTCGCTCATCCTGGGACAGAAGCAAGTTCTGTTGTTCCAACTTTGACT
GTCTCCACTGGTGAGCCGTTTACAAATATCTCATTGGTCACCCATCCTGC
AGAGAGTAGCTCAACTCTTCCCAGGACAACCTCAAGGTTTTCCCACAGTG
AATTAGACACTATGCCTTCTACAGTCACCAGTCCTGAGGCAGAATCCAGC
TCAGCCATTTCAACAACTATTTCACCTGGTATACCAGGTGTGCTGACATC
ACTGGTCACTAGCTCTGGGAGAGACATCAGTGCAACTTTTCCAACAGTGC
CTGAGTCCCCACATGAATCAGAGGCAACAGCCTCATGGGTTACTCATCCT
GCAGTCACCAGCACAACAGTTCCCAGGACAACCCCTAATTATTCTCATAG
TGAACCAGACACCACACCATCAATAGCCACCAGTCCTGGGGCAGAAGCCA
CTTCAGATTTTCCAACAATAACTGTCTCACCTGATGTACCAGATATGGTA
ACCTCACAGGTCACTAGTTCTGGGACAGACACCAGTATAACTATTCCAAC
TCTGACTCTTTCTTCTGGTGAGCCAGAGACCACAACCTCATTTATCACCT
ATTCTGAGACACACACAAGTTCAGCCATTCCAACTCTCCCTGTCTCCCCT
GGTGCATCAAAGATGCTGACCTCACTGGTCATCAGTTCTGGGACAGACAG
CACTACAACTTTCCCAACACTGACGGAGACCCCATATGAACCAGAGACAA
CAGCCATACAGCTCATTCATCCTGCAGAGACCAACACAATGGTTCCCAGG
ACAACTCCCAAGTTTTCCCATAGTAAGTCAGACACCACACTCCCAGTAGC
CATCACCAGTCCTGGGCCAGAAGCCAGTTCAGCTGTTTCAACGACAACTA
TCTCACCTGATATGTCAGATCTGGTGACCTCACTGGTCCCTAGTTCTGGG
ACAGACACCAGTACAACCTTCCCAACATTGAGTGAGACCCCATATGAACC
AGAGACTACAGCCACGTGGCTCACTCATCCTGCAGAAACCAGCACAACGG
TTTCTGGGACAATTCCCAACTTTTCCCATAGGGGATCAGACACTGCACCC
TCAATGGTCACCAGTCCTGGAGTAGACACGAGGTCAGGTGTTCCAACTAC
AACCATCCCACCCAGTATACCAGGGGTAGTGACCTCACAGGTCACTAGTT
CTGCAACAGACACTAGTACAGCTATTCCAACTTTGACTCCTTCTCCTGGT
GAACCAGAGACCACAGCCTCATCAGCTACCCATCCTGGGACACAGACTGG
CTTCACTGTTCCAATTCGGACTGTTCCCTCTAGTGAGCCAGATACAATGG
CTTCCTGGGTCACTCATCCTCCACAGACCAGCACACCTGTTTCCAGAACA
ACCTCCAGTTTTTCCCATAGTAGTCCAGATGCCACACCTGTAATGGCCAC
CAGTCCTAGGACAGAAGCCAGTTCAGCTGTACTGACAACAATCTCACCTG
GTGCACCAGAGATGGTGACTTCACAGATCACTAGTTCTGGGGCAGCAACC
AGTACAACTGTTCCAACTTTGACTCATTCTCCTGGTATGCCAGAGACCAC
AGCCTTATTGAGCACCCATCCCAGAACAGAGACAAGTAAAACATTTCCTG
CTTCAACTGTGTTTCCTCAAGTATCAGAGACCACAGCCTCACTCACCATT
AGACCTGGTGCAGAGACTAGCACAGCTCTCCCAACTCAGACAACATCCTC
TCTCTTCACCCTACTTGTAACTGGAACCAGCAGAGTTGATCTAAGTCCAA
CTGCTTCACCTGGTGTTTCTGCAAAAACAGCCCCACTTTCCACCCATCCA
GGGACAGAAACCAGCACAATGATTCCAACTTCAACTCTTTCCCTTGGTTT
ACTAGAGACTACAGGCTTACTGGCCACCAGCTCTTCAGCAGAGACCAGCA
CGAGTACTCTAACTCTGACTGTTTCCCCTGCTGTCTCTGGGCTTTCCAGT
GCCTCTATAACAACTGATAAGCCCCAAACTGTGACCTCCTGGAACACAGA
AACCTCACCATCTGTAACTTCAGTTGGACCCCCAGAATTTTCCAGGACTG

TCACAGGCACCACTATGACCTTGATACCATCAGAGATGCCAACACCACCT
AAAACCAGTCATGGAGAAGGAGTGAGTCCAACCACTATCTTGAGAACTAC
AATGGTTGAAGCCACTAATTTAGCTACCACAGGTTCCAGTCCCACTGTGG
CCAAGACAACAACCACCTTCAATACACTGGCTGGAAGCCTCTTTACTCCT
CTGACCACACCTGGGATGTCCACCTTGGCCTCTGAGAGTGTGACCTCAAG
AACAAgtaagaataacttttttattgtggtaaaatataaatactataaaa
attgccattctaaacattttaattgtacaactcagcagtactaatacatt
cacattgttgtgcaaccctcaccactatctgttttcaaaactttttttat
caccccaaacaggactgaaggaataatttcccattccccattctccctag
tgcagtggtgcaatctcggctcaccacaacctctgaacctctgtctcctg
ggttcaagcaattctcctgcatcagcctcctgagtagttgggactacagg
tgcacgccaccgtgcctggctaatttttgtatttttagtacagacagggt
tttaccatgttggtcaggctggtctcaaactcctgacctcaggtggtcca
cacgccttggcctcccaaagtgctgggattacaagtgtgagacactgtgc
ccggccatatctgttagatcttactaatcctgtcaagaggattcagtgtc
cttttttttttttctttcttttttttgatagagtctccctctggcaccca
ggctggagtgcagtggtacggtcttggctcactgcagcctccacctccca
gactgaagcgattctcctgcctcagcctcccgaatagctgggactacagg
cgcgtgccaccacgcccagctaatttttgcatttttagtagagatgggat
ttcactatgttggccaggctggtctcaaactcctgatctcaagtgatccg
cccaagggcctcccaaagtactgggattacaggtaggagccacctcacct
ggccctattttcggaatggatttttttttaatgtttaaaatgtcacctaa
gattattgtgaagatcaaataagataaaatcctaataacccaagtaaacc
acagggctccacttggaccagtctcagaagtttcaagaaaatcagtcaga
ccatcaaatgtaaaataagtctaaattttctttgcactattcacagagtg
ccaaagaggatctaattcatgtttcagaacataccctacttactaaaatc
ccctttcctcatttcttctcattctgcaactttatcatctcctgcggac
cccctagcctctcccctccccatagtcagtctctctctctctctttccct
cccctcttattatctcaatttcacacgaaagaattccagaaactatactg
ccaaaagtctttcctgtctttgaaaagttgggaaagaggagaaactcaga
cagcaatgacaaaattatacgtaatggatgaaggaaacacaaataaggct
ggaaacagaaaattttgtccccatcatttatttaatgaaggtggcagtat
tccagccacatagtgaacccccacaataagaagggcctctggcgattga
ttattgtcattgttgttaatgataatgagggtgaggatatcatgagcatc
agtgtaggaggcagttaactaataagaccaagctgttggctgggcgtgct
ggttcacacctgcagtcccagcactttgggaggccaaagtgggtggatca
cttgaggtcaggagttcaagactagcctggccaacatggtgaaacctggt
ctctaccaaaaatacaaaaattagtcaggtgtggtggcgtgtgcctgtaa
tgacaactacttgggaggctgaggcaggagaatcacttgaacctgggagg
cggaggctgcagtgagatgagcttgaaccactgcactccagcccgggcaa
cagagagagactcttgtctcaaaaaacaaaacaaacaaacaaaaactaaa
ccaaacaaaaaaagactagctgttattcatttatttatttatttatttag
agacggagtctcgctctgtcacccaggctggagtgcagcggcacaatctt
ggctcactgcaacctctgcctcccaggttcatgtgattctcccgcctcag
cctccccagctgttgttattcatgaatgaacctcagagaaagcacacagg
agggttggtgcacctgtgttttgagttctacccctccttcctctcttaac
ttcctcctgtcttctcactctgattcgttcttccttcctctccctctctc

tctgcagGTTATAACCATCGGTCCTGGATCTCCACCACCAGCAgtgagta
aacatggccctgaagtccctatgccctgggaattcttcctccctaagcct
gccttccaggaggaaagtatcccccattccctaggttctcatccccacag
aaactccagaatagcaaaagtctcaggctgagccaaggcacagatgccag
tgctcaccaagagtcctattctcccctcgctaaatgataggacccaacaa
acccgattcacgctgcgttttctttcagctccgatgacctccatgttctc
tccaaggcctctcgtatctgtgagccccacccccagcgctacaggtagga
atctggcttccagctcccatgaaacgtcggctgccattcagtggctgatt
aattgctgtgtggtctgagtcctgatgcccaccaagtctcagcgtgttcc
cctctgtccaatctcatccaacaatttaagctaatgcttgtttaatgatg
tcctcactataccaccttggacactttctttttgcctggatttaaagctt
ccatttctttccttccttccttcttttcttccttccttccttccttcctt
ccttccttccttccttccttccttccttccttccttcctccttccttcct
tcctttcttcctttcttcctgtctttttctttctttccttcttttggcag
agtctcactctgtcgcccaggctggagtgcaatggtgcaatctcggttca
ctgcaacctctgcctcccaggttcaagcgattctcatgccacatgccact
atgcctggctaatttttgtttttttgttttttgggggtttttttgagaca
gagtctcagtctgttgcccaagctggagtgcagtggcatgatctcgggtc
actgcaacctccttctcccaggttcaagcgatttcctgcctcagcctcc
tgagtagctggaactacaggcacgcaccatcacaccggctaattttttgt
gtttttagtagagacgacggttttgcaatgtgggccaggcttgtctcgaa
ctcctgacctcaagtgatcctccagcctcggcctctcaaagtgctgggat
tacaagtgtgagccactgcaccaggccaaaaacttgtatttcaatagtca
ttgaggctgggtgcagtggctcacgcctgtaatcccagcactttgggagg
ctgaggccagtggatcatgaggtcaggagatcaagaccaccctggctaac
acagtgaaaccccatctctactaaaaatacacaaaaaaattagccgggca
tggtggcagatgcctgtagtcccagctactcaggaggctgaggcaggaga
atggcgtgaacctgggaggcagagcttgcagtgagcggagatcgcaccgc
tgcactccagcctgggcaacagagagcgactctgtctcaaaaaaaaaaat
atatatatatatatatatatattcattgagaccgactctgacttaaaagc
agtaatgaatggtgtaggttttggtaaattacaggtcttgctttaagtcc
tggtcctctcttttgctcactgtgtggccccggaagagccatgtaacctc
tccaggcttcagtgtccattttagaacggagtaagtgaataagctgtgt
ccaatcatctctggccatatcagcttcatttttttttttcctccaggtcc
aaacatccctccaccctcagagtctttgcacctggtgttcttgtccttca
aatctcagcttggatcaccctttataaagtagcatttcccccgtatacgc
atcttgcacacagccaatctctattctacctctatgctcacttccttcct
ggcaattattactacagctgggcccttgaacagcatgagggttcagggtg
ctgacccctatgcattcaaaatccacatataacttttttttttttttgaga
tggagtttcacacttgttgcccaggctggagtgcagtggcgccatcttgg
ctcactgcaaactctgcctcctgggttcaagtgattctcctgcctcagcc
tcctgagtagctgggattacaggcatgtgccaccatgcccagctaatttt
gtatttttagtagagatgaggtttctccatgttcgccaggctgctcttga
actcctgacttcaggtgatccgcctgccttggcctcccaaagtgctggga
ttacaggcatgagccatgatgcccggccatttgctaatggcatctagtaa
gtagaggccagagatgttgcaaaacatccaacaatgcacaaagcagcctc
ctatcaaaacacattatccagaccaaaatgtcaatagggctgaggttgag

catctgctgtacacagattccaagttctggtacaaatctcgtagttctct
gagggctcatctttcaatgcctagcacatcaaaggaggccaatttcctct
tccctttcacctcctggtatgaaatgtttcctcctccaccttgatcctgt
aagagcccagctggagtttgcagacgacggggaaagaaatgggtgaggga
gggtcctatggttgagtctccgcagtgggccctgggtgcccagttcaccc
tcctccccttcatttctccatcatgacaactcaaggcaaattctcagtt
tccatgggccagtggaatccactgacttcatgaaataaccccaccctgag
caaatacccctcaaataataactgtttacacaacatcagtggcaacaatg
acccaagcagcaatgccaccaccagaatagcaaccataacagcagctcat
tttcatcaaaaggaaactgtagggccaggcacagtggctcacacctatat
tcccagcattttgggaggctgaggcaggcagatcacctgaggtcaggagt
tcaagaccagcccagccaacatggtgaaaccccatctctactaaaaatac
aaaaactagccaggcttggtggcatgtgcctgtaatcctagctactcggg
aggctgaggcaggagaattgcttgaacctgggaggcagaggttgcagtga
gctgagattgtgccactgcactccagcctgggcgacagagcaagactccg
tctgaaaaaaaaaaaaaaaggaattgtgccaggaattgtgatgagaactt
tatatgcattatctcctattaatattacccaaacctccgtgagttactat
actcatttctacagagagcatttatgcatccagggaggaagtaattagcc
cagaattactcagttatgacacaggacagtatgaaaactccaaccgaaga
ttggagactcatgaaaactccaggctcctaactacaagacatcactgtgg
atcgtccaaatagagcaagccccaatctcaggacaggaatgaggcatgaa
tggcctctatgctaatgatctaacctaatgctgaatttgttacttccctt
ctgaatccacttggagatttcctttatatctgacttgaaatagaggatat
atactcctctatccttgacataggagataatacacagaaagtatttcatt
gtagtatcaagtacacatcctgttctgtgtccataggattatgactaatt
tagggcatggcttaacagtgtggtactattgaatgacagacagatgtctg
ttttgttggatgcaggacaagccatgtaacctccccagactttagtgtcc
cctctgtggaatggaataaaaatactacgtgggattgttctgataatcaa
atgagataattcaggaacaacccagataaataacagggctgccctgggtt
ctgtctttccttgtatctctcacagagcctcaaaggagatgcaatccatg
acctagagaaacactcaggacaaattctcttttccccagttcctttcttg
ctccaatggcaacaccacccctctcatcctgaagtctcttgtttttacca
ccacacctattttgccaaatttttctccaatattccaaaccatatgaaacc
tttctttctttcttttctttccttcctttccttctttctttcttttttct
cttcttttcttttctttttgagacatggtctcactctgttgcacaggctg
gagtgcaatggcacgatctttgctcactgcaacctccgcctcccaggttc
aagagattctcttgcctcagcctcctgagtagctgggattacaggcgccc
accgccacgccacgctaatttttgtgttcttagtggagacggggtttcgc
catgttggccaggctggtcttgaactcctgacctcaagtgatttgcccat
ctcggtctcccaaagtgctaggattacaggcgtgagccaccaagcccggc
cccatatgaaccgtttctatccctcatttctctgtacttttacctaaaaa
caccactcccttcacccatcacatttttgtcaattctacatcacacacac
acacacacacacacacacacacacagagaaagtaagttggaaaaaaatta
tactatcatgaaattttgtgaaaggaggtaagctgagagagtaagaatca
aactaaattatctttatgggtagaaagcacactcatccatacatgtgtct
ttccacccttgtaatgtatttattattattgtttgtatatactagattcc
caataaatagggacagctattatggtatttttatttcaggaataataata

gtgatgatttccaccattattgtcaaaggacaaagcacaaaatatgtacc
aaataaaatatagccattatcctttattcacaaaagatcttggccccacc
tcttctcaatgaaatgtccatgacttgttcaactttggccactctgggct
gagagatggaggttcccttgcgagctgaagtcacacatcgaaggtggaag
cccctcccctccctctggctggctgagggatagcccagatgggctcatca
tgaaagtttcccattatttccatttctggatctaccatcttcccctcccc
tacctctcacccatcataattgtccttctttactctttcctccctatctg
cagGTTATAACCGTCGGTACTGGACCCCTGCCACCAGCAgtgagtattca
aacctgtgatattccaatgcccttgggacccttcctccccaaggtgcatt
cctcagaagagaaactgatcattctccctccctacgtgcccagccacagc
ctcagagcagcccctaacccgtcaaggtcttggtgtgagtcaagatagaa
gtccaaattccaatgagcagttcctgtcccatattcctttaggaagacac
ccaatcatttctccatgttctttttttctcagCTCCAGTGACTTCTACAT
TCTCCCCAGGGATTTCCACATCCTCCATCCCCAGCTCCACAGgtaggaag
ctcctctctggcatctatgaaatttaacactgcatggtctgttccctgct
gaccacccagactcagcctgttccactcgccctctcactctctctctctc
tctttttttttttttttttttttttttttacggagtcttgctctgtcac
ccaggctggagtggaatggtgtgatctcggctcactgcaaccttcgcctc
ccaggttcacgtgattctcctgcctcagcctccggagtagctgggattac
aggtgcacaccaccatgcctggctaatttttgtatttttagtagagacg
gggtttcaccatgttggccaggctggtcttgaactcctgacctcaagtga
tctacccaccttggcctcccaaagtgctgggattataggcatgagccacc
acgccaggcccactctctaaattttgaccaccctgccttgagtggtcttc
tagcaccctaacctctgtctaacctcgagagctttgcactagcgattcct
ggggaccagctatggttggtatcttctcaactttctaattttttttaaaat
tattattattattattattatttaaatggagtctcgctctgtcacccag
gctggagtgcagtggcaccatctcggctcattgcaacctctacctcccgg
gttcatgcaattttcctgcctcagccagaaattttctcagtggtcgagat
tgtgccactgcactccagcctgggcaatggagctaggctccatctcaaaa
aaaaaaaaaaaaagacggaggtcgggcattcctaacccttaaccctgcct
tgtgattctggagttatgagatagaacctggtgtcccgtaattaaaattc
cgccttcaggccttatgttttgtgagtcacaacactgcaaactttttaca
tgctgtagacaggatgttcactctccacttcctcactgctctgctctaat
caattcaaccatttatgtgacatgcctaacccctctgggcttgtacgtat
gtaacatgtattacaaagcaagtcattccatgatcaatgctgtcactttt
tctaggtgctttcaaaatttgttcttcatcattgattttcagtagtttga
ttacgatgtgtctgggcatggttttctttgagtttatcctgcttaaagtg
ttctcagcttcttgagtctcaaagtgtttattttctgctctgattctttc
tccccttcggacctccaatgaaatgatgttgcccgaagagaccctgaggt
tctgttcattttgttatttatcaatctttttttcctctccgaatttcaggt
ttaataattttttttttttttttgagacggagtctcgctctgtcgcccag
gctggagtgcagtggcgcgatctcggctcaccgcaagctccgcccccctgg
gttcacgccattctcctgcctcagcctccggagtagctgggattacaggc
acccgccaccatgcccggctaatttttgtatttttagtagagacgggg
tttcaccgtattagccaggatggtctcaatctcctgacctcgtgatccgc
ccgcctcagcctcctaaagagctgggattacaggcgtgagccactgcgcc
cggcccaggtttaataattttttatagaatattttcacaatcaccaagcct

tttctctaccagctccattctgcccatccattgaattctttttatctcag
ttactttatgtttcagttcgaaagtttctacttggttagatagatagatg
ttatatcatatattatatgttatataaaaatatatttatggttatacata
taacatatatgttatatatagttatttatatagccataactatatatagc
catatatatagttatatataaccatatatatagttaccatatagtaacca
catatataaaacatatatatatagtgtctctctatatatagttatatata
tagtttctatatctgtaactatatatagttatatatgtatgtttctctgt
atataaatatatatatttctatatatatagttatacacattatatatata
actgggagatgttggtaaaggatggcgtgaggaaacctggagcagtcatg
gtaatcctcgctctgctccgaactcctcaagagcaggagaagggtcctcc
tcattctccagccatgttgactttgagcaatttactcatcctctcagtac
ctcagtttcctcacctgccaattgaggataataatatttcataaattgtt
tgcaaatgttatatgcaactctacgtaagaacacctagcacaggggctac
cagggaatttggtttaacaaatatttatcaggcacctattctgggctggg
caggggggataagatgttgactaagtcaaatgcagtccctcccctcacca
agtttacagtgtattgggcaagactgaaatggaacaagcaattacaattg
acaataaaagacaaccaagttattgagcacttactatatggcatgccata
tgctatgtattttttttattttttaacttttcattttgaaataaataataa
atataaagtaaataataatataaataaataataaataacttttcattttg
aaataaataataaataaattcaggagatgttgcgaaaatagtgtagcatt
cccctgtatccttcacccagtttctccccaatggctacatcttacataac
tctaatacaatatcaaaagcaggaaactgacattgttaaaatccatttta
ctggttttacacgcgtgtgtgcatatgtgagcttgtgtatgtgcgtgtgt
gtgcaggcatgtgtgtgcatgcacgcctgtgtgtgcatatgtgcatgtgt
gcatgcgtgtgtgcatgtgtgcatgtgtgtgtgcatgcgtgcgtgcgtgc
gtgcatctgtgtgcatgtatgcacatgtgtgtgtgtctgtgcacgtgtgt
gcatgcatgtgtgtgtgcgtgtgtgttggtagccctatgcaatttttatc
acatgggcatagccctataatcaccaccaccatcaagattcagaactgtt
ccattcccccaaagattcccctcatgctagccttcgtaatcatgcccact
gagcccaacactattgcatagaatagctattctactctccatctccatct
ctgtctctacaattttcttttgaagatgttatataaatggaaatgtacaa
catgtcacctttgaaattggcttcttttccactcagtgtaatgccctgga
gatgtgctctttttaacagtcatgtaaccttcctaatttccctccaaaat
atcattatgcccctcgccgccttttttttttttttttttttttgagacag
agtctcgctctgttgcccaggctggagtgcagtggtataatctcagctca
ctgcagcctccgtctcccgggttcaagggattcccctgcctcagcctccc
aagtagccaggattacaagtgcatgccaccacgcctggctaatttttgta
tttttagtcgagacggggtttcattgtgttggccaggctggtctcgaatt
cctgacctcaagtgatctgcccgccttggcctcccaaagtgctgggatta
caggtgtgagccaccgcgcccgacccatattgcccattgtattacagcgg
aagaaactgaggtatggacaggtaacatgtccatggtcacttggctggtg
aggggcagagaggagatttgaaaccaaatctgactcactagtgtggccgt
aaccatggtaactatgtctctctaccatgtggtctcctctttattaaagg
aagggcaagttctgggagttttgggagttttgggcttgagtggggaaggg
tagccaagtaaagcaggtgagagaaggtctgctttaaggactgctgtttg
atttttattgttgttgttcagtgttcaatgggattgagttgactcttttt
tcccttcttgttccccaaagcatgagactgttccggtccttttcccttt

aacttctcagctagagtttgttagggcgggtatgggcacctggcagagtc
tgagacctcagcttccagtaggcacacgttctgacccaatacacctaccc
tggtcccctaacctgcttctggtcccctaacctgcttctgggcccaggta
atgcattttaggaacatcccacttttctccttacctggctttccattatc
cgtccaaactaaagcacccacctgtctgcttcagactcttgcttcaagca
ctccgtctgggtcctcagaaattgacttacagtcagttcagatctgactc
aggcgtggccttcttttctccttccttgcagCAGCCACAGTCCCATTCAT
GGTGCCATTCACCCTCAACTTCACCATCACCAACCTGCAGTACGAGGAGG
ACATGCGGCACCCTGGTTCCAGGAAGTTCAACGCCACAGAGAGAGAACTG
CAGGGTCTGgtgagagccccgcccaccgtactcctccctcgcccacttag
acaaaccagcccacctcacactgcctcgcccactgatgccagccacgccc
acctcatccaaccccagacacctttccctgccccacccactgattttagc
caagcccacctcaccccacccagcctactgatgccagccacgcccacctt
tccctgccccgcccactgatttcagccacgcccacctcaccctggtccac
ccctccaatgccccactcttcctggcttcccgcagctgttgtttctcacc
tcccctctccttccttgcagCTCAAACCCTTGTTCAGGAATAGCAGTCTG
GAATACCTCTATTCAGGCTGCAGACTAGCCTCACTCAGgtgagacgctcc
ttaagaaaaacacagcccaacaggtgaatatgaccctagtctctgggctc
cctgactctgttcatacttggaacaactattgcccatggatactaagcat
caccaccagcagcagcagataactattcctaagacccaaggcactgcatt
atgtactttatatttaatgcctcatcagtgcttgcaacagcctcatgaag
caggagcagaaggggaaactgaggcccagattaagtggcttgtgccagga
cacacaaagcaactgcagcacttcaggttctatatccaaactcctatccc
ttaggtggcacttcctcctctgcccccattatgaacttgcagcatgtgga
aaaccccaatctgacttccctctaagggaacttgcccagagaatctaaga
ggggaggaaaggaaggcgttcagcccttacaggcaggaggtcagctcctg
agtggctcagatgcagccacagagggcctggccggtctgagggtgactga
gaggcaccgagggcactgtccctgagtgctggaaagggcaggtcttttag
ggtagacagcggttgatatcatttcctgcctggcattctcaccttccaca
cctctctcacagaatctccaagtgtggctctcccaagagagagtgtcagt
catctacctccagcttcctttccttcccaggggggaagaggggacaggggg
gccctagtggctaagagcattggtgaactcaggcagacctcagttctgaa
ccaacccagctctgccatttactatctgtgactctgagcaagtgcctgaa
gccttctgtgccctatttcctgacatattatatatataaaatacatatat
tatatatagacatattttatatacatattgaggcatattttataaacatg
tttatagacacatttttatatgcatatgttatatacgtatataacatatg
ttatatataatgtatatattatacatattgttatattgtatacatgttat
atatgttatagcatatatagtacaagttatatataacacatacattatgt
tacatataatgtatatgttatatatgatatattatatataattatatatt
atataaaactgttatatataattatatataatatatagttgttatatata
attatataattgttatatattatatacaacatataacatacattatatat
tgttatatataatataatatatacatatataacatatgtataacttttat
gttatacataatgtatataacatatatgtgtatgtgtgatgtacataaca
tatctgacattaacatataacatatgatataacaatattatatgttataa
cataatatatgttataatataacaatattatatgttataacttatactgt
catatgtaacatatacataatattttataaatcagtttaatatacattat
gttacatataatgtatgttatatatgatatattatatataattatattat

acataattgttatatataatgcatacattgtatttgttacgtattatatg
caacatataacatatacattatgtattgttatatataatgtaatatatac
atacataacatatgtataacttatatgttatatataatgtatataacata
tatgtgtatgtgatgtatataacatatctgacattaacatataacatatg
ttataatatgacatattatatatattacatataacgtatatcatgtataa
tataatgtgtatatataatatattaaagtatataagtataaatacatgta
atatttaaatatatattatatatagtatacatgtggatacatacaacttc
tacatatacctagtatatattctatatataaacagtccatgaattacaat
gattcaacttatgattttcaaactttgtgataatgccatagcaatatgc
attcagtagaaagcataccttcaacacccatgcaaccattctgtcattca
ctttcagtacaatattcaataaattatatgagatattcaacagtttatta
taaaataggctttgtgttaggtgattttgcccacatgtaggctaatgtaa
gggttcagagcatgtttaaggtaggataggctaacctatcatgttctgta
ggttaggtatagtcgatttttatttttatttttatttttgagacagagtc
ttgctctgtcacccagactggaatgcactggtgcgatcatagctcactgc
agccttgaactcctgggctcaagtgatcctcctacctcagcctcctgagt
agctgggactacaggtgtgtgccaccacacctggctatttttttttttaat
tttttttttttttgtggagaggagggtcttgccatgttgcccaggtggcct
tgaactcctgggctcaaggaatcctcccaccttggcctcccaaaatcctg
ggattacaggtgtgagccatcacgcccggctacagggcatttttgactta
tgacattttcagttcacaatggatttgtcagggctgggcatgatggctca
cacctgtcatcccagcactttgggaggctgaggcaggtggatcacttgag
gccaggagtttgagaccaggctgtccaaatggcaaaatcttgtctctact
aaaaatacaaaaattagccaggcgtggtgtgacaactgtagttccagcta
ctcgggagactgaagcgtgagaatcacttgaacttaggagatggaagtta
cagtgagtcaagatcacaccaccgcactccagcctggatgacagagcaag
actcttgtctccaaaaaacaaaaaacaggctgggtgcatggctcatgcct
gtaatcccagcagtttgggaagctgaggcaggtttatcacctgaggtcag
tagttcacgatcagcttggcaaacatggagaaaacccatctctactaaaa
atacaaaaattagctggatgtggtggtgggtacctgtagtcccagctact
cgggaggctgaggcaggagaatggattgaacctgggaggcagaggttgca
gtgagccaagatcacaccattgaactccagcctgggcaacagagtgagac
tccatctccaaaaacaaaagaaagcaaaaacaaaaaaataaaataaaaaa
cctgtgtttatcaggacataataccatcatgagtcaagaagcatctaaat
gtacatggtagttatataaaaatagttatatagttatatacaatagttat
atataaaccagtttaatatatgttaagtagaggtatatggtagttatata
aaaaatagttatataatagttatagagttatataattatataaaatagtt
atatataaaccagtttaatatatgttaggtagaggtataataatatatat
tgtatatactatataatatagtaatgtataaaatgcaaaacgatatcata
tatttctatattaagtttatatttacagatctacattttatatattttat
gttatatacaattgtgttatacataatataattagtatagtactgacttg
gggaattgagcagtaccaacccatagggatgtttgaggatgaaaatatgt
gattatgaatacaaaatgctgggcctgctgcataggaagtatttaataaa
tggtagttgttactataaagtcgttcctactatagagctactcacaaccc
tgggacatagggaaagagcccgtttccctctaatcactcaatagtgggtg
gctaggtaggtgagtccacatcctgtggccgggaacaggtgctgagacat
gaagaccttctgactgcatgttggaccagccacagtttcagacggaccag

ccaaaaagggcattttccccaagccatttagctcccttgagtctcataac
aaatctcctagccctgctggtccataggatctagagaggatgacttgaac
cttctgatcccaccatttgaaaacgccatgccatgggcaccagtaggagg
gccactgctacgtgcaccagtacaagggccactgccatggattacagatt
aaccctaagtatagctgtcgcacacctagtacttcaggaggcttattcgg
ggccatgcagatccctggcattattatcctaggatcctacaccaagcaaa
gcaggagctgcccctcctcataaacccataagccctcctcttgagcaaag
cagctgggaaggccagaagttattcaagctcccctctgccccggttccaa
agacagacagctcaagcctacatgcagcaaaccctataaaagtgtcacct
cttggcatttctgccatggtaatgctttctgcttccactaataatcctag
taatttgtttatggtgggcatctctctgatgagaaccacattcttttttt
ttttttttttttttttgagatagagtctcactctgttgcccagactgga
gtgcagtggcgcgatctcggctcactgtaacctttggctcctaggttcaa
gcaattctcctgcctcagcctcccaagtagctgggactgcaggcacgtac
caccatgcccagctaatttttgtatttttagttgagacggggtttcacca
tgttagccaggatggtctcaatctcttgacctcatgatccacctgccttg
gcctcccaaagtgttgggattacaggcatgagccaccatgcctagcctga
gagccacattcttgttaaccacaattttctcagagtctgcattaggggtt
gacaaagagtggaaaggaaggacaaaaggatggagaggtggatggactaa
gcatatgtaggttcttacccagGCCAGAGAAGGATAGCTCAGCCACGGCA
GTGGATGCCATCTGCACACATCGCCCTGACCCTGAAGACCTCGGACTGGA
CAGAGAGCGACTGTACTGGGAGCTGAGCAATCTGACAAATGGCATCCAGG
AGCTGGGCCCCTACACCCTGGACCGGAACAGTCTCTATGTCAATGgtgag
cagctgtgatgtggttggaggctcttcctccttgctgagcagcctgtaat
cactggcttgaggtcacactcactgtcaggcaattgaaaatttggtcctg
tgctctacatgggatgactaatttccggacttcatggtatcttttttttt
ttttttttttttgagatggagtctcgctctgtcaccaggctgaggtgc
agtggcatgatctcagctcactgcaacctccgcctcccggattcaagcaa
ttctcctgcctcagcctcctgagtagctgggactacaggtgcatgccacc
acacccagctaatttttgtatttttagtagagacagggtttcaccatgtt
ggtcaggatggtctcaatctcttgaccttctactccaccttgccttggcc
tcccaaagtactgggattacaggcttgagccaccacacctggccaggact
tcatggtttcttcatcatcatggaatgaattccatcagggcattcttccc
tgatgtgagggcactgataggaaatctttaatggtccctgctgcatgaaa
ctgcttccattgcaccagggtagccctgacccctatttggtcccccacat
ctccttgtaacttacccacactcctccctccttctctgtgcagGTTTCAC
CCATCGAAGCTCTATGCCCACCACCAGCAgtgagtattcaactcatgtcc
acatgcccatgatcctacaccaagcaaagcaggagctgcccctcctcata
aacccataagtcctcctcttgagcaaagtagctgggaaggcagaagttat
tcaagctcccctctgccccagtttcaaagacagactcagctcaagcccac
atgcagcaaaccctataaaagtctcacctcttggcatttctgccatggta
atgctttctgctctcactaatgaggacttctcctcagCTCCTGGGACCTC
CACAGTGGATGTGGGAACCTCAGGGACTCCATCCTCCAGCCCCAGCCCCA
CGAgtaagtaccagtcaatggcatctctattagagcatgctatctctgtc
atttttactcagatgaagatggaaaatcatagcaaatctactgatagtga
gtggaccaacgaaatttgttggccacctagtgtgtaccagatcctagaga
tacaggagggaaaacaaaaccaatacaaaatttctgctctcagtgagctt

gtattcttgtcatgatgatgatgttggtggtggtgctgttgatgacgatg
atgatgatgatgatgatgatgatgctggtgatactgttgatggtgatagt
gatgttgatgacaatgatgatgatgatgatgttgaagaaaatgatgctgg
tgatggtggtgggggttattatggtaataatgatatgttgagtgtgacga
tgatggtggtggtgttgatgatgatgatgattattatgctagtgacattg
atgatggtaatggtgatatcaacgacagtgacaatgatggtgatgaggat
gatgtcggtgatggtggtggggttatgatggtaatgatatgttgaatgtg
atgatggtgatgatgatatttgtggttcatgatggggattgtcatggtgg
tgctggtggtacttgtgatgacaataatgataataatgatgacaatgata
gtgatgatggtgatggtgataataaagataacagatatcaccttacaata
ttgagcactaaatatgtaccaagagctatgctcagtatctaactactatt
atataatctactttagaaaatgaattgtatcatagataagaaaggcgtgg
aaaatatttattatgtcactcaatttaattgctgcatatggttattacaa
agtgctattctctctactttgaacataatgtttatttcacactcccacta
tagCTGCTGGCCCTCTCCTGATGCCGTTCACCCTCAACTTCACCATCACC
AACCTGCAGTACGAGGAGGACATGCGTCGCACTGGCTCCAGGAAGTTCAA
CACCATGGAGAGTGTCCTGCAGGGTCTGgttagtgtcctgccctccacac
tctgccctgctcatgatacccagtccctcttacatcatccatgccagggc
aatggaagaatatcaaacccaactcacttttgccccaagagatgcaagcc
tcagccaggagcggtggctcacgcctgtaataccagcatttgggaggcca
aggcgggtggatcacctgaggtcaggagtttgtgaccagcctggccaaca
tagtgaaacctcatccctactaaaatacaaaaattagccaagcatggtgg
tgcatgcctgtaatcccagctacttgggagggtgaggcaagagaatcact
tgaatcaaggaggcagaggttgcagtgagtcaagatcatgccactttact
ccagcctaggcaaaaaagcgaaactccatctcacaaaaaaaagaaaaaaa
gagagagatgcaagcctcccccaccaaggccagccctgcccacctcactt
ctgcctggctcttacataaaacttagccctcctactcactgccctctccc
tcctccacagCTCAAGCCCTTGTTCAAGAACACCAGTGTTGGCCCTCTGT
ACTCTGGCTGCAGATTGACCTTGCTCAGgtgagaacttagaatttccagc
ctggctgccccacttgtactcactccaaaagactttgcactgcttccttg
ctgcacttcctagggatatcctcaccaaaggtggaattcaggagtcacag
gcttcaggatcagtgtgtttcctgacagtaacacccctacactccacctc
aacagagagaatctgcatggcccatcatcaggattgagcctctcccttta
tcatccctctgaattccctccattccctgtgcctccctttcctttacatg
ttaaattctgtccccaggatttctttcaggacaatcatgccttatccacg
tgatttcatcctcatttcgagctcttcactgggctcaagtccggctcccc
gtcccgtccatgaaagtgtcagtttcatcttgtcactgtatccgtgactc
cactcacagtcctcagcaagccaatagtccatgcactaagagtcgatgtg
gcttctcacctctttcccaggtttctcatttctctggtccttgctgtcct
tccctcagcaatcgcaagacccttcctagataaacttttcattgtgattt
ttcccactgaccctccccagGCCCGAGAAAGATGGGGCAGCCACTGGAGT
GGATGCCATCTGCACCCACCGCCTTGACCCCAAAAGCCCTGGACTCAACA
GGGAGCAGCTGTACTGGGAGCTAAGCAAACTGACCAATGACATTGAAGAG
CTGGGCCCCTACACCCTGGACAGGAACAGTCTCTATGTCAATGgtgagtg
gctgtgatgtggttgaaatctcttccccttgctgggcagcctctaatct
ctaactagagatcacactccctgcctggcctttgaaaattctgtcatgtg
ctctacatgggatgactaaggtctggacttcatggtttccttaccatcat

ggactgtgttccctcagggcattctttcctgatgtgaggatgctgataga
aaatcttcaattgtccctgtaccatgaaactcggttcattgcaccagggt
agcattgacctccatttggtcccccacctctccttgtctcttacccactc
tcctccctccttctctatgcagGTTTCACCCATCAGAGCTCTGTGTCCAC
CACCAGCAgtgagtattcaactcatatccacatgcctcggttcctacacc
aagaggagcaggagctggcccctcctcataaacccattaagtcctcttca
taagcaaaggatttaggagggcagaagttatttaagtgtccctctgccca
gctcaagagaccgacccagctcaagctacacatgcaacaaaccccataaa
tagtctcccctcttgccatttctgccaagagagtgctttatgctttcact
gatgagaacttttcctcagCTCCTGGGACCTCCACAGTGGATCTCAGAAC
CTCAGGGACTCCATCCTCCCTCTCCAGCCCCACAAgtaagtatcagtcaa
tgacatctctatgagagcatacctgattagtgtaaacatctctgtcattt
tcactcaaataaagatggaaaatcatagtaaatctagtgatactgagtgg
acaaatttgtttgtttgtttttctcatccttttcactttttttattata
ctttaagttttagggtacatgtgcacaatgtgcagtttagttacacatgt
atacatgtgccatgctggtgtgctgcacccatttgctcgtcatttagcat
taagtatatgtcctaatgctatccctccccctcccccaccctgcaaca
gtccccagagtgtgatgttccccttcctgtgtccacatgttctcattgtt
caattcccacctatgagtgagaacatgcggtatttggtttttttgtccttg
cgatagtttactgagaatgatgatttccaatttcatccatgtccctacag
agacatgaactcatcatttttttatggctgcatagtattccatggtgtgta
tgtgccacatttcttaatccagtctatcattgttggacatttgggttgg
ttccaagtctttgctattgtgaatagtgccacaataaacatacgtgtgca
tgtgtctttatagcggcatgatttataatcctttgggtatatacccagta
atgggatggctgggtcaaatggtatttctagttctagatccctgaggaat
cgccacactgacttccacaatggttgaactagtttacagtcccaccaaca
gtgtaaaagtgttcctatttctccacatcctctccagcacctgttgtttc
ctgacttttaatgattgccattctaactggtgtgagatggtatctcatt
gtggttttgatttgcatttctctgatagccagtgatggtgagcattttt
catgtgtttttggctgcataaatgtcttcttttgagaagtgtctgttca
tgtccttctcccacttttgttggggttgtttgctttttccttgtaaatg
tgtttgagttcattgtagattctggatattagccctttgtcagatgagta
ggttgtgaaaatttctcccattttataggttgcctgttcactccaatgg
tagtttcttttgctgtgcagaagctctttagtttaattagatcccatttg
tcaattttggcttttgttgccattgtttttggtgttttagacatgaagtc
cttgctcatgcctatgtcctaaatggtaatgcctaggttttcttctaggg
tttttatggttttaggtctaacgtttaagtctttaatccatcttgaatta
atttttgtataaggtgtaaggaagggatccagtttcagctttctacatat
ggctagccagttttcccagcaccatttattaaatagggaatcctttcccc
attgctcagatttgtcaaagatcagatagttgtagatatgtggcgttatt
tctgagggctctgttctgttccattgatctatatctctgttttggtacca
gtaccatgctgttttggttgctgtagccttgtagtatagtttgaagtcag
gtagcatgatgcctccagctttgttcttttggcttaggattgacttggcg
atgcgggctctttttttggttccatgtgaactttaaagtagtttttccaa
ttttgtgaagaaagtcatcggtatcttgatggggatggcattgaatctat
aaattaccctgggcagtatggccattttcccgatattgattcttcctacc
catgagcatggaatgtttttccatttgtttgtatcctcttttatttcatt

gagcagtggtttgtagttctccttgaagaggtccttcacgtcccttgtaa
gttgcattcctaggtattttattctctttgaagcaattatgaatgggagt
tcacttatgatttggctctgtgtttgtctattattggtgtataagaatgc
ttgtgacaaatttgttttggccacctactgtgtaccagaccccaggaata
cagtaagaaaaagaaaatcaatttaaaaaaaatctgtgccctcagtgagc
ttgtattcttgtgatgatgatgatggtggtggtagttacaatggtaatga
tgatgtgttgagtgggatgatgatgatggtggtggtgacattgtttatga
tgatgatgatgatgatggtcatactgttgatgatggcagtagtgatgttg
atgatgatggtgatggtgatgaggatgatgctggtgatgttagaggaggt
tatgatggtaattatgatgtattgagtgtgatgataatagtgatagtgtt
gctgtttataatgatgatagtggtgatcttgatgatggtggtggtgatga
tgacaatgatgatggtgatgaggatgatgcaggtgatggtggtggaggtt
gtgatggtaatgatgtcgtattgagtgtgatggtgatgatgatggtggta
gtgttgctgtttataatgatcatgataatgatagtgttgatgttgatgtt
gatggtggtggtgatgtcgatgacaatgatgatgatggtgataaggatga
ttcaagtgatggtggtggggttatgatggtaatgatgatgtgttgagagt
gatgatgtttgtggtggtcatgatggggattatcatggtggtgatgatga
taataatgatggtgacgtgacaatgatggtgatggtgatgatgatgataa
taaagttaacagaaaatgtcagacagtattgagcaataaatattcaccaa
cagctatgctcagcatctactattatataatatacttttacaaaaataaa
ttatattattataggcaagggaggcatggaaaatattttgtcactcaatt
taaattctgcatatattgaaagataagtctattgcaaactcctattttct
ctgctttgaacatagtgtttatttcccattccagTTATGGCTGCTGGCCC
TCTCCTGGTACCATTCACCCTCAACTTCACCATCACCAACCTGCAGTATG
GGGAGGACATGGGTCACCCTGGCTCCAGGAAGTTCAACACCACAGAGAGG
GTCCTGCAGGGTCTGgtgagagccccacccactttactcctgccccatcc
cagatacatcatctatgccagggctatggaagaagattgtatccatctca
cccttgcccacaaaagatgcaagccctgctcactgaggccagccatgccc
actggtgcctgctccacccacctgacttctgccccacacacatgcacctt
agccctcctactcactttcctctcgctcctccacagCTTGGTCCCATATT
CAAGAACACCAGTGTTGGCCCTCTGTACTCTGGCTGCAGACTGACCTCTC
TCAGgtgagaccttagaagatccagcctggctgccccagttgttcccact
ccagtagattttgctctgcttccttgctgcacctcctagggatatcctca
cccaaaggggaattcaggagtcactggcttctggaccaatgtgtttcctg
atagtaacactcccacacctcacctcaacagggagaatctgcatggtcca
tcatcaggattgagcctctatcctgatcatccctcggaattccctgcccc
tccctttcatttaggtgttaaattctgtccccagaatttctctcaagaca
atcatgcctcatccaagtgctttcatccctgtttctagctcttcactggt
ctcaagtctgggctctcctgtccccatgctatgagaatgcaggtttcacc
ttgcacttttataagcatggttgtatctgtgactctgtgcacagtcccaa
gcaagccagtagtccatgcactcagagaatctaagtgtagcttctcacct
ctttcccaggtttctcatttcctctggttctttactgtctttccatcagc
agtctcaggacacaacctaagtaatcttttcatagtcattctccccacct
accttccccagGTCTGAGAAGGATGGAGCAGCCACTGGAGTGGATGCCAT
CTGCATCCATCATCTTGACCCCAAAAGCCCTGGACTCAACAGAGAGCGGC
TGTACTGGGAGCTGAGCCAACTGACCAATGGCATCAAAGAGCTGGGCCCC
TACACCCTGGACAGGAACAGTCTCTATGTCAATGgtgagcagctgtgatg

tggttggagtcttttccttctagagtctggaaagaatctaatctgtggct
tgaagtcacactccctgcctggccattgaatattctgtcatgtggtgtag
atgggatgacaaagttctggacttcacagtttcttcattgtcgtgaactg
tgttccctcagggcactcttccctgttgtgaggatactgataggaattct
ttaatggccccagtcccatgaaactcattgtcccatgaaactcatttaat
tgcattgggattgccatgacccttattgtgtccctcatatctccttaacg
cttaccaagtctcctccctccttctctatgcagGTTTCACCCATCGGACC
TCTGTGCCCACCAGCAGCAgtgagtattcaactcatgtccacatgcccct
gatcctacattaagtggagcaggagctggcccctcctcttaaacccataa
gtcctcctcttgagcaaaggagctgggaaggcagaagttattgaagctcc
cttccaccctagctccaaagacaggcccagctcatgcccatatgcagcag
acctcataatagtctaccttcttgccatttctgccatgagattattttct
gctttcactgatgagcacttttttctcagCTCCTGGGACCTCCACAGTGGA
CCTTGGAACCTCAGGGACTCCATTCTCCCTCCCAAGCCCCGCAAgtaagt
actagtcaatggcatctcccttagattatgcctgatgagtgtgaacatct
gtgccattttcactcaaatgaaaatagaaaatcatagtaaatctagtgat
actgagtgaaccaaaaaaatttattggccacttacagtgtaccagacctt
agagatacaaggaaaagaaaactaataaaagcacctctgccctcagtgag
cttgtgttcatgtgatgatggtggtggtggttatgatagtaataatgaca
tgttgagtgtgatgatgattgatgatgatggtgatgctgttgatgatgat
ggtggtgatgttactggcaatgatgatgatggacataaggatgttgtcag
tgatggctgtgaaggttatgatggtaatgatggtgtgtggagtgtgatga
tgatgatgatgttgctgtttatgatggtgaaagtgatagcaacaatcatg
atggccagtcatcataaatataacagataacatcagacaatattgagcac
tgaatatgcatgattagctatgctcagcatctaactactattatataaca
tactttcataaaaataaattgtattattataggcaagggagacatggtaa
atattttgtttctcaatttaaattttgcacatgtttaaagataagtctat
tctaaacccctatttcttgactttgaacatagtgtttattttcaattccc
actacagCTGCTGGCCCTCTCCTGGTGCTGTTCACCCTCAACTTCACCAT
CACCAACCTGAAGTATGAGGAGGACATGCATCGCCCTGGCTCCAGGAAGT
TCAACACCACTGAGAGGGTCCTGCAGACTCTGgttagtgcccttccctcc
tcactctgcccagccccagatatccagtcccttctacatcatccatgcca
gggtgatgaaagaagatagcaacaacttccccccttcccccccaagagatg
caagccccacccacagagaccagtcctgcttattggtgcctgctccaccc
acctcacatctgccccgacacacacacaccttagccccactactcacctc
cctctccctcctctacagCTTGGTCCTATGTTCAAGAACACCAGTGTTGG
CCTTCTGTACTCTGGCTGCAGACTGACCTTGCTCAGgtgagactttagaa
gagccagcctgggtgcccaacttgttcccactctaaaagactttgcactg
cttccttgctgcacttcctaggtatatcttcaccaaaaggggaattcagg
agtcattggcttgagaaccagttgtttcctgatagtaacacccccatgcc
ccaactcaacatgcaaaatcttcatggttcatcatcaggattgagacact
accctgattacccatctgaattccctcctttccctgcccctccctttcat
ttaggtgttaaattctgtccccaggatttctctcaagataaccatgcctc
atccacatacatgcatccgcctttcaagctcatcactagtctgaagtctg
ggttctcctgttcccatgccatgagaatgcaggtttcaccttgcactttt
ataaaaattattatatccatgactctgcttgcagtcccaaccaagatagt
ggtctatgtactcagataatctaagtgcagattctcacctctttcccaga

tttctcatttcctctggttccttgatatgtttccctcagcaatctcaaga
caagtcctaggcaatcttttcattgtcattccccctcctaccttcctcag
GTCCGAGAAGGATGGAGCAGCCACTGGAGTGGATGCCATCTGCACCCACC
GTCTTGACCCCAAAAGCCCTGGAGTGGACAGGGAGCAGCTATACTGGGAG
CTGAGCCAGCTGACCAATGGCATCAAAGAGCTGGGCCCCTACACCCTGGA
CAGGAACAGTCTCTATGTCAATGgtgagcagctgtgatatggtaggggtc
tcttcctcctggctgtgcaacctctaatctctggcttgggggcacactcc
ctgcctggccattgaaaattctgtcacgtgctctacatgggatgactaag
ttctggacttcatggtttctttgttatcatgagaggcattccctctgggc
actcttccctgttgtgaggatgctgataggaaatcttaatgacccctgt
cccatgaaactcatttaattgcaccagggtagtcctgaactctatcgcgt
cccccacatctccttaacccttacccagtctcctccctccttctctatgc
agGTTTCACCCATTGGATCCCTGTGCCCACCAGCAGCAgtgagtattcaa
ctcatgtccagatgcccctgatcctacatcaagtggagcaagagctggcc
cctcctctttaacccataagtcctcctcttgagcaaatgagctgggaagg
cagaagttactcaagctcccctctgccccagctccaaagacagacccagc
tcaagcccacatgcagcagacctcataatagtctatcttcttgccatttc
tgccatgagagtgctttctgctttcactgatgaggactttttttcagCTCC
TGGGACCTCCACAGTGGACCTTGGGTCAGGGACTCCATCCTCCCTCCCCA
GCCCCACAAgtaagtaccagccaatggtatctgtattagatcatgcctga
tgaatgcaaacatctgtgccattttcagtcaaatgaaaatggaaaatcat
aataaatctagtgatactgagtgaaccaaaaaaaatgtattggccaccta
cagtgtaccagaccctagggatatagcaaggaaaatagaaccaataaaaa
catctctgccctcagtgagcttgtgttcatgtgatgatatgatggtggtg
gtggtggtaatagtaataatgacatattcagtttgatgataatttatgat
tatggtgttgctgttgatgatggtggtggtgatgttactgacaatgatga
tgacggacatgaggatgttgtcagtgatggttgtgaaggttatgatggta
atgatgtgttgagtgtgatgatgatgatgatggtggtggtgctgttgatg
atggtgaccatggtactgatggtgttgatgatggtagtggtgacgttgat
gacaataacaataatggtgatgaggatgatgccagtgatggtcatgtggt
tattatgataatgatgatgtgttgagtgtggtgatgattatgtttgtggt
gatcatgatggggattatcatggtgatgatgataataatgatggtgatgg
tgacaatgatagcaacaatgattatggccataataaagataacagataac
atcagacaatattgagcactgaatatgcacaactagctatgctcagcatc
taactactattatataatatattttataaaaataaattgtattattata
ggcaagggagacataaatatttttctctcaatttaaattttgtatatgtt
taaagataagtctattccaaacccctatttctctactttgaacatagtg
tttattttcaattcccactacagCTGCTGGCCCTCTCCTGGTGCCGTTCA
CCCTCAACTTCACCATCACCAACCTGAAGTACGAGGAGGACATGCATTGC
CCTGGCTCCAGGAAGTTCAACACCACAGAGAGAGTCCTGCAGAGTCTGgt
tagtgcccttccctcctcactctgccagccccagatatccagtcccttct
acatcatagatgccaaggtgatgaaagaagatagcaccaacctcacccct
gcccctgagagatggaagtcccgcccacagagaccagccctgctcattgg
tgcctactcctcccacctcacatctgcccctaacacacacactccttagc
cctcctactcacctctctctccctcctccacagCTTGGTCCCATGTTCAA
GAACACCAGTGTTGGCCCTCTGTACTCTGGCTGCAGACTGACCTTGCTCA
Ggtgagactttagaagagccagcttggctgccacaattgttctcacttta

aaagactttgcactgtctccttgctgcacttcctagggatatcatcacca
aaaagggaattcaggagtcactggcttgagaaccagttgtttcctgatag
taacaccaccattccccacctcaacaggcagaattttcgtgatccatcat
caggattgaggcactaccctgatcactcctctgtattccctcctttgcct
gcccctccctttcatttagtgttaaattctgtccccaggactgctcttaa
gacaatcatacctcatccacatacatccatccccctttcaagctcttcac
tagtctgaagtctgggttctcctgtccccatgccatgagaatgcaggttt
caccttgcacttttataaaaattattatatccatgactctgcttgcagtc
ccaaccaagatagtggtctatgtactcagataatctaagtgcagattctc
acctctttcccagatttctcatttcctctggttccttgatatgtttccct
cagcaatctcaagacaagtcctaggcaatcttttcattgtcattcccccct
cctaccttcctcagGTCCGAGAAGGATGGAGCAGCCACTGGAGTGGATGC
CATCTGCACCCACCGTCTTGACCCCAAAAGCCCTGGAGTGGACAGGGAGC
AGCTATACTGGGAGCTGAGCCAGCTGACCAATGGCATCAAAGAGCTGGGT
CCCTACACCCTGGACAGAAACAGTCTCTATGTCAATGgtgaggagctgca
atatggtaggaatctcttcctccttgctgggcagcctctaatctctggct
tgggggcacactccctgcctggccattgaaaattttgtcatgtgctctac
atggggtgactaagttctggacttcatggtttcttcatcatcatgaactg
cattcccttggggcactcttccctttgggagaatgctgataggaaatct
taatggccccatcccatgaaactcatttaattgcaccagggtagccctga
accctattgcatctcccacatctccttaacccttacccagtctcctccct
ccttctctacgcagGTTTCACCCATCAGACCTCTGCGCCCAACACCAGCA
gtgagtattcaactcatgtccacatgcccctgatcctatattaagtggag
caggagctggcccctcctcttaagcccataagaactcctcttgagcaaag
gagctaggaaggcagaagttactcaagctcccctctgcccctactccaaa
gacagtcctagctcaagcccacatgcagcagaccttataatagtctaccc
tctttccatttctgccatgagagtgctttctgctttcactgatgaggact
cttttcagCTCCTGGGACCTCCACAGTGGACCTTGGGACCTCAGGGACTC
CATCCTCCCTCCCCAGCCCTACATgtaagtaccagtcaatgacatgtcta
ttagatcatgcctgatgaatatgaacatctgtgccgttttcactcaaatg
aagatggaaaatcatagtaaatctagtgatactgagtgaaccaaaaaaat
gtattggccacctacattgtaccagaccctagggatacagcaaggaaaat
aaaaccaataaaaacatctctgccctcagtgagcttgtgttcatgtgacg
atgatagtgatggtggttatgatagtaataatgacatgttgagtgggatg
ctgttgatgatggtgatgctgttgatgatggtgatgctgttgatgatggt
gatgctgttgatgatggcggtggtgatattactgatgatgatggtgatgg
acttgaggatattgtccgtgatggtcgtgaagattatgatgataatgatg
atgtgttaagtgtgatgatgatgatgactgtggtgatgctgtttaggatg
ctgaccgtggtaccgatgatattgatgttggtcgtggttatgttgatgac
aatgacaatgatggtgatgaggataatgccagtgatggtgtggggttatg
atgatgatgatgtgttgaatgtggtgatgataatgttcgtggtggtcgtg
atgggcattactatggcagtgatggtcataataatgatggtgatggtgac
aatgatagcaaggatgatgatggcaataaagatagtacataacatcagac
aatattgagctctgaatatgcaccacgaggagtgctcagcatctaaatac
tattatataatatattttgtaaaaataaattgtattgttttaggcaagg
gaagcatggtaaatattttgtcactcaatttaaattctgcatatgtttaa
agataagtctattgcaaactcctatttctctactttggacatagtgttt

gtttcccacctccactacagCTGCTGGCCCTCTCCTGGTGCCATTCACCC
TCAACTTCACCATCACCAACCTGCAGTACGAGGAGGACATGCATCACCCA
GGCTCCAGGAAGTTCAACACCACGGAGCGGGTCCTGCAGGGTCTGgttag
tgctccaccctcctcactccgccccaccccagagagtcagtacctcctac
atcatccatgccaggtgatggaacaagatcatacccacctcacccttgcc
ccaagagatgcaagccatgcccattgaaaccagccccactcactgatgcc
tgttctgcccacctgacttctgccctacacacccacacacgcaacttagc
cctcctactcatctccttctccctcctccacagCTTGGTCCCATGTTCAA
GAACACCAGTGTCGGCCTTCTGTACTCTGGCTGCAGACTGACCTTGCTCA
Ggtgagaccttagaagatcaagcttggctgccccacttgttctcactcca
atcgactttgcactgcttccttgctgcacttcctagggatatcctcacca
aaggtggaattcaggagtcactggcttcatgaccaatgtgttttctgata
gtaacacccccaaaccccacctcaacagggagaatctgaatggcccatca
tcaggattgagcctctaccctgatcatccctctgaattccctcctgtccc
tgcacctccctttcctttaggtcttaaattctgtccccaggatttctctc
aagatgatcgtgcctcatccacaggctttcatgcccatttccagctcttc
attggtctcacgtctggtgtctctgtccccatgccatgagaatgcaggtt
ttaacttgcactttttatttatttatttatttatttatttatttatt
ttatttatttatttatttatttatttatttattatttcattgttgaaa
catagtcttactgcacaccaggctggtctcagctcagtgcaacctccgcc
ttccatgttcaagtgattctcctgcctcagcctcccaagtagctgggatt
acaggcacccaccactatactcagcaaattttgtatttttgtagagac
tgggtttcaccatgttgaccaggctagtcttgaactcctgacctcaagtg
atctgcccgccttggcctcccaaagtgctgggattacagacatgaggcac
tgtgccaagccactttgcactttcataaaaattcttgtattcattaccct
gcttgcagttcaaagcaagccagtagtcggtgcattcagagaatctaagt
gtgatttctcacctctttcccagacttctcatttcctctggttccttgca
gtccttccctcagcaatctcaaaacccttcctagttaatcttttgattgc
attcctcccaccttccttccccagGCCTGAGAAGAATGGGGCAGCCACTG
GAATGGATGCCATCTGCAGCCACCGTCTTGACCCCAAAAGCCCTGGACTC
AACAGAGAGCAGCTGTACTGGGAGCTGAGCCAGCTGACCCATGGCATCAA
AGAGCTGGGCCCCTACACCCTGGACAGGAACAGTCTCTATGTCAATGgtg
aacagatgtgatgtggttggagtctcttcctccctgctgagcagactcta
atctctggcttgggggcatactctctgcctggccattgaaaattctgtta
tgtgctctacatgggatgactaagttctggacttcatggtttcttcgtca
tcgtgaactgcattccctcagggcattcttctctgttgtgaggatgccga
taggaaacttttaatggtccctgtcccatggcaccaggatagccatgacc
cctattgcatcccccacatctccttaatccttacacaattttctccctcc
ttctctatgcagGTTTCACCCATCGGAGCTCTGTGGCCCCCACCAGCAgt
gagtattcaactcaagtccacatgcccctgatcctacaccaagcacagca
gaagctgcccctcctcataaacccataagtcctcctcatgagcaaaggag
ctgggaaggctgaagttattgaagctcccttccaccccagctccaaagac
aggcccagctcatgcccacatgcagcagacctcataatagtcccctgttt
ggccatttctgccatgagagtgcttctgctttcactgatgaggacttttc
ctcagCTCCTGGGACCTCCACAGTGGACCTTGGGACCTCAGGGACTCCAT
CCTCCCTCCCCAGCCCCACAAgtaagtaccagtcaatgacatctctatta
cagcatgcctgatgagtgtgaacatctctgccattttcactcaaataaag

atgtaaaattatagtaaatctggtaatagtgagtgaaccaaaaatatttg
ttggccacctactgtgtaccagacccaagggatacagcaaggaaaacaaa
accaataaaaatgtctctgccctcagtgagcttttttattcatgtgatga
tgatagtggtggtggtgctagttgttgatgatgatgatgatcatgatcat
ggtaatgatcatgatggtaatgctgttgatgatggtggtggtgctgttga
tgatgatgatgatgatgatgatagtgatgctgttgatgataatggtgttg
atgttgccgaccatgatgatgatggatatgaagatgatgtcagtgacggt
tatgatgttagtgatgatgatggtggtggtggtggtgctatttatgatga
tgatgatgaaactggtgatgttgatgacaataactggtgatggtggtgag
gatgatgccagagatggtcatggggttatgatgataatagtgatgtgttg
agtgtggtgatgatgatgcttgtggtggtcatggtggggattattatgat
ggtggtgataataaagattgtgattgtgacaatgatagcaatgatgatga
tggcaataataaagataacaggtaacatcagagaatattgagcactgaat
atgaaccaagagctatgctcagcatctaactactattttatataatatcc
tttaaaaaataaattctgtcattgaaggcaagggatgcatggtaaatatt
tattttgtcagtcaattaaatactgcctatatttaaagataactatattg
caaactcctattttctctcctttgaacatagtgtttatttccccctccca
ctacagCAGCTGTTCCTCTCCTGGTGCCGTTCACCCTCAACTTTACCATC
ACCAATCTGCAGTATGGGGAGGACATGCGTCACCCTGGCTCCAGGAAGTT
CAACACCACAGAGAGGGTCCTGCAGGGTCTGgttagtgccctgccctcct
cactctgccctgaccaagatactcagtccctcctacaacatccaagccag
ggtaatgggagaagaatgtacccacttcgtccttgcccccagagatgcaa
gactcacccactgaggccagccacgcccactgaagctagcctcacccact
aggccctgcctcactcaccttatttctgcacccacacacagggaccttag
ccctcctactcatctctctgtccctttccacagCTTGGTCCCTTGTTCA
AGAACTCCAGTGTCGGCCCTCTGTACTCTGGCTGCAGACTGATCTCTCTC
AGgtgaggctagaatagccagcctagctgtcccaattattctcactccaa
aaggctttgcactgcttccttgctgcacttcctagagatatcctcaccaa
aggtgggtttcagcagtcactggcttcaggaccagtgtgtctcctgatag
taacacccccacactccacctcaacagagagaatctgcattgtccatcat
cagaattgagcccctaccctggtcatcactctgaattccctcctttccct
gcccctccctttcatttaggtgttaaattctgtccccaggatttctctca
agacaatcatgcctcagccacgtgctttcatcctcatttccagctcttca
ctggcctcaagtctgggctctcctgtccccatgccatgagaatgcagaat
tcactttgcactttttttttttttgagacagagtttcactcttgttgcc
caggctggagtgcaatcgcacaatctcggctcactgcaacctctgcctcc
caggttcaagcgattctcctgcctcagcctcccgagtagccgggattaca
ggcatgggccaccacacccggctaattttgtatttttagtagagacaggg
tttctccatgttggtcaggctggtcttgaactcctgacctcaggtgatcc
acctgcctcggcctcccaaagtgctgggattacaggcgtgagacaccata
cccagcctcaccttgcacttttatcaaaactgttgtattcatgactctgc
tcacagtctctaacaagccagtagtctgcgcactcagagaatctaagtgt
ggcttctcacctctttcccaggtttctcatatcctctggttccttgccgt
ccttctctcagcaatctcaagacccatcctaggtaatcttttcattgtca
ttctccccacctaccttccccagGTCTGAGAAGGATGGGGCAGCCACTGG
AGTGGATGCCATCTGCACCCACCACCTTAACCCTCAAAGCCCTGGACTGG
ACAGGGAGCAGCTGTACTGGCAGCTGAGCCAGATGACCAATGGCATCAAA

GAGCTGGGCCCCTACACCCTGGACCGGAACAGTCTCTACGTCAATGgtga
gtggctgtgatgtggttgaagtctcttcctccttgctgagcagcagctaa
tctctaactagaggtcacacttcctgcctggcaattgaaaattctgtcac
ttgttttacatgggatgactaagttctggacttcatagtttctttatcac
cgtagattgtgttccctcagggcattctttgctgatatgaggatgctgat
aggaaatcttcaaatgccccgtaccatgaaattcattcctttgcaccagg
gtagccttgacccctatttggttgccaatgtctccttaacccttacgcac
tctcctgcctccttccctatgcagGTTTCACCCATCGGAGCTCTGGGCTC
ACCACCAGCAgtgagtattcaactcatgtccacatgcccctgattctaca
ccaagcggaacaggagctactcctcctcataaacccataggtcctcttct
tcagcaaaggagataagagggcagcagttactcaagctctgctctgcccc
agctccaaagactgacccatctctagccacacaggcagcggaccccatag
tctcccctcttgccatttctgtcatgagagtgcttcctgctttcactgat
gaggactttttctcagCTCCTTGGACTTCCACAGTTGACCTTGGAACCTC
AGGGACTCCATCCCCCGTCCCCAGCCCCACAAgtaagtaccagtcaatgg
catctctgttagagcatgcctgatgtaaacgtctgtgccattttcattca
aataaagatagaaaatcataataagtctagtgatagtgagtgaaccaaaa
aaattaattggccacctacagtgtaccagaccctagggatacagcaagga
aaataaaaccagtaaaaacatctctgccctcagtgagcttctgtttatgt
gatgatgatgatgatgatggtggtggtgatgatgacaataatactactga
tggtggcaatcatggtgatattgacagtgatgatggtgatgatgataatg
gtgatggaggtgatgattgggatgatgatggttgtggtgttaatggtgat
aatgatgatggtggtggtggtgttggggttggtgctgatggtggtgatga
taataatactcatggagacaatcatggtgatgttgaaaatgaaggtggtg
ttaacaacggcactagatagtatctggcactatggaacactgaatatgca
ccaagacctatgctcagcctctaactactatttaatgtgctctctcaaga
acagctatcataatagtcaaaggaggcatgaaaacattatgttttatcac
tcactttaaatcctgttgcatatgtttagaaataagtctattgcaaactc
ttaaattttctctacttttgaacatagtgtttatttcccaccttcactat
agCCACCGGCCCTCTCCTGGTGCCATTCACACTCAACTTCACCATCACTA
ACCTACAGTATGAGGAGAACATGGGTCACCCTGGCTCCAGGAAGTTCAAC
ATCACGGAGAGTGTTCTGCAGGGTCTGgtaagagccccacctatctcact
ctgccctatccaccttacctagtcccacctatgtcacctatatggccatg
gaagatctcacccacctcccctcaccccaagagataaaagccccatacaa
cctactgatgtcagctccaccaactacagccagccccacccacctcactt
ctgccgtgcctctacttcttcagccctgccactcacctccctctccctcc
ccaacagCTCAAGCCCTTGTTCAAGAGCACCAGTGTTGGCCCTCTGTATT
CTGGCTGCAGACTGACCTTGCTCAGgtgagaccttagaatttccagtgtg
gctgccccgattattcctagtctcaatgagtttggacttctttttcctg
tcctctttgtgattattcttatcaaagatggaatccaggaaactggctcc
aggactaacctgtctcctggtattagcagtgcccccgcccccacttccaa
actgactgagagattccagagtccatcaacaggactggacttctgccctg
gccattcctcagaattccctgtttttcccccacctcccttgactttaggt
gttaaactctgtaaccaggatttctctcaagacaaacatgcctcaaccac
ttgttttcatccccaattccagctcttcagctatctcaagtctgagctct
cctgtttccatgctttaagaatgcaggatccaccctgccattttgggaaa
actgttatatccacaactatgctcacagtcccaagcagtccagtacactc

agggaatctaagtattgcttcccacctctgtattagacttttacttcct
ctgtccttcttgtcctacactctgccatctcaagacctctccttaggcaa
ttcatttattgccattcccctcaccacctctccccagGCCTGAGAAGGAT
GGAGTAGCCACCAGAGTGGACGCCATCTGCACCCACCGCCCTGACCCCAA
AATCCCTGGGCTAGACAGACAGCAGCTATACTGGGAGCTGAGCCAGCTGA
CCCACAGCATCACTGAGCTGGGACCCTACACCCTGGATAGGGACAGTCTC
TATGTCAATGgtaagcagctctgatgtgattagagtctctttctcctgcc
tgggcagcctctactctcttacttgaggtcacatgccctgcttggacact
gaaggctaggacacgtctgtatgtggtgattgatgtcagaatttcatgat
ttcttctttatcttgaactgatttcatccttagtagcttcttccctgata
ctcagattctgacagagaatctccaaaggcccctgttccatgaaactcct
ttcattccaccagggtagccctgactttttttgacccccctacctccccc
ttgactcttatccacactcctccctccctctctatgcagGTTTCACCCAG
CGGAGCTCTGTGCCCACCACCAGCAgtgagtattctactgatgttcccat
ggccccaatcttacaacaaacttagcaggagctgacccctattcataagc
ccttatgtcctttccataagggaaggaacatagaggacacaaattattcc
ccttccccactgccccagctaatcagaatcccagctgaagccccacaggc
aaaaatccccatgaatagtccctcctgctggcattacttccatgagagca
cttgctcctttcactgttgagggcttctcctcagCTCCTGGGACTTTCAC
AGTACAGCCGGAAACCTCTGAGACTCCATCATCCCTCCCTGGCCCCACAG
gtaaataccagtcaatggtatttggagcatggttgatgagtgtaaacatc
tctgtttatactctgttagagcatggttgatgagtgtaaacatctctgtc
attattcactcaactaaagatggaaattcatagtaaatgtagtaaccata
ggtcaaccaacccagttcattgagcactgcctctgtatcaggacctggat
atacatcagggaacaaaaaaagaaaaaacactgccctcagtgagcttata
ttattgtgatgatgatgatgatgggggtggcggtgatgatgattgcagtg
gtggtggtggtggtggtgatggtaaggataatgatgattatgctggtggt
aatcatgggaagaatgatggtggtggtggggattacaattgacaataatt
atggggattttgacaataatgataataataatgatgatgatgacggttgt
gttgattgtggtgatgatgttggagtggtgatgataacaatggtgacaat
gatggtaaagatgatgatggttgtttggatgatgacagtaatgttgctgg
ttatgatgctaatgatgatgtgattatggtgatgatgatgatgtttgtgg
tggtgttgatgtggattgtgacattcgtgatggtgaggatgacaatagtg
atggcgatagtgattatgatgacaatgatcgtgatgatgattattcagat
aatgacagtgatgatgattatgatggtaatgatgatatgactatggtgat
aatgatgtttgtggtagtattgatggggattatcgttgtggtgatggtct
tggtgctgatgctgacaataatggtgatgatgatgatgatgatgatgata
atggtgatgacaaaggtaacaggtaatatctgatatttttcagcactgaa
tatgcaccaaaagctgggctcagaatctaacaaatattatgtaatatgct
ttttaaaaaaataatttctatcaagaagggcatggaaacctttaattttg
tcattcattttaaattctgttgcatatgattaaaaataaacttattgcaa
actcttcaagtctattgcaaaagtcgagaaacttgaggagtttgcaatag
acttatttttaactatatgcaacagattaaaaatacaaacaaacacaata
gtgtttgtttccacaccttttactacagCCACTGGCCCTGTCCTGCTGCC
ATTCACCCTCAATTTTACCATCACTAACCTGCAGTATGAGGAGGACATGC
GTCGCCCTGGCTCCAGGAAGTTCAACACCACGGAGAGGGTCCTTCAGGGT
CTGgtgagacccctgtccaacttactctgcccctcccacgttacctggtc

ccaacaacatcacccatgccagggccatggaagaaaatctcatccatctc
ccctagagacacaagccctgcccactggtgccagctgcactcgccacact
tgtgccccaccactatatccccagccctcctagtcacctccctctccctc
ttccacagCTTATGCCCTTGTTCAAGAACACCAGTGTCAGCTCTCTGTAC
TCTGGTTGCAGACTGACCTTGCTCAGgtgagactttagaagagctagcct
agctgccccaattgttcctagtcgcaaagagtttggattgtttttttttt
tcctgccatccctgtgaatgtcctcacaacagatggaattcgggaagcat
tggcttcaaaactaatctgtctcctgataccatcagcaccaccccaatca
actgcgagattctgcagagtccatgaggagaattgtgcttctaccttggt
catccctctgaattccctcctttccacccacctccctttcctccagttgt
taaattctgtccccaggatttctcacaagacaagcatgcctcatccactt
gctttcatccccaattccagctcttcatctgtctcatgtctgagtactct
tgtctccatgccatgagaatgcaggctttactttgcaattttataaaaag
ttcttgcatctatgactctgttcacagtcctgggctggctggcagccggg
acactcagagaatctaaatgtgacttattacctctttcccaggcttctca
tttcctctggccccttcctgtctggccccaggaaatctcagaactcctcc
tgaggcaatttttaaattgttatttgcttcatctgccctccctagGCCTG
AGAAGGATGGGGCAGCCACCAGAGTGGATGCTGTCTGCACCCATCGTCCT
GACCCCAAAAGCCCTGGACTGGACAGAGAGCGGCTGTACTGGAAGCTGAG
CCAGCTGACCCACGGCATCACTGAGCTGGGCCCCTACACCCTGGACAGGC
ACAGTCTCTATGTCAATGgtgagcaactttgatgtggttggagtctcttc
ctccttgctggacagcctctattctctggcctgaggtcacacttcccgtc
tggctattgaaggctcaaccatgttgtctgtatgtggtggctaaggtcaa
aacttcgtggtttcttcttcatctcaaactgaatttaccctcaggatctg
ctttctaaatctgagggtgctgatagagaatcttcaatggcccgtgttct
gggaaattccttctgttgcaccagggtaccccaccactcccttaacactt
acccactctcgtccctccatctcaatgcagGTTTCACCCATCAGAGCTCT
ATGACGACCACCAGAAgtgagtattaactgatgttccagtgctcctgatc
ctacatcataaagggcaggagcttacctctccttctatgcccctatatcc
tcttcatgagggaaggaagagagagcacaagttattcccctttccctctg
gcccagctccagatggagatactgccccacatgcagcaagggccataaat
actcctacctgctggtgtttctgccatgagagggtttgctgctttcacta
atacagcaaaccttctcctcagCTCCTGATACCTCCACAATGCACCTGGC
AACCTCGAGAACTCCAGCCTCCCTGTCTGGACCCATGAgtgagtactatt
cagtgtcatctctattacagcatgcctgaggagtgtcaacatctctgcca
ttttcttttctttctttttttttctttttttgagacatagtcttgctctgtc
acccaggctagaatgcaatggcgtgatctcagcccactgcaacctctgcc
tcctaggttcaagcgattctcctgcctccacctcctgagtagctgggatt
acaggtgcatgccaccatgcccagctaatttttatatttttagtagaggt
ggagcttcaccatattggtcaaactgatctcgaactcctgacctcgtgat
ctgtctgccttggcccccaaagtgctgggattacaggcgtgagccaccg
cacctggcctacaactccaccattttcactcaaataaaaatgaaaagtcc
tagtgaatctagtgatgacaagtcaaccgacaaattttgttgagcgtctg
gcttcttcggggccctagggataaggcagggaacacaaaagaaataaaaa
tctctatcctcaggaaggttatattgttgtgatgatgatgataatgatag
ggatgatggacatgatgacaacaatgatggtgttgctgatgatggtgatg
atgatgatgatggtggtagtggttttgatggtgatgatgatgatagtggt

ggtgatgattatgggaaggatgatagtggtggtggtgatgtgatggttct
gatggcaattgacaatgtgatcatgatgttgacaatgaggataatgatgg
tggtgatgatgataatgatgttggtggtgatggtggttatgatagtaaga
atgatatgataatggtgttagtgaccatgtgctagtggtgattatatgga
tgatgatggttgtggtgctggtggtgtaatgatgatgatgggtggggatc
atagggattatgatggtcatggttttggtgctgatgctggtggtgatgac
aataatgatagcgatggtcacagtgttgctgatgttgatgatgatcgtgg
tgttggcaaaggtagtagataatgtctagtagtatggaatactgaatatg
caccaagagctgtgcttagcatctaactactattatttagtatgctctat
gagaactatgtaccattacagtgaagggaggcgtgaaaacctttttatttt
gtcactttaaatcatgttgcatatgtttagaaacaattctatcacaaatt
cttaagtgttagtctacttttgaacactgtttatttcccatcttcactat
agCCGCCAGCCCTCTCCTGGTGCTATTCACAATTAACTTCACCATCACTA
ACCTGCGGTATGAGGAGAACATGCATCACCCTGGCTCTAGAAAGTTTAAC
ACCACGGAGAGAGTCCTTCAGGGTCTGgtaagagccccacatacctcatt
ctaccccactcaccatgtttagtcctgcccacctcacctattgcagagca
tggaagatctcatctacctcatcttgcccccagatatgcataccccaacc
actgatgccagccccaccaactgttgccagccctgcccacctcccttcta
ccacaccccctatgacttcagtcctcccactcacctccctctccctcctcc
acagCTCAGGCCTGTGTTCAAGAACACCAGTGTTGGCCCTCTGTACTCTG
GCTGCAGACTGACCTTGCTCAGgtgagaactgagaacagccagtctgact
gatctgagcagtttgacctgcttcccttctgcactccctggagatgtccg
cagccaggtggaatccaggaggcagtggctctaagaccaatgtgcttcct
gttcccaccacctcccacctcaactgagagatgcagagcccatcagcagg
actgagcttctaccttggtcatccctctgaattccctcctttcccctacc
tgcctttccacaagtggttcaattctgttcccaggatttctcccaagaaa
aacatgcctcgtccacttgctttcatccccaaacctagctcttcacctgt
ctcaagtatgagttctccttaccccatgctacaagaatgcagtttccact
ttgcaattttataaaaatccttgcatccatgattctgctcatagttgcta
agagtcagtgcactcagagaatggaagtatggcttctcacttctctacca
ggcttctcatttcctctggcccctcctgtcctgccctgtgggatctcag
aacccctccctaggcaatccgtgtattgtctttccccaatcttgccctcc
ccagGCCCAAGAAGGATGGGGCAGCCACCAAAGTGGATGCCATCTGCACC
TACCGCCCTGATCCCAAAAGCCCTGGACTGGACAGAGAGCAGCTATACTG
GGAGCTGAGCCAGCTGACCCACAGCATCACTGAGCTGGGCCCCTACACCC
TGGACAGGGACAGTCTCTATGTCAATGgtgagtagttgtgatgtggttgg
agtctcttcctccttgctgggcagcctctactctctgccttgaggtcacg
ctccctgcctggctattgaatgctcatccatgttgtctgtatgtgatggc
tgaggttggaacttcatggtttctatttcatcttggactgagttcatcct
caggatctgctttctggatctgagggtgctgatagagaatcttcaatggt
tcgtgttctgggaaattccttccattgcaccagggtaccctgacccctat
atagttccccaccactcccttaacccttacccaccctcttccctccctct
ctatgcagGTTTCACACAGCGGAGCTCTGTGCCCACCACTAGCAgtgagt
atccactgatttccagtgctcctgatcctacatcatgcagggcaagaact
gacccctcctcacatgcccctatgtcctctatgagcaaaggagctgggac
agcacaagttactccctttcccttctggcccaagtctcttcagagagaga
cccagctcaagccccacatgcagcaaggtccataaatactcctacctgct

ggcatttctgccatgagagggttcaacactttcactaatgaggccttctc
ctcagTTCCTGGGACCCCCACAGTGGACCTGGGAACATCTGGGACTCCAG
TTTCTAAACCTGGTCCCTCGGgtaagtacaaatcaatcgcatctctgtta
gagcatgcctgatgactgtcaacatctctgccattttcacttaaataaag
ataaaaaatcctagtgaatctacggatgaggagtcatccagcaaacttaa
ttgagtgcctagtttctgcagggctctagggataaggaaggggacacaaa
acagttaaaaatatctgctgcaagaaagcttattttattgtgagggtgat
gggagttggtggtggtgaagttactggagatgatgacaataagaatggtg
atgctagtgatgatgatggtgataaggatgataattatgaagatggtggt
ggtgatgatgatgatggtggtggcaggggttatgatggtgatgctgtgat
gatgattacaatggtggtggtgattatgggaaggatgatagtggtgttgg
tgatggtggtagtcttggtagtgattgacaatagtaatgacgatgttgac
aatgagaataatgatgatgatggtagtggtggtggttatgatggtaatca
tgtgatgatgatcaggacagttgtgggggtgattatgggaagaatgatgg
tggtaaggatggtggcggttgtggtagtgattgacaatagtgattgtgat
ggttacaatgaagataatgatgattataatgatgatggttgtgatggtgg
ttgtggtgatgagaataatgatgatacgacaatggtgttggtaatcatgg
tgctggtgatcatgatgcaaatgatgatgattgtggtggtggtgatgatg
atgatgatggtagtggggatgctaccacactgttggtcatgattttggtg
ctgatgtgggtggtgctgatgctggtggtgatgacaataatagtgagggt
cacagtgttggtgacattgatgatgacaatggtaataataaaggtaacag
ataatatttagtattatagaactctgaatatgcaccaagaggtatgctaa
ctactactattatttagtaggctttgttagaaacttctgttgttatagtc
aagggacgcatggaaactttttatattattctctctttaaatcctgttgc
atatgtttagaagtaggccttttggaaatatataaagttctccacttttg
aacatgttgtttctttcccacctccacgacagCTGCCAGCCCTCTCCTGG
TGCTATTCACTCTCAACTTCACCATCACCAACCTGCGGTATGAGGAGAAC
ATGCAGCACCCTGGCTCCAGGAAGTTCAACACCACGGAGAGGGTCCTTCA
GGGCCTGGgtaagagccccgcccatctcactctgcccagttcaccttgtct
agttccacctgtctcactcatgacagggcatggaagaagatctcacccac
ctcctctcacacccagagccgtgagcccccatccactgatgccaggcccg
cctactggtgccagccccaatgactagtcccctgacgctaccccctcagc
catcctactcacctccctctccctctcccacagCTCAGGTCCCTGTTCAA
GAGCACCAGTGTTGGCCCTCTGTACTCTGGCTGCAGACTGACTTTGCTCA
Ggtgagaccttagaattgccaacctgtctgccccaattgttcccagtctc
catgagtgtggacttctttttcttgccctctctgtggttattctgatca
aagatggaactcaaggaacagtggctccaagactaacctgtctcccaata
ttaccactgccccctgccatcacacccaaattgactgagagtttccacag
ggtccatcagcaggattggacttccaccctggccatccctctgaattccc
tcttttctccccacctcccttgcccttaggtgttaaattctctaactaag
atttctctcaagacaaatgtgcctcattcacttgtttaattcccaattcc
agcttgtcacctgtctcaagtctaggctgtcctgtccccatgccatgaga
atgcaagaaccacactgaaatgttagaaaaattcttttatccacaagtat
gctcaccgtcccaagctggacagtagtcagtgcactcagagaatctaagt
gtggcttctcatctgtgtaccaggcttctcatttcctgtggccccttctt
gtccttccctcagcaatcttgggactcctccctagacaaaactttattat
tattcccctcacctgccctctccagGCCTGAAAAGGATGGGACAGCCACT

GGAGTGGATGCCATCTGCACCCACCACCCTGACCCCAAAAGCCCTAGGCT
GGACAGAGAGCAGCTGTATTGGGAGCTGAGCCAGCTGACCCACAATATCA
CTGAGCTGGGCCCCTATGCCCTGGACAACGACAGCCTCTTTGTCAATGgt
gagcaattgtgatgtggttggagtttcttcttccttgctgagcaggcctc
tactctctgtcttgaggtcactctccctgcctggccactggtcttggcca
tgttgtctgtatttgatgattgatatgaacttcaccgtttcttcttcatc
ttgtactggagaccttcatcctcaggaccttcttccctgatctgagtgta
cttgtatagaatcctcaaagcccatgttccctgaaactccttcaattgca
ccatggtagcactgaccccttttggtcccccacctttcccttaacccttt
cccactctcctccctccctctctatgcagGTTTCACTCATCGGAGCTCTG
TGTCCACCACCAGCAgtgagtattctactaatgttccagtaaccccagtc
ctactccaagcaggacaggagctgaccccttctcctatgcccctatatc
ttcttcatgagggaaagatctgagagggcacaagtgattccctttccctc
tggcccagctccagagagacactaatctcaagccccacatgcagaaatat
tcctatctgctggcatttctgccatgaaagagcttatgctgctttaacca
atgcgggcttctcctcagCTCCTGGGACCCCCACAGTGTATCTGGGAGCA
TCTAAGACTCCAGCCTCGATATTTGGCCCTTCAGgtaagtaccagtcaat
ggcacctctattagagtatgcatgatgagtgtcaacatctctgtcctttt
cactcaaataagattaaaaatcatagcaaatttactgatgatgagtcacc
caacaaacttctttgagtacccactctctgccaggccctagagataaggc
agggaacacaaaagaggtaaaaatctctgccctcagagagcttcttttat
tttgaggatgatgtgggatagtggtgatgatgatgttgctggagatgatt
acaataatgatggtgatgcttatgaccatgatgtgatgatgatggtgatt
atgaagatgatgatgatgatattgatgatggtagtggttttgacagtaat
gatgatgtgatgatgatgatgatagtggtggtggtgattatgggaaggat
gacagtggtggtggtgatggtggtggttgtggtggtgattgacaatgtgg
tggtgatattgacaatgaggatgatgatgatagtggtggtggttatgatg
gttaaggatgatgtgatgatggtgttggtgatcacggtactagtggtggt
gatgtggaccgtcatggttgtggttgtggtggtgatggtggtgatcatga
tgataatgaggatgatggtggtgattgtcatgatggtaaggatgaaacag
tgatggtgttggtgaccatgttcctggtggtgatggtgcaggtgatgatg
tggatgatgatggtgatggtggtggagatgatagggattatgaagatcat
ggtttttgtcctgatggtggtggtgatgacaataatgaaaatgatggtca
cagtgttggtgatgatgatggtggtgataacaaaggtaatagatagtgtc
tagtattatggaacacagaacatcaccaaaggttatgctcagcatctaac
tattattatttagcatgctctatgaaaaactttgatcgttatagtcaagg
gaggcatgaaaaccttctattttatcactctctttaaatctggttgcata
tgtttagaaataaatctattacaaactcttaaatgttctctacttttgaa
catagtgtttatttcccacctccactacagCTGCCAGCCATCTCCTGATA
CTATTCACCCTCAACTTCACCATCACTAACCTGCGGTATGAGGAGAACAT
GTGGCCTGGCTCCAGGAAGTTCAACACTACAGAGAGGGTCCTTCAGGGCC
TGgtgagagccctgcccacctcactctgccctgcccaccttgtcttgttc
cacctacgtcacccattccaaggcatggaagaagatctcacccacctccc
ctcacctgagagatagccccgccccctgattacagccccttccaccttac
atcttcctcacttctatgtcctcagccatcttactcacctccctcttcct
cctccacagCTAAGGCCCTTGTTCAAGAACACCAGTGTTGGCCCTCTGTA
CTCTGGCTGCAGGCTGACCTTGCTCAGgtgagaactgagaataaccagtc

tggctaccccaagtgttcccaggcccaaggagtttcatcagctttcttcc
ttccctccctatggaagtcctcagcacaagtggaattcaggcgttggtgg
ctccaggatgaacatatctgctgatcctaccacctcccccatcaatcgag
agaatttgcagggcccatcagccagatcaggcttctactttggtcatcct
tctgaatttcttacttctccctacctccctctccttcaggtgttaaattc
tcttccaaggtttctctcaagataaacatcccccatccacttgctttcat
ccccaattccagctcttaatatttctcaagtctgggctctcctgtcccca
taccatgagaatgcaattttataaaattcttgtattcctgactctactca
cattcccaggctgcctggaagttggtgcattcagagaatcttagtatggc
ttctcacctgtctaccaggattctcatttcctctgtccccttcctgtcct
gcccccaggaatctcaggatgcctccccataggcaatctatttaatgtca
tccccccttatctgccctccctagGCCAGAGAAAGATGGGGAAGCCACCGG
AGTGGATGCCATCTGCACCCACCGCCCTGACCCCACAGGCCCTGGGCTGG
ACAGAGAGCAGCTGTATTTGGAGCTGAGCCAGCTGACCCACAGCATCACT
GAGCTGGGCCCCTACACACTGGACAGGGACAGTCTCTATGTCAATGgtga
gcggctgtgatgtggttggagattcttcctctttgctggacagcttctta
ctctctgacttgaggtcacactccctgactggccattgacgtcttggcta
tgttgtctgtatgtgatgactgatgtctgaacttcatagtttcttcatct
tggactgagttcatcctcagtaccttcttccctgatctgagggtactgat
agagaatcttcaaaggcccctgttccttgaaacttcttccattccactag
ggtatctgtgaccccctatttgattccccacctctcccttaacccttaccc
actctcctccctccttctctgtgcagGTTTCACCCATCGGAGCTCTGTAC
CCACCACCAGCAgtgagtattcaaccgatgctccagtagccccaattata
caccaagcagggcaggagctgtcctgtcttcctatgcccctatgtcctct
tcataaaggaaggggctgggagggcacaagttattccctttcccttctgg
ccagctccagagagagacccagctcaggcccgatatgcagcaaggcctgt
aaatagttttatttgctgacctttctgccatgagaggcttggatgcttcc
cctgaagagggtttctctgtagctcttgggactaccacagtggacctggg
aaactctggggatccaccccttctactggtcccttgaataagtaccagcc
aatggcacctctgttagagcatggctgatgagtgtaaacatctcttccat
tattcagtcaaataaagatggaaattctttataaatctagtgatgatgag
ccaaccaacaaactttattgagcattgtgacaagccctggggctctgcca
aatcctggggatatggcatggatcatgaaacaattaataatctctcctct
cagagagctatttttatgatgatactgatggtggcaatgatgatgatgtt
gatggtgattatgaccatgatgacaatggtgatggtggtggtgatgatgg
taatgatgatgatggtgatgttggtaatgatggtggtgattatgacaata
atgatggtgatggtgacagggatggtgatgattatgatggtggtggtgat
aacaaagttaatggataatatatgaacttattggctactgaatatgcacc
aaagtgctatgctcagtgtttaactagtactatttaatatgatttctaaa
aaaaatcttgaattattataggcagaagaatcatgggaacctttttatttt
gtcactcactttaagtcctattgcatattttttaagtcaattgcaaacac
agtttctctgctttgaacattgtgtttatatccagtcaccccaatagtgc
ataaacctgctgattggagcaactgtgtcttactcccttgtgcttcccta
gtatctgcttcaggaccttgtacatggtagatcgacagatttagatctac
aggaaaatatggattttcccagggaaggaaggaatgaagtatgctttctt
ataatgtatggaaactttcctcttctgccttggttcaactttagtgtctg
ccagagtttacactggaaaactatatggcatctgctccactccctcatcc

atgacagacatcattaattgattgcagcattcatggcagacatcaccaat
tgataatagcattcattttctctcagttcaaaacagcttcagaatggtta
ccaaaaaaaaaaaattcagtcgctaccaattcaattggagctgactcagg
attatgggacagaattcaagagagttaggttccttgatgatgtgtagttg
gctctttggttttcctagaaggctcagctcaggctcagcttggtcattgc
tgatatcctttcttccacttggtcgatttggctgttgatacttatgtatg
cttcacgaagttttgtgctgtgtttttcagctccatcggttggtttatg
ttcctctctaaactggttattctagttagcaattcctttaacctttcatc
aaggtgcttagctttgcattgcattagaacatgctcctttagctcatcgt
actttttattgcccatcttctgaagcctacttctgtcaattcatccatc
tgatcctccatccagttctgcacccttaatggagagatgttgcggtcatt
tggaggaaaagaggcactctggccttttgggttttcagcatttttttgtt
gattatttcccatcttcaggagttttagtttcaggctttgaggctgctga
tccttggatggggtttttatgggggtcttttggttgttgttgttgatgat
gatgatgttattgtcactttctgcttgtttttctttcaatagtcaggtcc
ctcttctgtagggctgctgcagtttgctaggggttcacttcaggccctat
tcatctgattcgctcccatgtctggaggtgtcactcaaggaggcttggag
agcagcgaacataggtgcctgcttcttctgggacctctgacctcgaggga
caccaacctgatgccagtaggatcgctcctgtgtagggtgtctgacaact
attgttggagggtttcgcccagttgactggcatggagagcaggacccatt
taatgaagcactttgtcccctggtggagaggggttcttcactgggggga
aaccacatgtctgggctgcttggattcctcagaactaccagaggagaggc
taagtctgctggtccacagagactacagccatccctcccactagggcccc
aagcccagggagtccaaattctgtctctgagcctctggctggagtctttg
gagatcctgcaaggaagctctgcccactgaggaaggatgggtcagggtta
gccctgaagaggcactctggctgcagactgccacagccggtgtgttgggc
tgtggggacaagtcttgggaccaagccgtccagcctacccggctctagca
ggggaaaagtacagcctggagctattgaaaggggtgccgcccttcccccg
cccagggagcttagcgtgttaggcagttgtgagtccagtgctggctgtcg
ccccttccccaaggaacaaaaaagacttagcaggcagccgcagccagtgc
tggtcgcccctcccccggggagttccgtaggcttaggcagattccagctg
taagaatctgcgtgttctggggttgggacactaggtcccagtggcatggg
ttcgcgagtgagatcttccaatctgtgagttgcacagttccgtggaaaaa
gcacagtttccccctcttgggtagcccgctcactcaccacctcccttggc
tggaaggagggggttccccttccccgtgtgtctctcaggtgggccaccac
accacactgctcttccttctctctgtgggtcactgccagccttctagtca
attttgatgagggaacctggacattttggttgccaggaaggatcacacac
ttattacagtttttttcaatgtgagcctctgagcgctgctgcttatagtc
gaccatcttggcccccagagtcacacatctgttatttttgatgttttga
ttgtggcaattcttgcagaagtaaggtggtatcaccttatggttttgatt
tccctggtcattagtgatgttgaacatttttttcatatgttcattagcca
tttgtatatattctttcaacaactgtctatttatgtccttagcccacttt
ttgatgggattgtttttttcttgccaatttgtttgagttcgttgtagatt
ctagatattagtcctttgttggatatatagattgtgaagatttctccca
ctctgtgggttgtctgtttactctactgactgtgaaggaaaagtcaattt
cttatacgaatttgtctcactcctacttccaaatgagatcctggggtttt
ttttttctgttaatccttcacaatacttctcccactttttttgaactcatt

tgtttatattctgttgtctgcttctctttataggaatgtgacttcttat
gggctttctctattataccacatatgggtttttgttttgttttgttttgt
tttgttttgtttttgtcctcggatccattctccaacctcctccagccttc
ccgtgctctgtgggatagacgtctgactcatgaaaactacatttcccagg
ctcccatgctaactagcttcctgttaggttcagccaataggaggcattgg
tgggacaatggtgggcggggctatggaagggccagagtatttctgtaccc
cgccccccctgctccccttccaatgttcctggagcggtgtaggaccaatac
tgtatatatggaaggaaggcaaggtggatagatggaaggaagaagtgaca
gatggaaagaagaagtgataaatggaaggaggaagggagagaggatggat
gagtggattgaagaaagaaaaaaatggatgaaagaaaaaaggaggaggaa
agatggataagtagatggaaggaagaaaagagaggtgtaagaaaggaaag
attgatgatggatggatgaatggatcagtgggtgagtgggtgaagggatg
aatggatggatggacagatggatgaacagatgggtgggtggatagatgga
tggatggataaatgggtaggtggatggatggatggatggacagatgggtg
ggtaggtggatggatggatagatggatggataagtgaatggatggatgga
tggatggatggataaatggatggatgggtgaaaggaaggaaagaagtgag
agaaggaagaggaaggatagacagatgttagaaggtacaatgaaaggaag
gaagccagcaagaaagaaaggatgcattaatagaatgaaagatggaaggg
aagaagaaaggatggaaagagagaaggaagaatgaacagaaggaagttca
agagtggtgaaaagaagaaaggcagggagagaaggagaagtaaactttc
ttctagagatttgtcttaaaccttagcttggctggacactgtggttcacg
cctgtaatcccagcactttgggaggccgaggcgggtggatcatgaggtca
ggagatcaagaccatcctggctaacacggtgaaaccctgtctctactaaa
aatacaaaaaaaatttagtcaggtgtggtggtgcatgcctgtggtcccag
ctactcaggaggctgaggcaggagaatggcataaaacctgggaggcagag
cttgcagtgagccaagatcacaccactgcactctagcctgggcgacaaag
tgagactctgtctcaaacaaaaacaaaacaaaaaaacaaaaacaaaaaac
aaaaccaaaccaaaacaaaaaaaaaaaaccttaactcatactttcataaag
ttccacacacagggagtgattagaaagcatttgctgatatattttatata
ataaacatgtacaccatattgacctgtgtgcccagcagtgcttacatgat
ttacaatgattaacttgtttaagcttcataacaacggttgaggcaggaaa
catcattgtgaaccattgtcatctcattttacagatgagtaaactgaagt
gctgagaggttggttatggctgcaaagattgttggccatgttaaccaatg
catagaagattagcatacctggttgtgagtgcaggagagagagagaaatg
ggagaaaggcagagaaggatcgatggggagagaggaagagagagagagag
aataaatttttaaaaatgtctagagtcatgacttccgcatcagtgtggt
aatatgcagcctttaccctgggaaagatcagaaccattggtactttttac
agaatcttcccttcctgcatttgggtagaaggaccccatctggacatccc
aaatcattaagcacacccttactggctgctggagttgtctccattaaaag
tcaccgttgggtttattaagaggcggacacagggtccttagaacacactg
cccccaccctgtcccacaccaccccccacccacccatcatcctccccaag
agcttcatctctctctctcttccccctgccctagCCGGGGTGGTCAGCGA
GGAGCCATTCACACTGAACTTCACCATCAACAACCTGCGCTACATGGCGG
ACATGGGCCAACCCGGCTCCCTCAAGTTCAACATCACAGACAACGTCATG
CAGCACCTGgtgagaggcctgcctcccgctgcagccctgccatgcccatc
ctagggctgttgcctgcctgcctctgaccaacccaagctcccttctccct
ctgcagCTCAGTCCTTTGTTCCAGAGGAGCAGCCTGGGTGCACGGTACAC

AGGCTGCAGGGTCATCGCACTAAGgtgagaaactcccccacccacagcgc
accaccaagaacttagagttctgactgggaggtccctcttgggttggggt
gggctacatattttttaaatcttttatctttcctttttttttttttga
gatgaagtttcgctctcgttgcccaggctagagtgcaatggcacgatctt
ggctcactgcaacctctgcctcccgggttcaagtgattatcctgcctcag
cctccccagtagctgggattacaggcaggcaccaccatgcctggctaatt
gttttgtattttagtagagatggggtgtctccatgttgatcaggctggt
cttgaactcctgacttcaggtgatccaccctcctcagcctcccaaagtgc
tgggattacaggcgtgagccaccatatctggccccattcttttttttaa
atgaatttaaggagtgcaaatgcagttttgttacatgcatatattccat
agtgaagtctgcagacagtagacttccagacagtagcttctggtgtatca
cccgaatagtgtacattgtacttattaagtgaggttccccacccttctcc
cactctcccacctttctgagtatccagtgtctattattccacactccagg
tccatgctctcacgtataagtgagaacgtatggtattccaccatgagcta
atggacatggagtccattggctcccacttataagtgagagcatgcggtat
ttgactatttctgagtttcacttaagataatggactcccattccatccat
gttgctgcaaaatacatgatttcactcttttatggctgaatagtatttc
gtggtatatatatataccacattttctttatccagtcttctactgatgga
cacttaggttgggtccatacctttgctgtgaatagtgctgcaataaacat
acacgtgcaggtgtctttttatataatgatttctttttttctttctttt
tttttgatataacgatttcttttattgggtagatccccaatagtgggatt
gctgggtcaaatggtagttctacattttgttctttgagaaatcctcata
ccattttccatagattgtactaatttggattcccaccaacagtgtataag
agttcccttttcttttattcttgccagcatctggttgtttgttgtttgtt
tggttggttggttggtttttgtctttttagtaatagttattctgactggt
attaataactttttatttattcatgacagtttgatttttgacatataaa
aattgcttgcatttaaagtgtacaacttgatattttggtatacattgtta
aatcatggccacatttcagctaattaatatatctattatctctacatagt
tatcatgtttgtaccctttgaccagcatcaccccatttgctcctcctccc
agcccctggcaaccaccatcctactctctgcttctatgagtctgacaatt
ttagattccacctataagttagattatgcggtatttgtctttctgtgcct
ggcttatttcacttagcctaatgtcctccagctccatctatgttatccca
agtggcaggattttcatctttcttatatatttcattgtatatgtgtatgc
cacattttctttacccattcatccattgaaggtcatttagcttgtttcca
tatcttggctattttgaatagtgctgcaatgaacataggagtgcagatat
ctctttaagatactggtttcatttctttctttcttctctttttttttttt
ctgagacagagtctgactctgtcgctcaagctggagtacagtggtgcaat
cttggctcactgcaaactctgcctcctgagttcaagcgattctcgtgcct
caacctcccagggagttttgctcttgctgcccaggctgaagtgcagtggt
gcaatcttcactcaccacaacctgtgcctcccgggttcaagcgattctcg
tgcctcagcctcccaggtagcaaggattacaggcgcccaacaccacacca
ggctaaatttttttgcattttagtagagacggggttttgccatgttggc
caggctggtctcaaattcctggcctcaagtgatccacctgcctcagcctc
ctgaagtgctgggattttacaggcatgaaccaccacacatggcctcattt
cttttagatatatatgggttgagctattctcagagggtccttttctgcat
ctatttaagatcacattttttttatattgtggcaaaaatacatgtaacat
aaaatctgccattttaaccattttaaatgtacaattcagtgacattgat

tatattcacaatgtcatacagccatcaccactatttatttctaatacttt
tccattgggtagatccccaacagtgggattgctgggtcaaatggtagttc
tgattttttttttttgttttttgagaaatctccatactgtttttcatttg
aggttgtactaatttacattcccaccaacagtgtataagagtttcctagg
ccgggcatggtggcttatgcctgtaatcccagcactttgcgaggcccagg
tgggtggatcatgaggtcaggagatcgagaccaccctggctaacatggtg
aaaccccgtctctactaaaaatgcaaaaaattagccgggcgtggtggcgg
gtgcctgtagtcccagctactggagaggctgaggcaggagaatggcatga
accctgaaggcggggcttgcagtgagctgagatcgcaccactgcacactt
caacctaggcgacagagcgagactccatctcaaaaaaaaaaaaaaaaaaa
aaggtttcctttcagtgcatccttgccaacttgagttttctgggttggtt
tgcactctcatggtatttactagatacttctccatttatatttttactca
acccatgcccataacaccactcctctaccattcccaccaaccatgtataa
gagttccttttcttgcatccttgccaacttgacttctttgggtcagtttg
cactctcttggtatttactatttacttctccatttatatttttagtcaac
tgatgcccatggcaccgctcctctgaggcaggtgctgggtactagagtga
taagacagatgctgtccctgccctcacccagtggagaagaacagatgcta
aacaggaacataaatatctaagtaaaatggcttcaaatggagtaaagtga
tatgaaacataaataaatagcaagtgatgggtagagcaactttacccagg
atgaatcttgggctgtgtcccaaatggccatgaaaactgttccaggcagg
gagaacagcatgagaaaaggtcttgaggtgcaaatgagcttggcatgttc
tatgaacagcaaagaggccagtgtggctggagcagagagagagcaagaag
aaaagagagaaaggatgagactcaagacatcagcaagtttgaagggcctt
ggaggacttggatttttttttttaagacagctttgttcttgttgcccagg
catgatctcggctcaccacaacctccgcctcctgggttcaaacgattcct
ctgcctcagcctcccgagtagctggggttacaggcatgtgccaccacacc
tggctaattttgtatttttagtagaaatggggcttctccatgttggtcag
gctggtctcgaactcccgacctcaggtgatccgaccgcctcggcctccca
aagtgctgggattataggtgtgagccactgcacctggcttggattttttt
tttctatattgtggtaacatacacatcacattaaattgatcattttagct
atatttcccgttcagtggcatcaagcacattcacattattgtgcaaccat
caccactatcatccatctccagaactttctcatcttcccaaactgaaact
ccatccccatgaaacactcattcctcatcccccctcctcaagcctctggca
cccaccattctactttctgtctctgtgaatctgatgattctgaggacctc
ctatgaatggaggaatcatatggtatatgtcctggtttatactgtatggc
tggcttatttcaccaagcataatgtcctcaaagttcatccatgttgtagc
atgtgtcagaattcccttccttttccacttgtatgtaaatgctgtattgt
gtttctccattcattaggactttgatttttgcagggagttgtcaaggggt
gctgggttctggggcttcaatataataagagtaagctaaactggttcatt
tcctccttcgtggagaccatgttctggtaggaacaggaacaaataattta
tgattacatagagggtgaccagggcagtgacaggggaagagtggaggatt
gtgggacccagaggaggctcctgaccttgcctaggaagataggaggagga
agaggaggaggaagaggaggaggaagaggaggaggaggaggaggagggag
tcctctaagctgagacctggaggatgaccaggaagttatccaggtaagga
gaaatggggagaagcttccagacaaaagtaacagcaattgcaaagatcct
gagatgatagataaggtcaggtggagaaagtgcaaactgtcaatgagacc
aaaatatggactgtgagttgtgcagtgaccacaagtggagaggtgctagg

tggccttcatcccccaaagctgcacctctccctcctcagGTCTGTGAAGA
ACGGTGCTGAGACACGGGTGGACCTCCTCTGCACCTACCTGCAGCCCCTC
AGCGGCCCAGGTCTGCCTATCAAGCAGGTGTTCCATGAGCTGAGCCAGCA
GACCCATGGCATCACCCGGCTGGGCCCCTACTCTCTGGACAAAGACAGCC
TCTACCTTAACGgtgagcagctatcagccccatctccctgccccaccccc
cagcccccactgcagtccaggagggtgtctgtttgccggttctctaggga
aagacttggggttcaagtcttggcattaccactggccctcccataaccac
aatgcaaggttggactttgattaatcccattttacagatgaagaaactga
ggcttagacaggctaagcaatttaccttgacagtggtggaaccaggatat
gaactccacttgtcagcattcggtgctatgatccactccacatgtttaac
tcacagaagagtcttcctggtgggggcacttggggggacaaaaaacacatt
tccggctgtgagcagtggctcacacctgtaatcccagcactttgggaggc
caaggcgggcggatcacaaggtcaagagattgagaccaccctggacaaca
tagtgaaaccctgtctctactaaaaatacaaaaattagctgggtgtggtg
gcgcacgcctgtagtcccacctactcgggaggctgaggcaggagaatcgc
ttgaacccagaaggcagaggttgcagtgagccaaaattgtgccattgcac
tccagcctggtgacagagcaagactctgtctcaaaacaaaacaaaacaat
ttcccctccctgctttcttctcaccattgacgagggatgggcttctctcc
tacctgaggcccccctataccaggaagatctatgggatctaatcttcaggc
acactgggcctcagcattggtctagaactcaggataagatagcatttaag
aaggcatcccctaaatggggttctgagaggcaaagcatgaccgtggagaa
ttgacaaaatagctcgcctttcatcccctccaccgccaacccaagaacag
tgcttatcatcatgaccccatgaggtgggcaccccatatcacttatatga
ggtacctttaggtaggtaccgggatgtggagagacatcctgggctttcat
tactcttattttagcaaagagggaaactgaggcacagagaagggaaggga
cttgcccatgcccacagcgagtttttggctagtctgggtcttgatgttct
ttattattattattactatcctcctcatgatcatcgttgtatgtatacag
ttatggggtacaagtgcaatttgttacatgcatagatggcacagtagtg
aagtcagagctttcagagtatccaccactccaataacatacattgtatcc
atttagtcctttctcatcatccaccttgctcctacccccctcacccttcca
agccccctttatctatcattcccctctctgagttcttgtctacacattgt
ttagctcccacttatgattgagaacatgtgatatttgtctgtctgtgtct
gacttgtttcacttaaggtaacttcagtttcatccatgttgctgcaaaag
acatggattcattctttttttctaactttttaagttcagacatacacgtgc
agattttctatatggttaaactcatgtcacagggggtttgttgtacagatt
atttcatcaccacccaagtattaagcctagtacccattagttattttttc
tgatcctctccctcctcccaccctccacccgccacccgccaccctccaac
ctcaggcaggccccagtgtgtgttgttcccctctatgtgtccatgtgttc
tcatcatttagctcccacttataagtgagaagaggcagtatttggttttc
tgttcctgcattagtttggtaaggataatgacctccagctccaaccatgt
tcctgcaacggacatgatctcattctttttttatagctgcatagtattcca
tggtgtatatgttcctcattttctttatccagtctatccttgatgggcat
ttaagtagattccatgtctttgctattgtgaatagtgcttcagtgaacag
gtgtctttatgatagaaaaatttatatgcctttgggcatatatgcagtga
tgagattgctgggtcagacggtagttctgtttttagctctttgaggaatc
atcctgctgctttctacagtggatgaactaatttacactcccaccaacag
tgtataaacactccttttttatctgcaacctcagcagcatggttttatttc

tctttatggctgaatagtgttccattgtgcatatataccacactttcttt
atggattcatctgctgatggacatataggttgattccacatctctgctat
tgtgaatagtgctgtgataaacacacaggtgcgggttgggtcttgatgat
ctcagttaacatccagtcccttcaacttggctattgcagggagctgttcc
cccttgtaaactgcacagcttatgtgcttcattttgttccttcatttaga
tttaccaagcagctactattaaccaggccacaatgtgcctcgccccagga
acagagataggttacatgtgcatcctgtcctaatgtaatctccagggggg
cggagactgttttgttctaccctatattccccaaatgtaaagggagcctt
gcacatactaagcccttaataaacattcattgggtggaggaatagattgg
aggaggcctggaaggggaggcgggggttatggatggataggaggatagac
ttgtgaacacaaaggtagtgagagcctctcattggaggcatgctggagac
gtgagtagggaagggtcagtgctaattgaaatatcaggaaattctttcta
gtggtgaacacatttaagtcaaatattagatgatacataaatgtatccat
aatctctagatacacaaagggaaaggcatccaggcaggggccccatatgg
acaaaggcatggagtatctgggacggttccaccacctcctcttacgtgtg
acttctttgtttcaagGTTACAATGAACCTGGTCCAGATGAGCCTCCTAC
AAgtacgtgtctttgaatctagtgcccatttcaatctccatgggtcttgg
ttcaagcttttctcctcattcatgaaggaaggttgccccaaattcgggct
ggtcccctgaggtggtgaggggcattgtctcagtgggaggaagaatgctg
agtccttggcctgtttttagacctgcagccatagtcttggctttgtgaat
tttccatgtccctctgggttggaggaagaagtttgaacaagcattcccta
cagggatagaggttgaggtcagatgatgaccttgttagtctgtacctcct
gataagaaaatctcctccaagtgccccagcagaggcttcatggtcaagct
gcagactctgctggctactggttttggctaaatttgcccattgcctcatc
cagtgatccactcgtctatctttccagccatccattttctatccttcca
gtcatctctcagacaccacctgtccttccatccatccatccgtccatcca
tttacccatccatccatccaccccatttcctgaccatttacctcctcgt
ccttccttccatctgtccttttatccatctattcatccatcacccatcct
cctgcccattcacctgcttgtccctcctttcttctgtccttctatacatc
catccatccatccatccatccatccacccatccactcatccaccacccac
ccatccttctgcccactcactcgctagcccctccttccttctgtccttcc
atccatccatccacccatcttcctgcccattcacctgcttgtccttcctt
ctatctgtcttttatccatctctccatccatccaccatccatccatccat
ccttctccctattcactggtttgtctttccttctgtccttccaaccatcc
acccatctctccattcattctcctcttcattcacctgtttccttatttct
gtctttccatccatccatctatccagccagccatctctcccccattctc
ctccccattcactcaattgtccttccttccatctgtccttttatccatcc
atccacccatccatccatccatctatccttctccccattcacctgtttgt
ccttctttctgtccttccaaccatccatccatccatcatccatccatcta
tccttttccccattcacctgtttgtccttccttctgtccttccaaccatc
cctccatctctccatccatcctcctgcctattcatctgcttgtctttcct
tccttctgtccttccatccattcatccatctgcccatccacccactcatc
ctcttgcccattcacctgcttgtccttccttccacctgtccttttatcca
tccatccatccatccatcttgctcactcctccactcacacaatcactcct
tccctcagtctcatttatggcccacctgtgaatggttgtcctggcttgga
ccactgatgaagcccaggggagcttctcccactaggtggtgggcttttgt
cctctctgatggactgttccttccacagCTCCCAAGCCAGCCACCACATT

CCTGCCTCCTCTGTCAGAAGCCACAACAGgtatttggggccattttcct
cctcgaagattagaatagcatttcaatcagacacctgccctcgtggagtc
ccagattttatgaaataaatagaccatcataatgtcagatgttttggggt
gagatacctggcatagttgggaaggaggagggctttctggagaaagtttc
acctgaactgagtctttaaggatgactaagagtgattcaggcaaataggg
catgaatagtataactgaaagaggggaatctgtgagcaaagcctcagtgg
ccagaaacagcatagagtatagggagaagtgagagaaatttggtttgcat
gaaacataaagcttaacccagagtggatggataagtgagactgaaaggtc
agcaggagccagattgggaagggccttgaatgccaagtcaagaaatttga
acttaacactgaaggccatagggagctgtggatggtactagagcaggggc
agccatagtgagattgtcatttcagaaagattcttcttgtgttcagtata
gagaatgtcctttagacagggcatccagtgagtctgccaggtgctaatca
gggtgagagaaaataagacctgaactgggataggggaggagagagaggat
atatgtgatgaatattcagtaaagagaattggtgttacttggagggggaga
agacacatagcttctgacttggatggccacactagttaataatgagcgca
gtctgatctagtctcagaccagccctcagttgcagacgtctctcctcccc
tcctgcagCCATGGGGTACCACCTGAAGACCCTCACACTCAACTTCACCA
TCTCCAATCTCCAGTATTCACCAGATATGGGCAAGGGCTCAGCTACATTC
AACTCCACCGAGGGGGTCCTTCAGCACCTGgtgagaccctggtcccagca
gctcctggtgggataaatcctacccccaacctctgttcctcggcttaccc
tcttcctccttcctctcaagCTCAGACCCTTGTTCCAGAAGAGCAGCATG
GGCCCCTTCTACTTGGGTTGCCAACTGATCTCCCTCAGgtgagaccactt
cctggccatttgccagtaacaaccaccccttttgtgaccaccccttcctc
agctttcccctgctcctccctccactgctctttacctgcagaggtctcgg
gacctctctagagtcctcaaatgcctctctccccagGCCTGAGAAGGATG
GGGCAGCCACTGGTGTGGACACCACCTGCACCTACCACCCTGACCCTGTG
GGCCCCGGGCTGGACATACAGCAGCTTTACTGGGAGCTGAGTCAGCTGAC
CCATGGTGTCACCCAACTGGGCTTCTATGTCCTGGACAGGGATAGCCTCT
TCATCAATGgtgagtgtcaggctgaacttggatttacagtgacttttggg
gagttggtttctttgtttttgagatggagtctcactctatcacccaggct
ggagtgcaatggtgcaatcttggctctgcaacagtgattctcctgcctca
gcctcccaagtagctgggatttacaggtgcatgccaccacgctcagctaa
tttttgtatttttagtagagatggggtttcaccatgttgcccaggctggt
ctcgaactcctgacctcaggtgatccacctgccttggcctcccaaagtgc
caggattacaggcatgagccaccatgcccggcccaccatgactattattt
gtccctgttgtatgccctttcctctctaaaaaaaatagcccaaggcctgg
ctgggggacacccttccccaaaccaccaagggagggtctttcccattat
tttgagtaaatagcatgaaattctttgaccaaattaatgtcataaattgt
ttgtctctttctccttcacttttgtttccaacttggttgcggtataacta
tcaaatacaattgtatgtatttaagatgtataatgcagtgatttaatata
tgtgtagcttatgaaatgattaccatgatcaaattagttaacactgcttt
catgtcacatagttaccgtgtgtctgtgtgcgtctgtgtgagttagagag
aaagagaacatttaaggtctaccctcatagaaaatttcaggtttacaata
cagtattattaactataatcatcaagctttatactcgatccccagaactt
attcatcttgtaactaaaagtttgtattttgtgaccaacatctccccatt
ttctctatcaccacccccatgcccccagcccctgataaccatcatgctac
tctctgcttctgtaagtttgacttctgatcccacatataagtgagatcat

gcagtatttgtttctctctatctggtatatttcacttagcataatgaacc
ccccccaggtacatccataatgaatttcaattcaaaacccaagtggctga
gtcgtggcatcctttgggacaggatagcaggtcccttctatataaggatc
ctctgtgtcagtggttattaccaggggacaattctgcacttctgccccac
cccaccccccaactgggagactctaggcaatatccgaaatcattttttggg
tatcacaactcagggagggaaggagggtgcaactggcacctagtgggtcg
gtagcccattttccagtgcacaggagacaaccaccccagggaatgatcca
gccccaaatgccaataatttcaagggtgagaaatcctgttgtacatggtc
tcaaagttcttaggtgggcacaaggctgacatttatcacactttactgta
attacttgttaaattatctgattccccttaccctgtgaactcaacaaaa
ttacggtctattatgagtgccactgtaccctcggttcgcagtacatcagc
acatcatagtatggaaagaatcattgaatgagtgagcaaattaaagattt
gtgtctctgctgtaactcacattcattaattcattcattcagcaaacata
tatgggtggctgttctgccccaagccttgtactgggtctggagatagaag
acacatttttctgtctctgaaaaactcatactcaagttaacaacaaatta
cgggcacaacaaagaccccactgctgttattaacagggtactatgggagc
tgagaggaggagtaaattaaggagggcttcctggaggagggtgttatata
cccggccctgtgccgggacacaataatgaataagacagacttgggcctct
gctgtcctggagctccctctcactgggctcttgaagcgtgagcaggagtt
ttgcaggaaatgaaaaggatgcattcctagaagtgggaactgcatagcac
atgcaggaaagctcagctcagaagaatctgtgtaatattccatttttccc
tctctttggggcaactttctgtctaagagctcctgcaatgcccagcgtgt
ggacctgaaattgattctgacagtaggcaggggactgctgggcaactttg
gctctgcattttgtgatcaacatttccccaccatatgttgccttttcttc
ttctctgtggctccagGCTATGCACCCCAGAATTTATCAATCCGGGGCGA
GTACCAGATAAATTTCCACATTGTCAACTGGAACCTCAGTAATCCAGACC
CCACATCCTCAGAGTACATCACCCTGCTGAGGGACATCCAGGACAAGgtg
gggcatctctcacccctcccgtcttctctgtcctgtgtgcttctctccct
cttctacctgatttctctgttaagtgatcactttaaatgcttcacttcac
tatgtattctgggttctctctcagtttccaaaagtactctcttgactacc
attcccatttcacagatgggcaaactgaggctcagaaaggggcgtggtgt
gcctagggtcatacagtgctttaggaacagagttaggatttgaactctgg
tcccctttgctccaagtcctgtgtttttttccactggcatcagcggcccc
tccacccccaagaggcctccatctcacccactctccctacccatctttct
agGTCACCACACTCTACAAAGGCAGTCAACTACATGACACATTCCGCTTC
TGCCTGGTCACCAACTTGACgtaagttctgaaggtcataagcagtgacca
agcttgtggctgtgtctctgagcacccttgagctagacgtccccagtggg
gtacccattctcccctacatccctgtctagctaatcctaccatctcctcc
cataaatcctcaaggtagggagtgaggattaacctcatggggccaccaac
tcccagcatacaccttctttttttctggacacttgggaaaatataactt
tttgatgtagaactcaaaatattagcccaataataatatttaacatcaac
cagcctcctctcatttaattctcacaacagaatctatgagttgagtgcaa
aaatcatccctattgtgcagatgggaaaactgagggtcagaaaagtgaac
ttcccaagaactgtcaaagttgggatttgaacccaggtctctgatgactg
gatgaaggaatgaagatacctatacttgggaagaggagggtcacaggaca
cagggctgactttgtatatttctaaacttcaaagatttttgtatttcagc
tgggaatatggagaaggttaattggaacaaaaaaatgcaaagaatgaata

agacctagtatttgctagcacaacagggtgactgtagtcaaaaataactt
cactgtacattttaaaatataactaaaggtgtatgcttggattgtttgca
acacaaaggatatatgcttgaggggatggataccccatttaccctgatga
ttattatgcattacatgcttgtatcaaaacatctcatataccccataaat
ataaaaacacctactatgtaccccaaaaaattaaaaacaaataaaatcaa
aagtaggctgggcacagtggctcatgcctgtaatcccagcactttgggag
gccagggcaggtggatctcttgagcccagaagttcaagaccagcgagggc
aacatagggagtcctagtctctacaaaaaatacaaaaattagccaggcat
ggtggcacacacctgtagtcccagccactcaggaagctgaggtgggagga
tcgcctgagcctaggaggctgtactccagcctgggtgacagagcgagact
ctatctcaaaaaataaaataaaataataaaaagtagaaatcaagagggaa
aatgtgggagaaattgggataattttaacaataccttccaccagagtgat
gatgaagaatgcataagtcacttcttagtggtcttgatctataaaaagtg
ttcaataaatatcgattattgttactgttattgcttctagacgtaattcc
tggaagcatttttttttttttttttttgagatggagtcatgctctgttg
ctcaggctggagtgcagtggtatgatctcggctcactacaactgcctcct
gggttcaagcaattctcctgcctcagcccccatgtagcagggactacag
gcatgcgccaccacacccggtgaagttttgtatttttattagagacaggg
ttttgccatgttggtcaggctggtctcgaactcctgacctcaggcaattt
gcctgcctcggcctcccaaagtgctgagattacaggcttgggccactgca
tccagccgaaggcctcccatttgatcagaacccttctctagactgaggg
tgggtgcctctagatcttttgctctttaaagacagcaaccgatgaccctg
ctgatgctgagtactggctgaattcctgtggtctctgtccctagGATGGA
CTCCGTGTTGGTCACTGTCAAGGCATTGTTCTCCTCCAATTTGGACCCCA
GCCTGGTGGAGCAAGTCTTTCTAGATAAGACCCTGAATGCCTCATTCCAT
TGGCTGGGCTCCACCTACCAGTTGGTGGACATCCATGTGACAGgtacaag
gtggggtggctggtttcctaactggaagaggtggggttatgaggaaagat
ggggcttctcggtaccagtggaattggtggaggctctagagagggaaagg
gaggctttctggagacccatgtaggtgacctctggcagtagatcatccaa
cgaggcaggaacagaacaccagccattgcatctaagagaatagctatttt
tacatgtaaaaagaattgtgttgaatgaatgaatcaatagatcatttatt
ttgaatcaatttattgattcattcatttaattaatgaataataaatgatt
cagtacataattgattaattgatgtaattgagaattgatttaattgatta
attgatcaattaaaatgatcaattaaatgaatgaatcagtaaatgaataa
ttcattcattcaataaacaatggaagtaggccgggcatggtggctcacgc
ctgtaataccagtactttgggaggcccaggcaggcagatcacgaggtcag
gagattgagaccatcctggctaacacggtgaaaccctgtctctactaaaa
atacaaaaaaaattagccaggcatggtggtggccacctgtagtcgcagct
actcgggaggctgaggcaggagaatggcgtgaacccgggaggcagagctt
gcagtgagccgagatcgcgccactgcactccagcctgggcgacagatgga
gactctgtctcaaaaataaataaataaataaaaataaaaaataaataaac
aatggaagtaaacacgtactgataacacagtgtgatcattgctatgataa
gggaatttcaggggcctgtgggagccccaaggaggaacacacaaccttgt
cttggaaagttttatgtaggaaggggtgaagaagctgagatctgacagag
aatgggacctagccaggggtaatagatggagaattgtgctccatgcatct
ataacctagaagatagaaagaatatggcatctggccgggtgcggtggctc
acgcctgtagtcccagcactttcagaggctgagatgggtggatcacctga

ggtcaggagttcaagaccagcctgaccaatatgatgaaaccccatctctg
ctaaaaatacaaaaattagccaggcatggtggtgcgtgcctgtaatccca
gccacttgggaggctgagagaggagaactgcttgaactcgggaggcggag
gttgcagtgagccgagattgtgccattgcactcaagcctgggcaaaaaga
gcaaaactgcatttcaaaaaaaaaaaaaagtggcattttggggcaagttta
agaagattggtgtagctggagcatccactttgatactggagaggtgacag
ttgaagccaaagatgtgggcagagactttgttgggcactggaatggcttg
gggaggaacatgacacactcatgagttctgctttagaaagaaaatgaaat
gaattctgctcatcctctgggtgctgtgtgcagaatggagggtgggggga
gagaagagcaaaggcaagaagaccctttaggaacaatgatcattagttag
aagactctgggtttctcagcacctgcaattgctgactacacccccagaga
aacccagtctcttttcccccatgttgtagagaattcttacaatgcttggt
agaaagagaattgaacaggtagatgggtggatggatacaagctggacaga
tggatggaggaagatcctccatccaatatagagctgttacctaaaaccct
ccatcccacctttaaaatcctagctcagccaggcgcggtggctcacacct
gtaatcccagcactttgggaggccaaggcgggtggatcacctgaggtcgg
gggttcgagaccagtctgaccaacatggagaaaccctgtctctattaaaa
atacaaaaaaaaaaaaaagttagccaggcagggtggcgcatgcctgtaat
cccgctactcgggaggctgaggcaggagaatggcttgcacccaggaggtg
gaggttgtggtgagccaagatcacgccattacactccagcctgggcaaag
agagtgaaactgtctcaaaaaacaaaacaaatgaccccccctgccaaaaaa
aaaaaaaaaaaaaaaagaaaagaaaaaaagaaaagcctagctcagctcaca
ctgtcaggaataagtaagctagctggaatcatctctttcttaaaaccctg
ccttgatagtggatttttacatactttttttttaattctagAAATGGAGT
CATCAGTTTATCAACCAACAAGCAGCTCCAGCACCCAGCACTTCTACCTG
AATTTCACCATCACCAACCTACCATATTCCCAGGACAAAGCCCAGCCAGG
CACCACCAATTACCAGAGGAACAAAAGGAATATTGAGGATGCGgtgagaa
gggggtggtatgtccactctgttgccatgcagaaactgacttatgcatac
tgggtagccacagggtgactttttataacaatccacaaagacaggttctt
attcccatttaatacacaagcacagagaggttcagtagctgacccaaggt
cacacagctaagtcataccctagaagagcatgtcctttgatatacatacc
tgggcaagtggttgtcatgacaagaagcaaaatagacggagaagtgtgct
cagtggctgaaaattctctgatgctactggggccaggattctgacctaag
aaacatcgccctgtctttcagCTCAACCAACTCTTCCGAAACAGCAGCAT
CAAGAGTTATTTTTCTGACTGTCAAGTTTCAACATTCAGgtaagttctaa
ctcaggacctaatgactctaggaacttctgctgtcctttaaatagaagtg
tccccaagccatagctttgatggaagagagccctagaaatagagagctgt
taactaaaaactagctttttcctaaagctggagcccaactggcttcaaca
ctcaagagagctggtgtaaatctcagcagacataaaggtacctggtgctg
aggccatggagtctagagtgtagaatctactacattaagacatcagctac
tgaaatcaggacccatggaagacgggggaaggaggggactaaaaccagat
tacttagaatctagcagcctaactgtgcttttcaatgagaggtatcattt
ccaatggtggggggtaccaatgattttttttttttgacaactgccttgag
aacaggctttcctcactaaacaaattctgaatcagaacaaataaagataa
gccctgagaatagggctttttcaaggagctgccaaacagatcaaatagtg
actatgttctgcagattgatgtctggagaactctacagctattttgactg
ctaggcagctggttttcacagatatcatgattctgaggctgccagttttc

aaagttaccgaggatcttgctggatgcagtggcttgcgactgtaatccca
gccctttgggaggccaaggtgggtagatcgcttgagctcaggagtttgag
accagcctgggcaatatggtgaaaacccatctctacaaaaaatacaaaaa
tcagctgagcatagtggcatgtgctgtagtcccagttacttaggaggctg
aggtgggaggatggcttgagcccaggaggcagaggttgcagtgagctgac
attgtgccatgcactctagcctgggcaacagagccaaagcctgtctcaaa
aaaaaaaaaacaaataataataataataaaatactgaggatcttgaaaga
gcactgtggaaataatgcaagttaaaatgccacaaagcttgctcttttta
ctgagatttaacactttccttaactaaacacccctcgaatttttgcaagc
ctttggttcacttctagacttctggaaaaattgatttggactattttggc
caatgttctcattgattttatgggtattcagaagttgttaccccaacatt
ccagaaatgttctccctgtggctattactttatttatttatttatttatt
tatttatttatttatttgagacggagtctccctctgttgcccaggctgga
gtgcagtggcgcaatctcagctcactgcaacctccgcttcccaggttcaa
gcgattctcctgcctcagcctcccaagtagctgggattatggatgtgcac
caccacaccggctaattttgtgtttttagtagagatggggtttcactgt
gttggccaggctggtctcgaactcctgatctcaagtgatccacccgcctt
ggcctcccaaagtgctgggataacaggcatgagccactgtgcctgacctc
cctgtggctatttttaaatgaattaagtggaataaaattagaaattcagt
tcttctcccacgctagctgcattttaagcatttaataacaacatgaagct
actaatggctgcattgtgtagtgcagatgtagaattttttttttgttttt
tgttttgtttttgagatggagtctcgctctgtcaccaggctagagtgcag
tggcgtgatctcgtctcactgcaatctctactccccgattcaagtgattc
tcctgcctcagcctcccaagtagctgggattacaggcacgtgccaccaca
cccagctaatatttgtatggatggtctcaatctcctgacctcgtgatttg
tatggatggtctcgatctgacctcatgatccgcctgcctgggcctcccaa
agtgctgggattacaggcgtgagccactgtgcccggccgacatagaatgt
ttacatcattgcagaaagtttctgcaggaagagcctagaaggagaaagcc
tagaatcatgataaaattgcagatatctttgcttatccctgtccccttcc
agGTCTGTCCCCAACAGGCACCACACCGGGGTGGACTCCCTGTGTAACTT
CTCGCCACTGGCTCGGAGAGTAGACAGAGTTGCCATCTATGAGGAATTTC
TGCGGATGACCCGGAATGGTACCCAGCTGCAGAACTTCACCCTGGACAGG
AGCAGTGTCCTTGTGGATGgtaaagctccctgggtcattgggactgaggt
ggaagctcccacttcctcacctgggtccttccctgggaatctgaaggctt
ggggttgattcgtcatcgagctttctcagactgggagaaagtggcttagt
tctcctaagctttacccatcattgaaggaaagaaaaggacgcccgaggga
tatgggaggcatttgccctcttctggccagctctgtgacctcaggctagt
cacatctcctttctggacttcttatctctctgtacttagcaagccacttg
gtttttggttcccatcttgcctgccctagatggtattgctcctccacccc
caggcagctgcagtgttaaacaattaccctgattagttattgttgttgtg
ttgtttgtttgtttttgagacagggtctcactctgtcacctaggctggag
tgcagtgacatgatctcagctcactgcaacctcaacccctggactcaagc
aatccacccacttcagcctcccaagtaactgggactacagccatgcgcca
ccacacccggataattttttgtattttttctagagatggggttttgcaaca
ttgcccaggctggtcttgaactcctgagctcaagcatgccacctgcttca
gcctcccaaagtgctgggattacaggcaggcaggcaccactgcagctggt
tctggtttttttgtgtttgtttttttctttagaggcagggtctcgctctg

ttaaccagaatggagtacagtggtgcaatcatagctcactgcagtcttga
actcctgggctcaagcgatcctcccacctcagcctcctgagtacctggaa
ctacaggcacgtgtcaccacgccttgctaatttctaaatttttttgtagag
acagggtctcactatgttgcccagactggtctctaattcctggccacaag
tgatcctcctgcctcagcaggtcaatgagggcttccagtttcaagttgta
tgtgattcatcctcaacaaatgtggtaggatggacctattttccaactcc
agagatggcttcaaggtggctcaactttgcatatccaattttacccattc
aaagaatagttatatacattgtaccatgtatcaggaatataacagagagt
aactgtttgctctttcaccactatattccaagaaccccatattctgcctg
gcacataataaacactcaagtcatatttgcagaaggaataactagatttc
atacaaggttcttttcaagtcaaatgcgaataacgttttagacgggacct
tccaatgcctgtgtgcactgtccttgattccgaattattgttgtgcaaga
gagcactgttgatccttcagaatcaacaagcctttcacatgcctgtcaca
ggtttttctttttcttgttttaccaattttgtttgttgtttgtttgttgt
tattgttttgttttgtttttgtttttatttgtttttatttttttcttttt
ttttgagacagagtctcgctctgtcacccaggctggagtgcagtggcacg
atctccgcccactgcaagctccgcctcctgggttcatgccattttcctgc
ctcagcctcctgagtagctgggactacaggcgcctgccaccatgtctggc
taattttttttgtatttttagtagaaacagggtttcaccatgttgaccag
gatggtctcgatctcctgacctcgtgatctgcccacctgggcctcccaaa
gtgctgggattacaggcgtgagccaccacacccagccccaattttttttt
taattaaaattgttgtcagctcacaagctttctaaaaacaggccatggac
ccagcatcgctgtagtttgccaaaccttgccttgaatcagtaccatccaa
tagaactttctgcagtgatagaaaatgtttctatctgtgctattcagcac
aaagccatgtgtgattactaagcttgaagtgtggttaatgtaactgagat
accgaagttttaattttatttaattttaatttaaaaagccacttgtggct
gctccatattgcacactactttttaaaattattatttgtatatatttaag
gggcacaagtacaattttgttgcatggatttatagcccagtggggaagtc
tgggcttttagggtatctattacctgaataatgtacattgtacccattga
gtaatttctcatcatccactctcctccactccccaacccttccaagtttc
cactgtctattattccactctctatgtccatgcctatgcattatttagca
ttgacatgtctatgcattatttagtcaaatacatgtgctatttgacttcc
tgtatctgagttgtttgacttaagataatgaccttcacttgcatccatgt
tgctgcaaaagacatgatttcattctttttttatgcctgggtggtattgca
ttgtgtgtgtgtgtgtgtgtgtgtgtgtagagagagagagagatcacatt
ttctttatacagtcctccattgatgggcacttaggttgattccatatctt
tgctattgtgaatagttttgtgataaacacacaggttcaggtgtcttttt
gacaaaattatttattttcctttgtgtagatacccagtcgtgggattcct
ggatcaaatggtagtttcatttttagttatttgagaaatctccacgtttt
tcatagagattatactaaattacattcccaccaacagtgtgtaacggttc
acttttcttgcatcctttttaacatctgttatttttttgtctttttagtaa
cagccattctgactggcgtaaggtggtatctcatcatggttttaatctgt
atttctctgattattagtaatgtcgagcatttttttcatatgcttgttagc
cattggtatgtcttctacatctttaagaagctggctatgggctgggcgca
gtggctcacacctgtaatcccagcactttgggaggccgaggcaggcggat
cacgaggtcaggagttaaaaaccagcctggccaacatggtaaaaccctgc
ctctactaaaaatacaaaaaattacccaggcatggtggtgcgcctgtaat

cccagctactcaggaagctgaggcaggagaatcacttgaacccaggaggc
ggaggttgcagtgagacgagatcacatcattgcactccagcctgggtgac
agagtgagactctatcttgagaaaaaaaaaaagttggctataacagggtt
gtagaagtagaggaaccagtaacccttctcgccatgcctgatgatggctt
tacatccctgtcttcatggagtttatgctgtcgtgaggaataacaagaac
aggcagttgtcaattataaattatttgatgtgaacctattcatacatggg
tgtggtcatcagggaaggcttcctggaggaaatgacattgaaggtgaatt
ctaaaagatgacgataaaaccaccaagtgaaggagagcttaaatgtgttt
ttaggcagaagaaaaacctttgggtgaaaattttaaaacttagagaggt
cccatcagtttccaactgcgatgatccattctctccaccactgcccttgg
gcccagcccaatttaggtccaccatgcccagaggcatgaatttaacttat
gacactcttgtggtggaataatggctttgggcttatgtagccatgtgtca
tttttttagagatacaaattgaaatatttggggtgagatgtcatggtgtc
tactggcctctaaaacttcagtgaaaacatttactttcactgaaatgtca
ataaatcataaattggatgtatatgttttagttggaggaaatataaacca
ctaaatctaggtgatgcatatttattatactcttctctctgctttttttgt
acgcttgtaaaattgtatttaaaagaataagacacacttggccgggcgcg
gtggctcacgcctgtaatcccagcactttgggagaccgaggtgggtggat
catgaggtcaggagttcaagaccagcctggccaacatggtaaaactccat
cactacatacaaaaattagccaggcattttggcgggcacctgtaatctca
gctacttgggaggctgaagcaggagaattgcttgaacccgggaagcagag
gttgcagtgagccaagatcacgccactgcactctagcctgggcaacagag
caagactccatctccagaaaaaaaaaaaaaaaaaagacacactcacatgca
ccctccatttctttcatttctagGGTATTCTCCCAACAGAAATGAGCCCT
TAACTGGGAATTCTGgtaagtctcaaagaagccccagcccagggtaggga
gggggtagcctgatggtgctttgccttgtccaagagcaccaggcacacag
agtcttggatgaggatcaaaattgccaacccatggcaaagactattgagg
catagtaaagggatagcagggatcctggctttctgggggcccagtttttg
ggggcatcagaggcatgaggtgttgagccactaagctctcttccccaggg
ctgtgcccatcctcaggccacatagggtccaagaaggagccctgggacgt
ggcaggaggtggctcaccccagcccttgtctccccagACCTTCCCTTCTG
GGCTGTCATCCTCATCGGCTTGGCAGGACTCCTGGGAGTCATCACATGCC
TGATCTGCGGTGTCCTGgtgagcaaggaagggttgcttgtcttcttaaca
attgggttgtaagagttcttaatatattataaaaccatactatactatac
acaagtcctttgctggatatatgttttgcaaatattttctcccagttcac
ggagtggctttcctattttctttttataattttatttttaattaattgac
aaataatgaatgcatatatttaggggatacaatgtgatgctttggtatat
gtacaattatggaatgactcaatcaagctaattaatatgtccctcacctc
tcatacttattatttctttgtggtgtgaacattggcaacctatactctta
gcaattttgaaatctacattattattaactatagttactatgttatgcag
atctcaaaaacttcacaacctatatgctgattacaagatattgagagaaa
aagtgattgcaaagagtgtaaataaaataatgtaagagggaaaaatgtaa
caaaattagtcgttagggaaatgtacacggaagtcacaatgagaggccac
ttttcacaagaatggataaaattgaaaagattgactataacaagtgttgg
tgaaaatgtgacagaactggaactctcataaagtgaaagtggaaaatagc
ttggccatttctttgaaaattacacacacctaccgtaagaccctaccatc
ccactactagtaatttatctaagagaaataaaaacatatgtctatatgaa

gacttgtacacaagtaaatgttcataacagctttgtttgtaatagccaaa
ctctgaaaacaaccctaatgtccattaacaaatatatcctgacaatggaa
tattattcagcaacaaaaaggaattattaatacattaataaattatacag
caacatgtataaattgcaaaatagttatgcctagtgaaagaatccagatg
aagaaaagagtacatgccatatgattcccttaatagacaaattctagaaa
atacaaactaatctgtaaggacaggaatcagatcagcggttgcctgggaa
tgaaaatgtgtttgcagtggcagggaaaaaggaattgtaaaagagcagga
agaaagtttttttgttgtttttttttttgtttttcttgagacagagtctt
agtctatcgcccaagctggagtgcaatggcacgatctcagctcattgcaa
cctctgcctctcgggttcaagcgtttttcctgccccagcctcccaagtag
ctgggattacacatgcgcaccaccacactcagctaatttttgtattttta
gtagagacggggttttaccatgttggccaggctggtctcgaactcctgac
ctcaggtgatccacccgccttggcctcccaaagtgctgggattacaggag
tgagccaccatgcctggccaggacgaaagttttggggatgatggatggat
gttccttatgttgattgtggtgacgattcaataagttatgatcagaactt
atcaaaacattcactttaaatgtgtgcagtttatttatgtcagttatgc
ctcagttaagctggacagatgtagaggaggaagggagggagagagggggc
tgagatcaggaccaaaagccagagagaaagagactgagaatgagatgaga
gagaaatggtatttagacagaagacaggcgatagatgattgatagttgac
agatgattggtggatagctgataggtagatgataaatagattattttgat
agataattggtagataataaattgataggtaacagatagttgatagatat
tgataagtagatgataaatacatgattgatggatgacaggtgattgatag
atgattgatggattataaataggagatgattgagaggtgagagataattg
atggttattgattggtagataattgattgacaggttgataaatattgata
gctagatgatagataaatagatcattggtagatatgtgatatattgataa
agaaattcagaggcaaaaggagagagaaatgaaggggatatcggaggggg
aaaaattttttaaaccgagagtgaaacaaggagacagaagaaaagaaag
tggtgaaaagaggaaaagaactgagggagaaattaaatgaaacaatgaag
ggagacagaggaagcataaggcctctggctttggccatattctcacccct
gtggtctcctctccctggacggctgaccagtccattctcacgcctcctcc
tcaccctcatagGTGACCACCCGCCGGCGGAAGAAGGAAGGAGAATACAA
CGTCCAGCAACAGTGCCCAGGCTACTACCAGTCACACCTAGACCTGGAGG
ATCTGCAATGACTGGAACTTGCCGGTGCCTGGGGTGCCTTTCCCCCAGCC
AGGGTCCAAAGAAGCTTGGCTGGGGCAGAAATAAACCATATTGGTCGGA

SPP signaling report (CHIP-seq MACS2 score) for MUC16

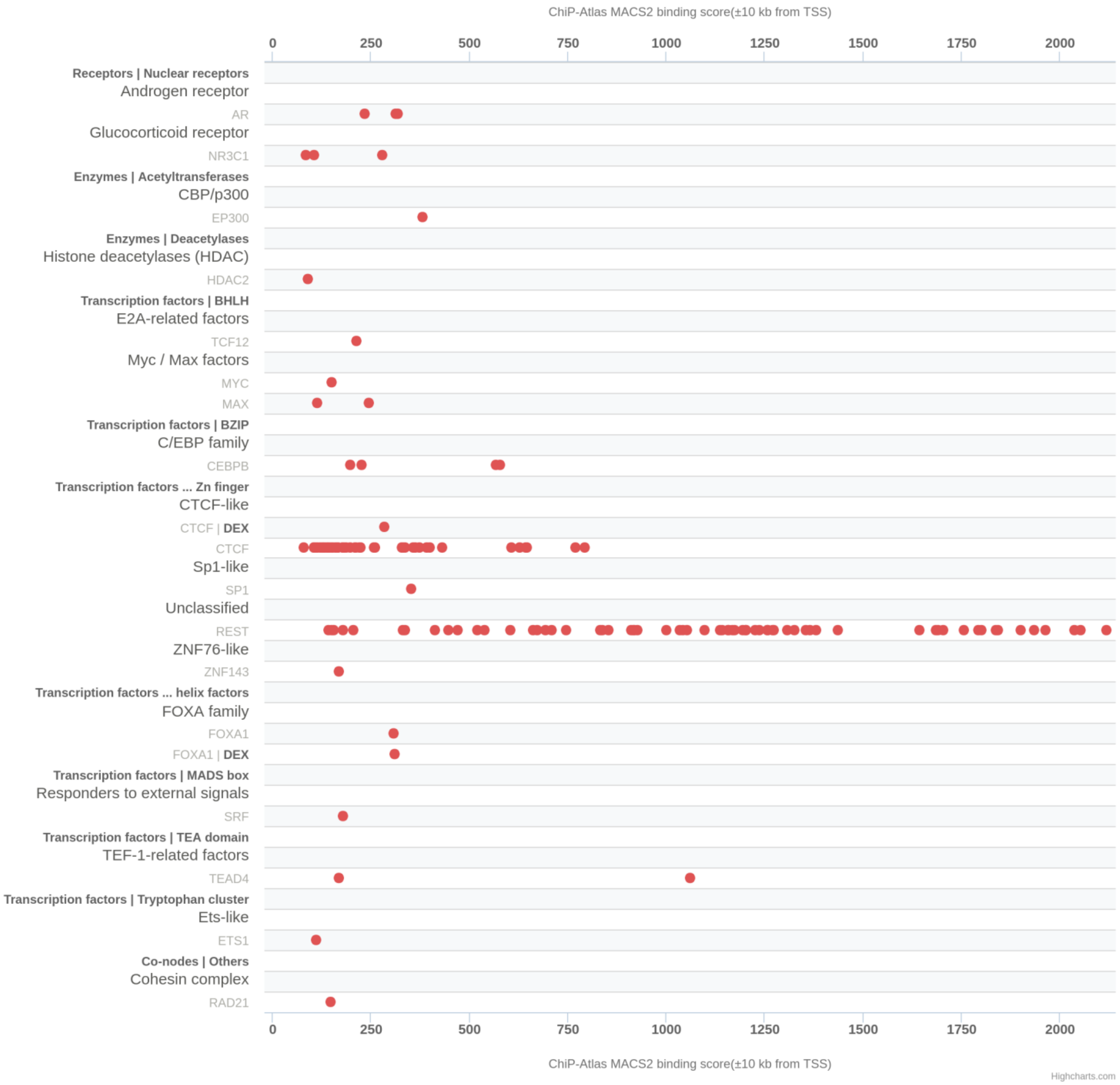

SPP signaling report (CHIP-seq MACS2 score) for TP63

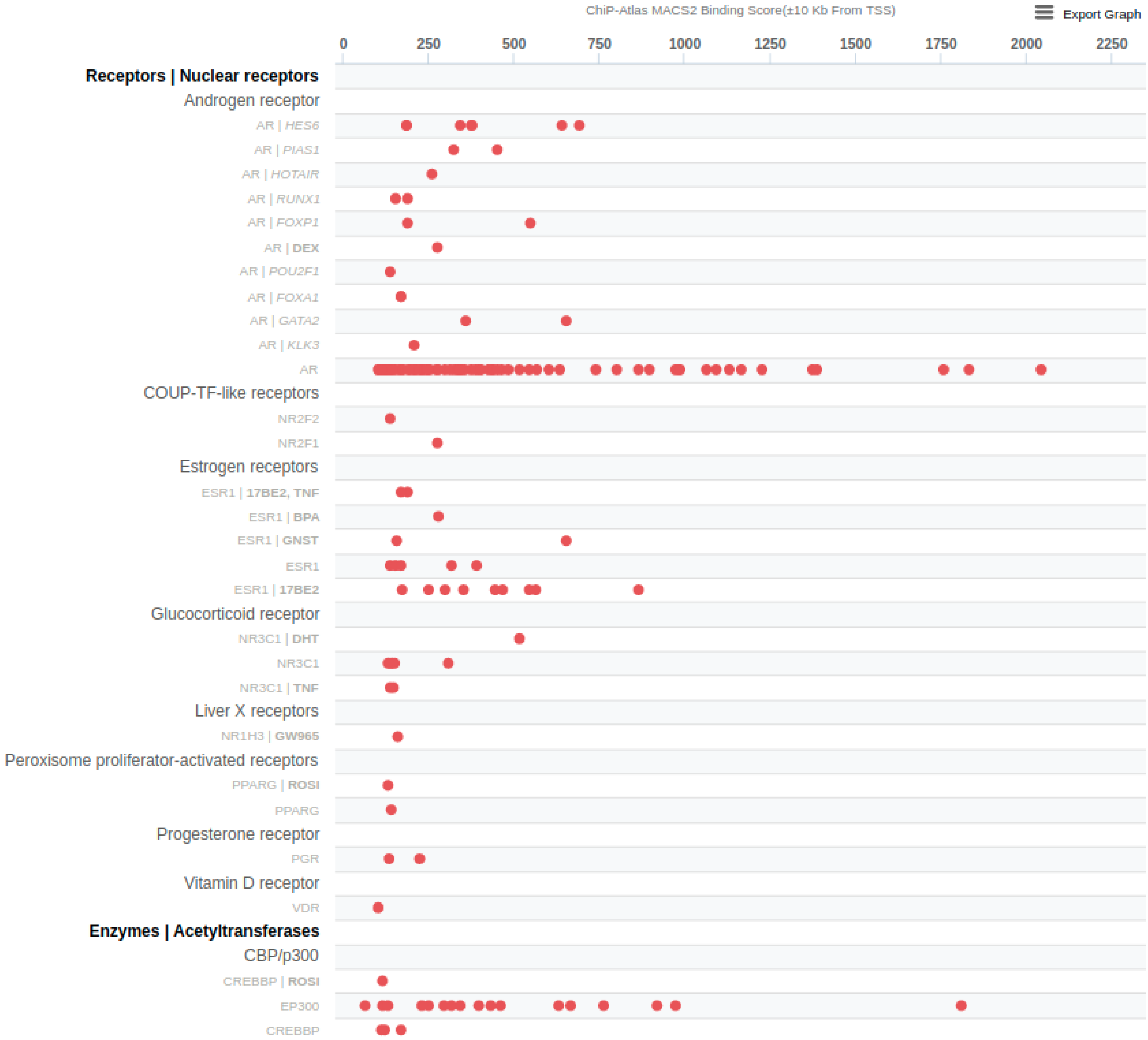
ChiP-Atlas MACS2 Binding Score(±10 Kb From TSS)
Export Graph
0
250
500
750
1000
1250
1500
1750
2000
2250
Receptors | Nuclear receptors
Androgen receptor
AR | HES6
AR | PIAS1
AR | HOTAIR
AR | RUNX1
AR | FOXP1
AR | DEX
AR | POU2F1
AR | FOXA1
AR | GATA2
AR | KLK3
AR
COUP-TF-like receptors
NR2F2
NR2F1
Estrogen receptors
ESR1 | 17BE2, TNF
ESR1 | BPA
ESR1 | GNST
ESR1
ESR1 | 17BE2
Glucocorticoid receptor
NR3C1 | DHT
NR3C1
NR3C1 | TNF
Liver X receptors
NR1H3 | GW965
Peroxisome proliferator-activated receptors
PPARG | ROSI
PPARG
Progesterone receptor
PGR
Vitamin D receptor
VDR
Enzymes | Acetyltransferases
CBP/p300
CREBBP | ROSI
EP300
CREBBP

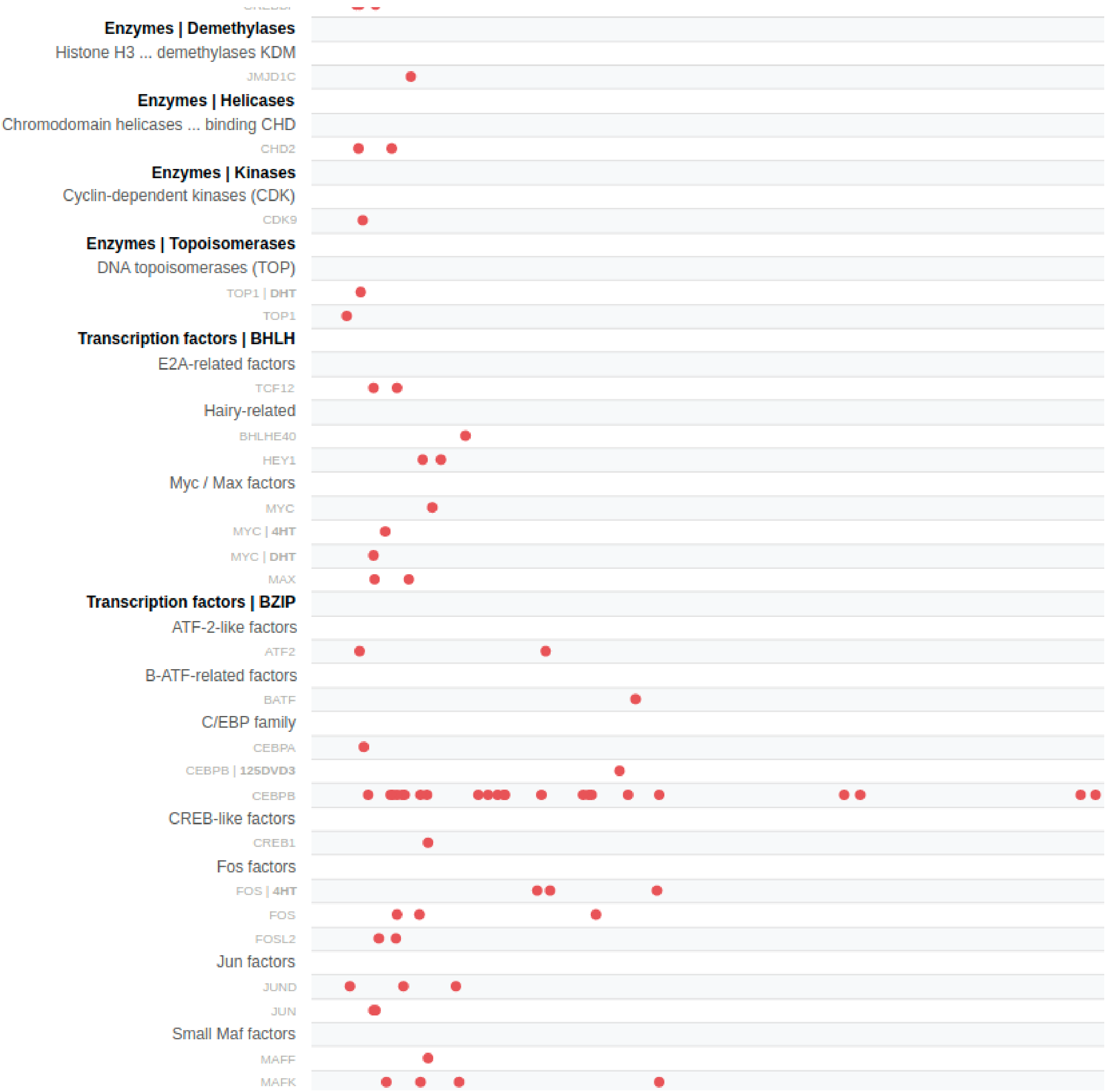
Enzymes | Demethylases
Histone H3 ... demethylases KDM
JMJD1C
Enzymes | Helicases
Chromodomain helicases ... binding CHD
CHD2
Enzymes | Kinases
Cyclin-dependent kinases (CDK)
CDK9
Enzymes | Topoisomerases
DNA topoisomerases (TOP)
TOP1 | DHT
TOP1
Transcription factors | BHLH
E2A-related factors
TCF12
Hairy-related
BHLHE40
HEY1
Myc / Max factors
MYC
MYC | 4HT
MYC | DHT
MAX
Transcription factors | BZIP
ATF-2-like factors
ATF2
B-ATF-related factors
BATF
C/EBP family
CEBPA
CEBPB | 125DVD3
CEBPB
CREB-like factors
CREB1
Fos factors
FOS | 4HT
FOS
FOSL2
Jun factors
JUND
JUN
Small Maf factors
MAFF
MAFK

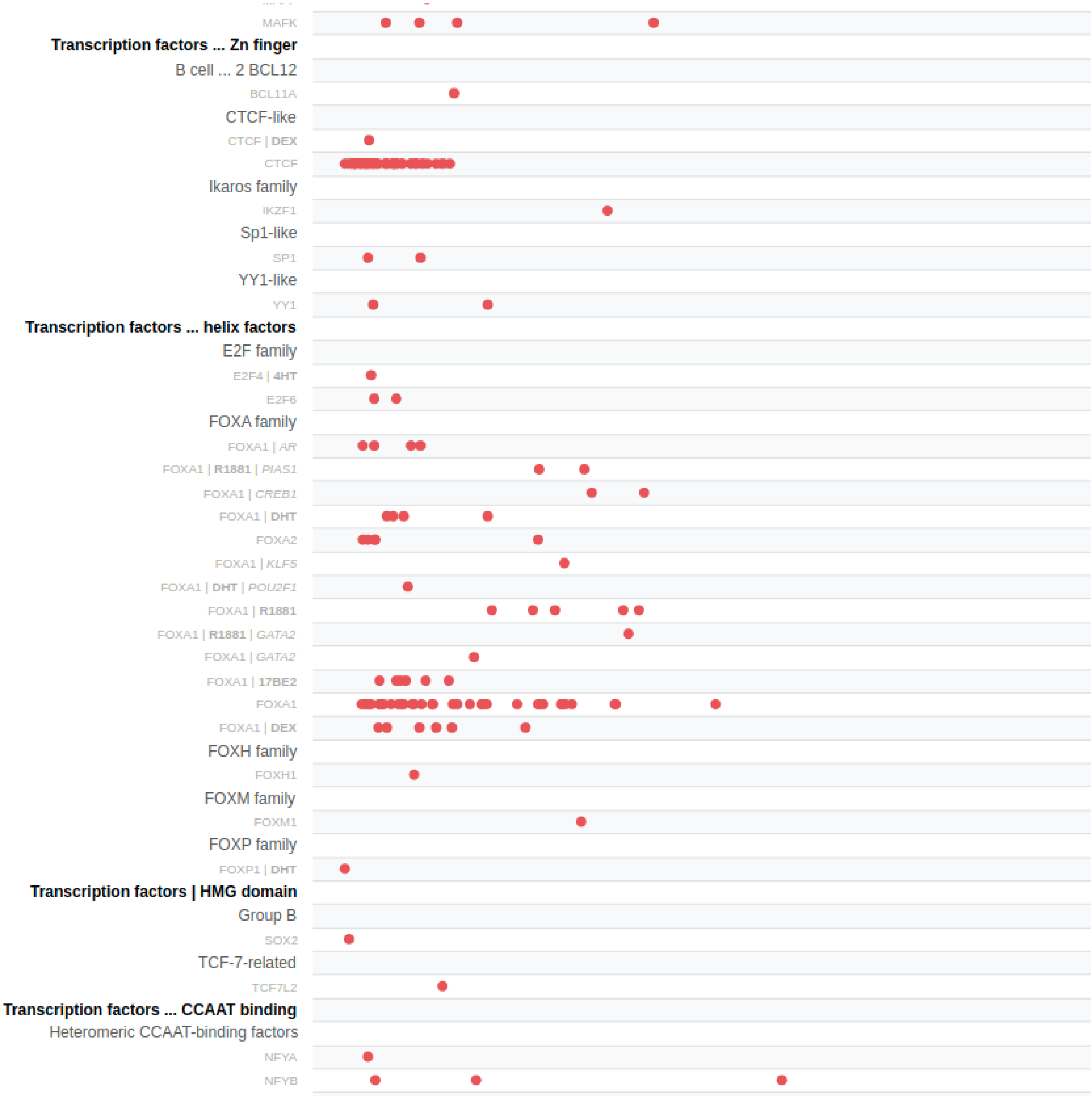
MAFK
Transcription factors ... Zn finger
B cell ... 2 BCL12
BCL11A
CTCF-like
CTCF | DEX
CTCF
Ikaros family
IKZF1
Sp1-like
SP1
YY1-like
YY1
Transcription factors ... helix factors
E2F family
E2F4 | 4HT
E2F6
FOXA family
FOXA1 | AR
FOXA1 | R1881 | PIAS1
FOXA1 | CREB1
FOXA1 | DHT
FOXA2
FOXA1 | KLF5
FOXA1 | DHT | POU2F1
FOXA1 | R1881
FOXA1 | R1881 | GATA2
FOXA1 | GATA2
FOXA1 | 17BE2
FOXA1
FOXA1 | DEX
FOXH family
FOXH1
FOXM family
FOXM1
FOXP family
FOXP1 | DHT
Transcription factors | HMG domain
Group B
SOX2
TCF-7-related
TCF7L2
Transcription factors ... CCAAT binding
Heteromeric CCAAT-binding factors
NFYA
NFYB

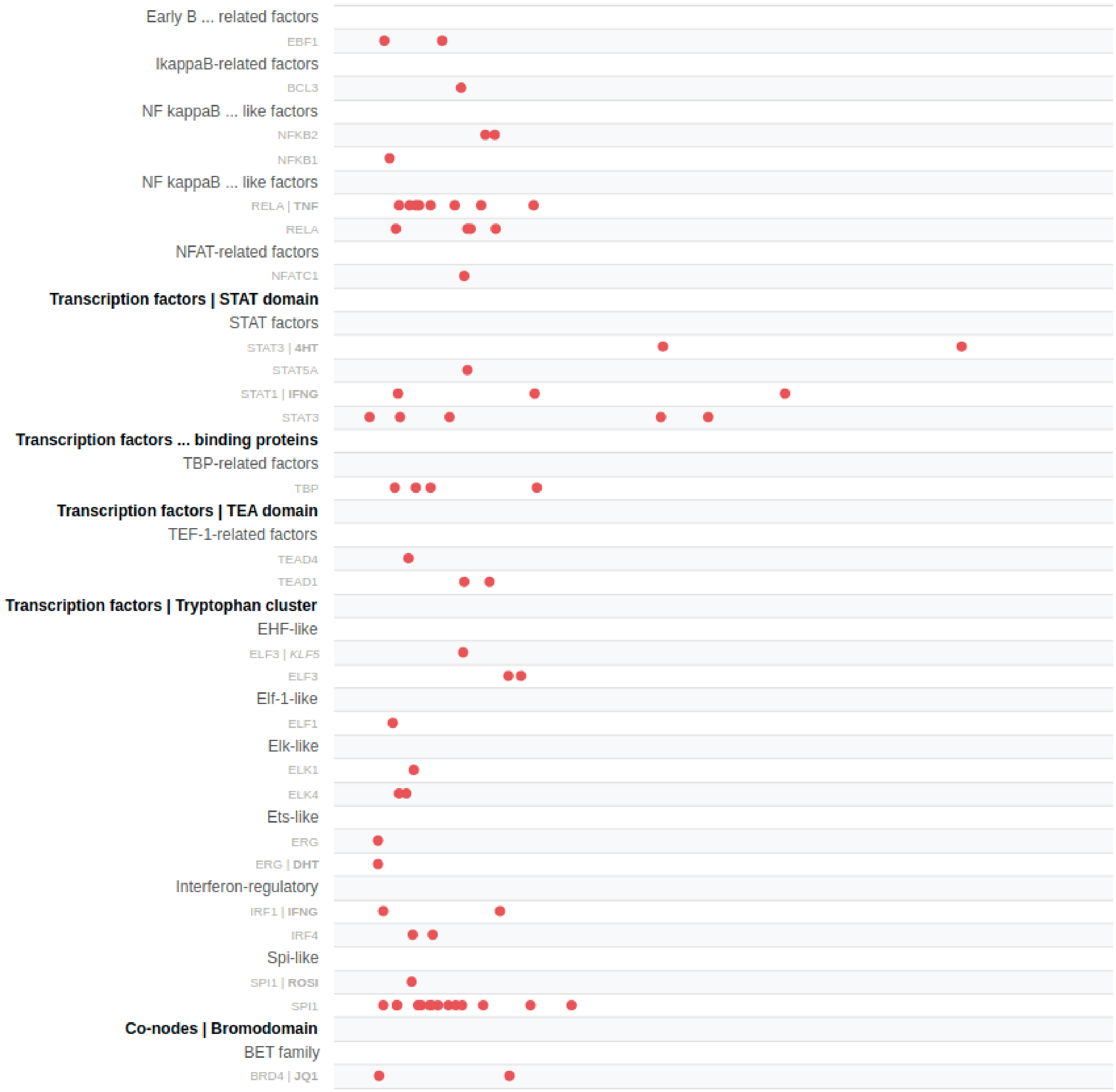
Early B ... related factors
EBF1
IkappaB-related factors
BCL3
NF kappaB ... like factors
NFKB2
NFKB1
NF kappaB ... like factors
RELA | TNF
RELA
NFAT-related factors
NFATC1
Transcription factors | STAT domain
STAT factors
STAT3 | 4HT
STAT5A
STAT1 | IFNG
STAT3
Transcription factors ... binding proteins
TBP-related factors
TBP
Transcription factors | TEA domain
TEF-1-related factors
TEAD4
TEAD1
Transcription factors | Tryptophan cluster
EHF-like
ELF3 | KLF5
ELF3
Elf-1-like
ELF1
Elk-like
ELK1
ELK4
Ets-like
ERG
ERG | DHT
Interferon-regulatory
IRF1 | IFNG
IRF4
Spi-like
SPI1 | ROSI
SPI1
Co-nodes | Bromodomain
BET family
BRD4 | JQ1
