# Supplementary material for: MUC16 and TP53 family co-regulate tumor-stromal heterogeneity in pancreatic adenocarcinoma
Source: Front Oncol. 2023 Feb 3;13:1073820. doi: 10.3389/fonc.2023.1073820 (PMC9936860; doi:10.3389/fonc.2023.1073820)
Supplement: Supplementary file 7 [file DataSheet_4.pdf]

Supplementary Methods-1

Additional details on data processing and lineage tracing analysis of scRNA-seq data of KPC stromal cells

The RNA-seq processed data of tumor-stromal subtypes (also known as Moffitt data; n = 357) was retrieved from the NCBI GEO repository (accession no: GSE71729). Only primary PDAC samples (n = 145) were used for downstream analysis. The prior normalized scRNA-sdata of stromal cells (DAPI-, CD45-, CD31-, EPCAM-) (GSE129455) from 4 KPC (Kras-LSL-G12D; Trp53-LSL-R172H; Pdx1-Cre) C57BL6/J mice were used to perform clustering and lineage trajectory analysis. The exploratory clustering (unsupervised) of stromal cells revealed the presence of distinct CAF subpopulations characterized by either myeloid-like (Cd14-expressing), myoepithelial/smooth muscle (SMC) (Caldesmon/Cald1), and mesothelial (MUC16, Upk3b, Upk1b) transcriptional activity. We also identified the expression of mesothelin, a well-established marker of mesothelial cells, in ~40% of all KPC stromal cells, including but not limited to mesothelial cells and fibroblasts. As this was not the case with other specific mesothelial markers like Muc16 and Uroplakins (Upk1b and Upk3b), we investigated if mesothelin-expressing stromal cells (fibroblasts and epithelioid-like cells) share lineage with mesothelial cells using trajectory inference analysis. To perform this analysis, we first separated the stromal cells individually based on the expression of Mesothelin (Msln) (3370 cells), Muc1/epithelial membrane antigen (938 cells) or Muc16 (195 cells). As these cell groups did not show mutual exclusivity (**Supplementary Methods Figure 1**), we made distinct (mutually-exclusive) cell groups using the following considerations: a) identification of mesothelial cells (146 cells) with 5 specific markers (Wt1, Msln, Upk3b, Upk1b, Muc16) instead of just Muc16; b) Next, we grouped Muc1-expressing cells (921 cells) irrespective of expression of mesothelin because most of these cells did not express any of the mesothelial markers except Msln besides their distinctive epithelial (E-cadherin expression) and myeloid (Cd14-exp) like transcriptional activity (**Supplementary Methods Figure 2 & 3**); c) Lastly, we selected the rest of the mesothelin-expressing cells (2491 cells) to create the third group. The cell trajectory analysis was performed using

Monocle3 R package. The mesothelial cells expressing highly selective markers (Wt1, Muc16, Msln, Upk3b, Upk1b) were selected as root cells for trajectory inference.

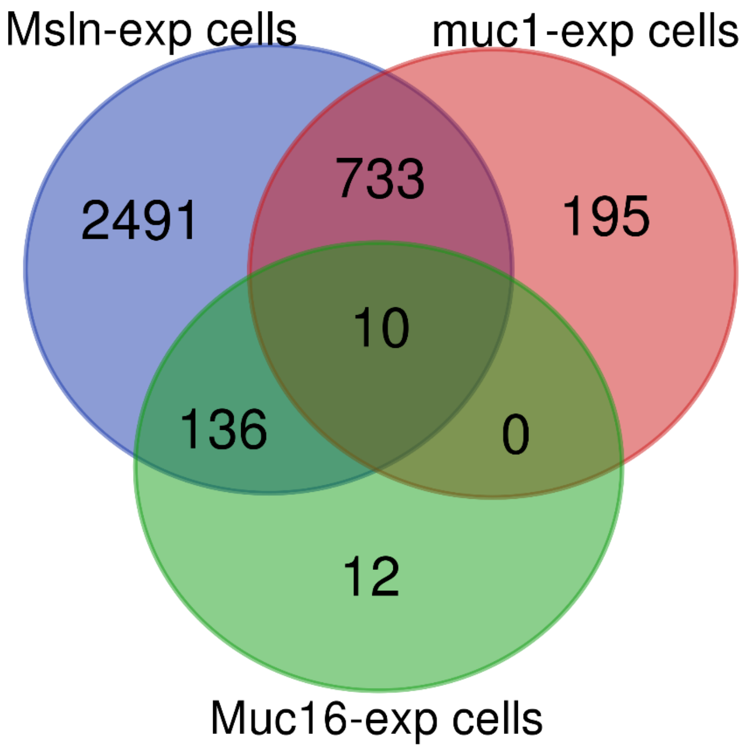

Supplementary Methods Figure 1: Venn diagram showing the overlap of cells between the groups initially created using the expression of Msln, Muc1, and Muc16 without additional filters.

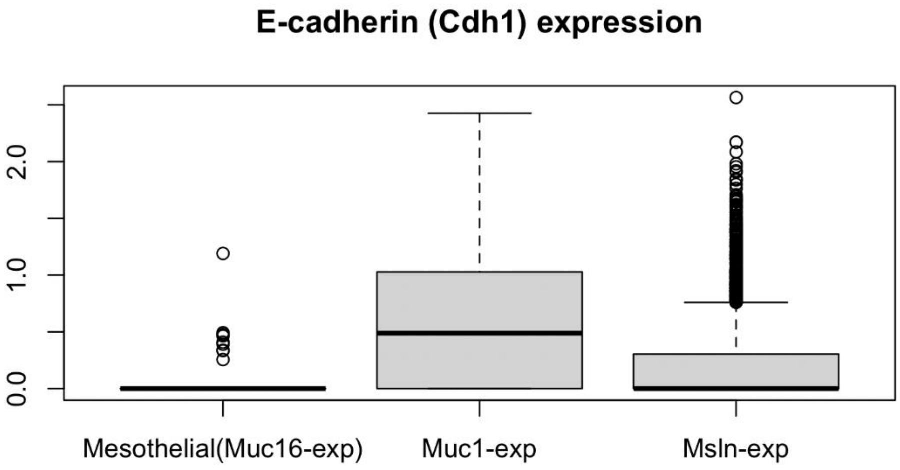

Supplementary Methods Figure 2: Boxplot illustrating the differences in the expression of E-cadherin (cdh1) between Mesothelial (Muc16-exp), Muc1-exp and Msln-exp cell groups.

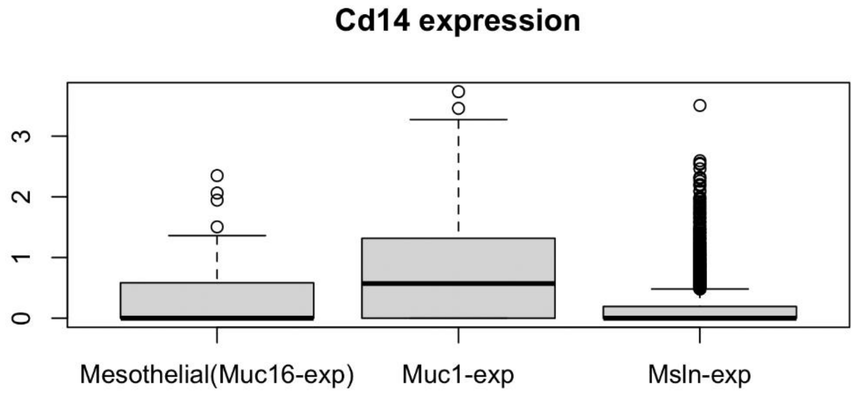

Supplementary Methods Figure 3: Boxplot illustrating the differences in the expression of myeloid marker Cd14 between Mesothelial (Muc16-exp), Muc1-exp and Msln-exp cell groups.
